# Genetic Loci Controlling Carotenoid Biosynthesis in Diverse Tropical Maize Lines

**DOI:** 10.1534/g3.117.300511

**Published:** 2018-01-29

**Authors:** Girum Azmach, Abebe Menkir, Charles Spillane, Melaku Gedil

**Affiliations:** * Maize Breeding and Genetics Division, Bako National Maize Research Center, Ethiopian Institute of Agricultural Research, Bako, Ethiopia; † Genetics and Biotechnology Laboratory, Plant and AgriBiosciences Research Centre, Ryan Institute, National University of Ireland Galway, H91 REW4, Ireland; ‡ International Institute of Tropical Agriculture, PMB 5320, Ibadan, Nigeria

**Keywords:** vitamin A deficiency, provitamin A, linkage disequilibrium (LD), mixed linear model (MLM), genotyping by sequencing (GBS), biofortification, maize

## Abstract

The discovery and use of genetic markers associated with carotenoid levels can help to exploit the genetic potential of maize for provitamin A accumulation more effectively. Provitamin A carotenoids are classes of carotenoids that are precursors of vitamin A, an essential micronutrient in humans. Vitamin A deficiency is a global public health problem affecting millions of people, especially in developing countries. Maize is one of the most important staple crops targeted for provitamin A biofortification to help alleviate vitamin A deficiency in developing countries. A genome-wide association study (GWAS) of maize endosperm carotenoids was conducted using a panel of 130 diverse yellow maize tropical inbred lines genotyped with Genotyping by Sequencing (GBS) SNP markers. Numerous significant association signals co-localizing with the known carotenoid biosynthesis genes *crtRB1*, *lcyE* and *ZEP1* were identified. The GWAS confirmed previously reported large effects of the two major carotenoid biosynthesis genes *lcyE* and *crtRB1*. In addition, significant novel associations were detected for several transcription factors (*e.g.*, RING zinc finger domain and HLH DNA-binding domain super family proteins) that may be involved in regulation of carotenoid biosynthesis in maize. When the GWAS was re-conducted by including the major effects of *lcyE* and *crtRB1* genes as covariates, a SNP in a gene coding for an auxin response factor 20 transcription factor was identified which displayed an association with β-carotene and provitamin A levels. Our study provides a foundation for design and implementation of genomics-assisted selection strategies for provitamin A maize breeding in tropical regions, and advances efforts toward identification of additional genes (and allelic variants) involved in the regulation of carotenoid biosynthesis in plants.

Carotenoids are diverse organic pigments that are naturally found in plants and other organisms (Cazzonelli 2011; Moran and Jarvik 2010). The β-ionone ring(s) containing carotenoids, known as provitamin A carotenoids (*e.g.*, β-carotene, β-cryptoxanthin and α-carotene), are precursors of the essential micronutrient vitamin A in humans (Fraser and Bramley 2004; West and Darnton-Hill 2008). However, humans cannot synthesize vitamin A *de novo*, and therefore need to obtain the nutrient from dietary sources either as preformed vitamin A (retinol) from animal-based foods (*e.g.*, liver, whole milk, and egg), and/or as precursors of vitamin A from colored vegetables and fruits (*e.g.*, carrots, dark green leaves and papaya) in the form of provitamin A carotenoids (West and Darnton-Hill 2008).

Vitamin A deficiency is a global public health problem. The World Health Organization (WHO) estimates that 190 million pre-school children and 19 million pregnant women worldwide were vitamin A deficient (in the period 1995–2005) with a prevalence rate of 33 and 15%, respectively, based on low serum retinol content (<0.7 μmol/liter) (World Health Organization 2009). Almost half a million children lose their sight every year due to xerophtalmia caused by vitamin A deficiency, the leading cause of preventable blindness (Sherwin *et al.* 2012). Millions of child deaths annually are attributed to vitamin A deficiency coupled with other undernutrition problems (Black *et al.* 2003).

Genetic improvement of staple crops for improved nutritional quality (*e.g.*, enhanced level of micronutrients) has been termed biofortification and is a promising approach for reducing vitamin A and other micronutrient deficiencies in human populations. Maize represents a significant proportion of the total calorie intake of people in many African countries, accounting for ∼30% of the per-capita calorie consumption in Eastern and Southern Africa, even reaching as high as 56% in some of the southern African countries (FAO, 2011). The biofortification of maize with higher levels of provitamin A carotenoids could play a significant role in reducing vitamin A deficiency in regions where maize is a major staple crop (Wurtzel *et al.* 2012; Burt *et al.* 2011; Meyers *et al.* 2014). While breeding lines of maize that can accumulate up to 26 μg/g β-carotene (and 30 μg/g of provitamin A carotenoids) in the endosperm have been reported (Pixley *et al.* 2013), commonly cultivated maize varieties contain low levels of provitamin A carotenoids ranging from 0.5 to 1.5 μg/g (Harjes *et al.* 2008).

Understanding the genetic variation, genes and regulatory mechanisms controlling maize endosperm carotenoid levels is important for biofortification of maize with high levels of provitamin A carotenoids. Genome wide association study (GWAS) approaches are a powerful approach for ascribing gene-phenotype relationships (Huang and Han 2014; Yu and Buckler 2006; Zhu *et al.* 2008), while genotyping by sequencing (GBS) is a next-generation sequencing (NGS) based genotyping approach that has dramatically facilitated large-scale genome-wide marker development and GWA studies in crop species (Chia *et al.* 2012; Davey *et al.* 2011; Elshire *et al.* 2011; Varshney *et al.* 2014).

A number of GWA studies have identified loci controlling agronomic traits such as plant height, yield and yield components, flowering time and plant architecture in a range of crops, including barley (Pasam *et al.* 2012), tomato (Shirasawa *et al.* 2013), wheat (Edae *et al.* 2014; Wang *et al.* 2014), and maize (Thornsberry *et al.* 2001; Wang *et al.* 2012). GWA studies have also identified loci associated with grain quality traits including oil content in maize (Li *et al.* 2013), protein contents in wheat (Edae *et al.* 2014; Wang *et al.* 2014) and essential micronutrients such as α-tocopherol (vitamin E) and β-carotene in maize (Harjes *et al.* 2008; Li *et al.* 2012; Lipka *et al.* 2013; Yan *et al.* 2010). In particular, two key carotenoid biosynthesis genes, namely *LCYE* and *crtRB1* (*HYD3*), have been found to be significantly associated with accumulation of provitamin A carotenoids in maize endosperm (Harjes *et al.* 2008; Yan *et al.* 2010). Different allelic variants of these genes can affect the flux of substrates through the carotenoid biosynthesis pathway leading to synthesis of higher levels of provitamin A carotenoids (*e.g.*, β-carotene). The total provitamin A carotenoid proportion in maize endosperm is affected by the level of total carotenoids accumulated in the endosperm which is a function of substrate flux into the carotenoid pathway and downstream catabolic steps involving degradation of carotenoids (Rodríguez-Concepción 2010; Vallabhaneni *et al.* 2010)

There have been only a few GWA studies for carotenoid composition and content in maize endosperm to date (Owens *et al.* 2014; Suwarno *et al.* 2015). In our study, we have used a GBS-based GWA approach to identify loci associated with carotenoid content and composition of maize endosperm. Uniquely, our study used a collection of genetically diverse yellow maize inbred lines (with a mixed genetic background of both tropical and temperate germplasm) developed by the maize breeding program of the International Institute of Tropical Agriculture (IITA). In addition, we factored in the effect of already identified provitamin A alleles as covariates to detect additional association signals. Our findings contribute to ongoing efforts to identify allelic variants that can be used for genomic selection to develop maize lines with higher levels of provitamin A carotenoids.

## Materials and Methods

### Germplasm used for GWAS

A panel of 130 diverse yellow maize inbred lines previously described in Azmach *et al.* (2013) was employed for the GWAS. This panel had inbred lines with kernel colors ranging from light yellow to dark orange. White maize lines were not included, since our focus was to investigate the genetic variability underlying composition and content of the various carotenoids in maize endosperm. We did not investigate the variation between white and yellow lines, which is largely determined by a mutation in the *psy*1 locus (Palaisa *et al.* 2003). This maize germplasm panel was composed of inbred lines developed by IITA from eight bi-parental crosses, four broad based populations, and 28 backcrosses of tropical inbred lines, involving five temperate lines as donors of high β-carotene alleles (Azmach *et al.* 2013). The inbred lines were considered to represent the allelic diversity underlying the variation in carotenoid composition and content in both the temperate and tropical maize gene pools, since each line contained both tropical and temperate maize germplasm in its genetic background.

### Field trial evaluation and analysis of carotenoids

Field trial evaluation of the maize inbred lines was performed at IITA’s research station, Ibadan, Nigeria (7°29′11.99″N, 3°54′2.88″E, altitude 190 m above sea level) for two seasons, in 2010 and 2011. The trial was arranged in (10,13) alpha-lattice design with two replications. Each line was planted in a 5 m row plot, with 0.75 m spacing between rows and 0.25 m within each row. The fields were managed as per the recommended agronomic practices (Menkir *et al.* 2008) which included fertilization at the rates of 60 kg N, 60 kg P, and 60 kg K ha^1^ at the time of sowing, with an additional 60 kg N ha^1^ applied as top dressing 4 wk later; plus weed control using Primextra and Gramazone herbicides applied as pre-emergence herbicides each at 5 liter ha^1^. Subsequent manual weeding was done to keep the trials weed-free. The environmental conditions during the first season were as follows: Total rainfall of 310 mm (supplemented with irrigation); temperature ranged from 19.4 to 33.8° with average 27.7°; relative humidity ranged 28–97% with average 67%; and solar radiation ranged from 19.4 to 21.2 MJ/m^2^/d. During the second season the total rainfall was 1022.5 mm; the temperature ranged from 21.7 to 32.4° with average 25.8°; the relative humidity ranged from 40 to 97% with average 78%; and the solar radiation was from 15.2 to 20.2 MJ/m^2^/d. The dominant soil type of the trial site is Ferric Lixisols (FAO 1991), which is a sandy loam soil, moderately drained with a PH of 6.2.

Seed samples for carotenoid analysis were generated by controlled self-pollination of all plants in each plot. The self-pollination protocol employed consisted of covering the shoots with shoot bags before emergence of the silks to avoid cross pollination, once the shoots were ready for pollination, the tassels were bagged with pollination bags a day before pollination. The next day fresh pollen was collected and applied on the silks of the same plant using the pollination bags, after which the shoots were covered with the same bag used for self-pollination. The shoots remained bagged until harvesting. The ears of each self-pollinated maize line in each plot were harvested, dried under ambient temperature with minimal exposure to direct sunlight, and separately shelled. Samples of 100 kernels were used from each seed lot for carotenoid analysis.

The carotenoids from kernel samples of each of the 130 maize inbred lines were extracted and quantified with HPLC at the University of Wisconsin, USA. The extraction protocol used was the method of Howe and Tanumihardjo (2006) for carotenoid analysis of dried maize kernels, as previously described in Azmach *et al.* (2013). Extraction was performed using finely ground 0.5 g samples of each inbred line’s kernels. The internal standard consisted of 200 μl of β-Apo-8’-carotenal (Sigma-Aldrich, St. Louis, MO), which was added at the beginning of the analysis for calibrating losses of carotenoids during extraction and the entire work-flow process. Fifty microliter aliquots of each extract were injected into the HPLC system (Waters Corporation, Milford, MA). The gradient was applied for 30 min from 70% solvent A:30% solvent B, to 40% solvent A:60% solvent B. Each carotenoid type was quantified based on calibrations using its respective external standard. Total carotenoid content was calculated as the sum of concentrations of α-carotene, lutein, β-carotene, β-cryptoxanthin, zeaxanthin. Provitamin A was calculated by summing the concentrations of β-carotene, and half concentrations of each of β-cryptoxanthin and α-carotene, since β-cryptoxanthin and α-carotene can provide only one molecule of retinol each as opposed to two molecules of retinol for β-carotene (US Institute of Medicine 2001). Other derived carotenoid traits were also calculated as indicated in Harjes *et al.* (2008), Yan *et al.* (2010): *i.e.*, ratio of carotenoids in β to α branch of the carotenoid pathway, ratio of β-carotene to β-cryptoxanthin and ratio of β-carotene to all carotenoids (β-carotene + α-carotene + lutein + zeaxanthin + β-cryptoxanthin). The data for the ratio traits were transformed using natural logarithm (log_e_) before being subjected to statistical analysis to correct for the non-normal distribution of the data. All carotenoid concentrations were measured in microgram/gram dry weight (DW). BLUEs (best linear unbiased estimates) calculated for each trait based on the two season carotenoid data were used in the GWAS. BLUEs were calculated using the GLM option of TASSEL software version 4 (Bradbury *et al.* 2007) with a statistical model Y = Xβ + e, where Y is matrix of the dependent or response variables, *i.e.*, each carotenoid type; X is the design matrix; β is vector of fixed effect parameters, and e is vector of the random errors that are assumed to be normally distributed and independent of the other variances.

### Genome wide SNP marker generation using GBS

DNA samples were isolated from freeze-dried leaf samples of each inbred line using Qiagen DNeasy plant mini kit following the protocol supplied with the product. DNA samples were quantified using a NanoDrop 2000 Spectrophotometer. Samples having at least 10 ng/µl DNA each were prepared and sent to the Genome Diversity Facility (GDF), formerly Institute for Genomic Diversity (IGD), Cornel University, USA, for GBS genotyping. Genotyping by sequencing (GBS) libraries were prepared, analyzed and sequenced at GDF, according to Elshire *et al.* (2011). SNP calling from the sequenced GBS library was also performed at GDF using the GBS production pipeline (Version: 3.0.134), an extension of the Java program TASSEL (Bradbury *et al.* 2007; Glaubitz *et al.* 2014) which used aligned short reads of GBS (tags). The GBS pipeline options used for calling SNPs consisted of: 0.1 minimum locus coverage, 1 × 10^6^ maximum number of SNPs per chromosome, duplicate SNPs above 0.05 mismatch rate were not merged, and 0.8 cutoff frequency between heterozygote *vs.* homozygote calls. Tags were aligned to the reference genome B73 refgen_v2 (Schnable *et al.* 2009).

The GBS pipeline generated a data set containing a total of 619,596 unfiltered SNPs. This SNP dataset had a total of 51% missing data points possibly caused by biological presence-absence of sequences between the reference and each test genome, or errors introduced in the GBS procedures (Glaubitz *et al.* 2014; Poland and Rife 2012). The dataset was further filtered in TASSEL 4 on the basis of missing data proportion and minor allele frequency (MAF) cutoff thresholds (Bradbury *et al.* 2007). The cutoff thresholds used to filter the dataset for the GWAS allowed only those SNPs showing a maximum of 20% missing data, and 1% minimum MAF (MMAF). This resulted in a dataset of 109,937 SNPs. The diversity and genome-wide Linkage Disequilibrium (LD) analysis were performed using datasets obtained by filtering with criteria of no missing data points and 1 and 10% MAFs which resulted in 3532 and 1658 genome-wide SNPs, respectively. SNP data summary and basic diversity parameters were calculated using TASSEL 4 (Bradbury *et al.* 2007) and PowerMarker 3.25 (Liu and Muse 2005) softwares.

### Linkage disequilibrium (LD) estimation

The two commonly used measures of LD are Lewontin’s D and the squared pairwise correlation coefficient *R*^2^ (Chen *et al.* 2006; Flint-Garcia *et al.* 2003). Although D’ is a good measure of recombination history, it is severely affected with reduced sample size. *R*^2^ summarizes both recombination and mutation history (Flint-Garcia *et al.* 2003). In our study, LD was estimated using *R*^2^, since it helps detect LD with minimal error despite small sample size and low MAF (Khatkar *et al.* 2008; Yan *et al.* 2009). In addition *R*^2^ is a more relevant measure of LD for conducting association analysis between genotype and traits (Flint-Garcia *et al.* 2003).

To determine the degree of resolution achieved in the association analysis (Yu and Buckler 2006), both genome and chromosome wide linkage disequilibrium (LD) were estimated using the squared allele frequency correlation coefficient (*R*^2^) for all possible pairs of SNPs in a dataset. For genome-wide LD, SNP datasets of the 10 maize chromosomes were combined and filtered with cut-off threshold of no missing data and 10% MMAFs yielding 1658 SNPs typed across all inbred lines. On the other hand, LD estimation within each chromosome was performed using the SNP data of each chromosome filtered at 10% maximum missing data per marker and 10% MMAFs. Missing data in all the SNP datasets used for chromosome wide LD analysis were not imputed. The software used to estimate LD was TASSEL 3 (Bradbury *et al.* 2007), which uses permutation tests to determine the *P*-values for each pairwise correlation. LD estimate significance levels were considered at α = 0.001 (Pasam *et al.* 2012). Genome-wide and chromosome wide rate of LD decays were estimated by plotting localized regression curves (LOESS) of the *R*^2^ values *vs.* the corresponding physical distances between the SNP pairs, followed by observation of the intersection point between the fitted LOESS curve and a critical *R*^2^ values (Cleveland and Devlin 1988; Breseghello and Sorrells 2006). Two background critical *R*^2^ values for estimating LD decays within and across chromosomes were considered in the present study to offer comparison. The first baseline critical *R*^2^ was determined by taking the parametric 95 percentile of distribution of *R*^2^ values for unlinked SNPs, taking SNPs on different chromosomes and SNPs beyond 50 Mbp apart on the same chromosome as unlinked (Breseghello and Sorrells 2006; Pasam *et al.* 2012). The second baseline *R*^2^ value was 0.2, an arbitrary value often used to describe LD decay (Zhu *et al.* 2008). Scatter plots and fitted smooth curves for estimating LD decay were plotted using a base scatter plot function of R version 3.0.3, “scatter.smooth” (R Core Team 2014). The function plots and adds a smooth curve to a scatter plot computed according to LOESS (R Core Team 2014). LD patterns of all SNPs significantly associated with carotenoids and local LD patterns in regions surrounding significant genes were visualized using LD plots generated with HaploView (Barrett *et al.* 2005).

### Genome wide SNP-trait association

Associations between genome-wide SNPs and carotenoid content was identified using the R (R Core Team 2014) package GAPIT (Genetic Association and Prediction Integrated Tools) (Lipka *et al.* 2012, 2013). GAPIT package uses a unified mixed linear model (MLM) to calculate genome-wide association between traits and large number of markers by employing methodologies that maximize statistical power, provide high prediction accuracy, and run in a computationally efficient manner (Kang *et al.* 2010; Lipka *et al.* 2012, 2013; Yu *et al.* 2006; Zhang *et al.* 2010). A unified MLM incorporates both population structure (Q) and relative kinship (K) inferred from marker data into the GWAS to control for the confounding effect of Q and K and thus minimize spurious associations due to both type I and type II errors (Yu *et al.* 2006). Since the panel used in this study was composed of groups of inbred lines that were extracted from many backcrosses and single crosses involving diverse parental germplasm, multiple level relative kinship and non-random population structure was expected. Thus, the unified MLM model was applied to compute accurate associations. The analysis was executed mainly with the default settings of the software which automatically calculated both K and Q using the entire SNP marker data. The default setting implements VanRaden’s algorithm option (VanRaden 2008) to calculate the K matrix, and uses principal component analysis (PCA) to define Q. It applies optimum compression levels using default kinship clustering and grouping values “average” and “mean,” respectively. The model selection option was used to estimate the optimum number of principal components (PC) covariates using Bayesian Information Criterion (BIC) (Schwarz 1978). The variation explained for a trait by the model and a particular SNP in question were determined using the likelihood *R*^2^ statistics calculated in GAPIT.

SNP data used for GWAS was filtered in TASSEL 4 with maximum missing data of 20% and MMAF of 1%. Missing data were imputed automatically within GAPIT using the conservative option of “major allele,” which replaces missing data points with the major allele of the SNP. Different significance cut-off thresholds were used to assess the effect of the SNPs on carotenoids. The statistical significances of the SNPs were evaluated at 5 and 1% critical thresholds of the false discovery rate (FDR) adjusted *P*-values (Benjamini and Hochberg 1995) and the Bonferroni procedure was used to control the experimentwise type I error rate at both α = 0.05 and α = 0.01. FDR values generated with the GWAS result in GAPIT were used.

### GWAS including allele specific markers of lcyE and crtRB1 as covariates

Variations in carotenoid content and composition of the association panel caused by allelic variants in the two genes, *lcyE* (Harjes *et al.* 2008) and *crtRB1* (Yan *et al.* 2010) were accounted for by including marker score data of the three allele-specific markers of each gene as covariates. These marker data were scored in the same inbred line panel used in the current study to validate the allele specific markers by Azmach *et al.* (2013). To incorporate these markers into the GWAS the six allele specific marker data were first transformed to principal components. Components explaining the largest proportion of the variation were then included as covariates in the unified mixed model for calculating the second GWA using GAPIT.

### Data availability

The genotype and phenotype data for this GWAS population are available in Supplemental Material, File S1 (hapmap file containing GBS generated SNP data for the 130 maize inbred lines, filtered with maximum of 20% missing data and 1% minimum allele frequency) and File S2 (an excel file containing BLUEs of carotenoid contents for the 130 maize inbred lines).

## Results

### Carotenoid profile

The carotenoid composition and content of maize lines used for this GWA study has been described in Azmach *et al.* (2013). The panel displayed considerable diversity in carotenoid profile. The ranges of least square means of the carotenoid concentrations (over two growing seasons) are presented in Table S3 in File S3 (can also be referred in detail in Azmach *et al.* (2013)). The BLUEs of the carotenoids are available in File S1. The concentration of α-carotene was low across the inbred lines, with poor repeatability, and hence α-carotene was not included in the GWA study.

### SNP diversity

The summary of the 110 k SNP data set used for the GWAS and its diversity parameters are presented in Table 1. The average missing data for this data set was 10%. SNP distribution across the genome was not uniform but attained significant coverage (Figure S1). The MAF displayed a uniform distribution across the 10 maize chromosomes (average = 0.13–0.14, median = 0.06–0.8). The rare allele frequencies (<0.05) represented the largest proportion of the MAFs (Figure S2). The average inbreeding coefficient (f) estimates per locus ranged from below zero to one, while the genome-wide mean f was 0.82. The heterozygosity (H) of the lines varied from 0.02 to 0.13, with an average of 0.05. More than half of the inbred lines showed <0.04 H values. Both H and f had more or less uniform values across the chromosomes. The genome-wide polymorphic information content (PIC) of the SNPs ranged from 0.02 to 0.38, while the average was 0.18. PIC is one of the diversity parameters that is used to measure the informativeness of genetic markers. A large proportion (>40%) of SNPs used for diversity analyses in this study had PIC values higher than 0.2, suggesting informativeness of the GBS generated SNPs for the association study.

**Table 1:** Summary of extent of chromosome and genome-wide LD estimates plus information on the SNP datasets used to calculate the LD

Chromosome	No. of SNPs	No. of pairwise Comparisons	*R*^2^				LD decay*^a^*	
			% *P* < 0.001	% >0.2	Avg	Max	*R*^2^ = 0.2	*R*^2^ = 0.25
1	3710	6,878,340	13.8	28.6	0.18	1	0.96	0.71
2	2790	2,889,260	12.4	30.8	0.19	1	0.65	0.59
3	2384	2,839,344	20.0	35.6	0.20	1	1.6	0.71
4	2294	2,628,924	13.2	29.8	0.19	1	0.71	0.71
5	2617	3,423,036	14.6	32.6	0.20	1	0.71	0.71
6	1644	1,349,724	12.1	31.0	0.19	1	0.83	0.71
7	1750	1,529,500	21.9	34.0	0.20	1	1.27	0.56
8	1851	1,712,175	16.0	32.5	0.20	1	0.71	0.59
9	1834	1,679,944	13.0	30.3	0.19	1	0.83	0.77
10	1562	1,218,360	13.0	32.5	0.20	1	0.71	0.65
Across genome	1658	1,373,654	10.25	15.40	0.25	1	0.83	0.65

a LD decay estimated at two baseline critical *R*^2^ values for comparison purpose. Avg, average; Max, maximum; % *P <* 0.001, percentage of significant LD (*R*^2^) having their *P*-values <0.001; % >0.2, percentage of *R*^2^ values >0.2 critical *R*^2^.

### Population structure, kinship and LD

**Figure 1:**
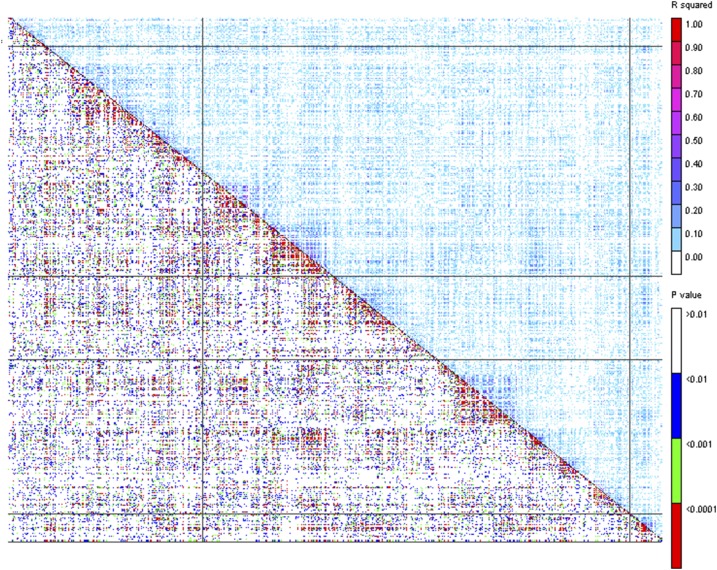
Genome-wide LD heat-map plotted using 1658 SNP dataset representing all the 10 chromosomes of maize in TASSEL 3 software. The lower triangle represents *P*-values while the upper one represents R2. The color legends indicate the level of significance and the corresponding strength of LD (R2).

The Bayesian Information Criteria (BIC) suggested the population structure calculated (based on PCA) had only a small contribution to the variation in carotenoid profile of the panel (Table S2 in File S3). The kinship heat map indicated a low level of overall relatedness in the panel (Figure S3). The genome-wide extent of LD estimate was 0.83 Mbp at baseline *R*^2^ = 0.2 and 0.65 Mbp at *R*^2^ = 0.25 (Figure S4, Supplemental Information in File S3, and Table 1). There was heterogeneous distribution of LD decay across the genome, as was evident from the pattern of LD heat-map generated using the same SNP dataset (Figure 1).

### GWA analysis of carotenoid content and composition

Of the 110 k SNPs tested, 386 unique significant SNPs were detected at 5% FDR (Table 2). At this significance threshold, at least two significant SNPs were identified on each of the 10 chromosomes. The number of significant SNPs declined to 168 at 1% FDR correction rate, discarding all the significant SNPs on chromosomes 1, 5 and 7. Application of the conservative multiple comparison correction term, the Bonferroni test, at 5 and 1% levels further reduced the number of significant SNPs to 81 and 32, respectively. The vast majority of significant SNPs were found on chromosome 8 followed by chromosome 10, which were mainly associated with lutein and β-branch carotenoids, respectively. Except for significant SNPs on chromosome 6 and 9, the average MAFs of the significant SNPs at FDR 1 and 5% were above 10%. Only 5% of the significant SNPs at FDR 1% had their MAFs below 10% (Figure 1).

**Table 2:** Number of significant SNPs by chromosome with the corresponding average minor allele frequencies (MAFs) at different significance cutoff thresholds

Chromosome	FDR		Bonferroni		Average MAF	
	5%	1%	5%	1%	FDR 5%	FDR 1%
1	7	0	0	0	0.15	—
2	14	4	3	0	0.21	0.20
3	7	3	0	0	0.16	0.18
4	4	3	1	1	0.13	0.14
5	5	0	0	0	0.14	—
6	7	1	0	0	0.13	0.03
7	3	0	0	0	0.10	—
8	226	120	53	27	0.20	0.19
9	4	1	0	0	0.09	0.08
10	109	36	24	13	0.21	0.21
All*^a^*	386	168	81	41	0.18	0.19

a SNPs associated with multiple trait counted only once; FDR, false discovery rate; MAF, minor allele frequency.

**Figure 2:**
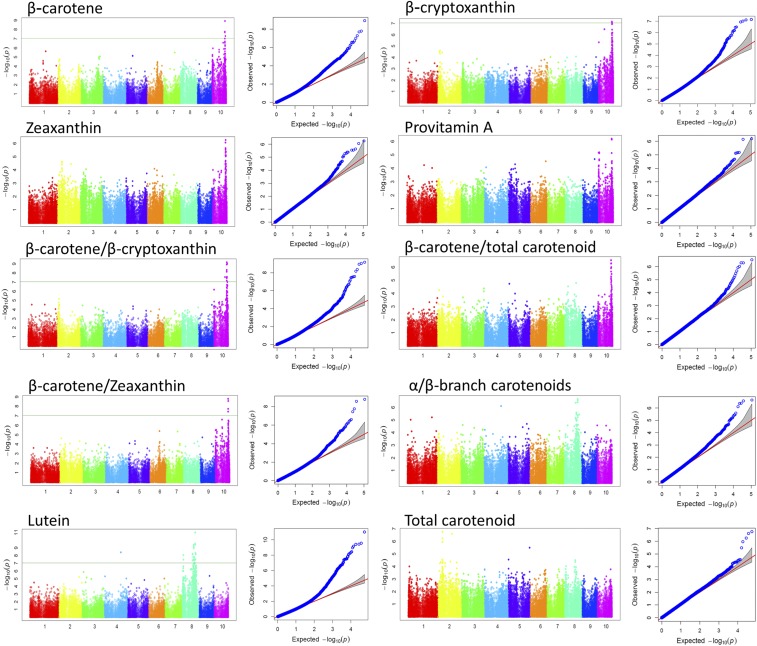
Manhattan and QQ-plots depicting the GWAS results. For the Manhattan plots, horizontal axes represent chromosomes, vertical axes represent −log of the *P*-values to the base 10. Horizontal line at −log10(p) = 7.04 is 1% Bonferroni-adjusted cutoff threshold for highly significant associations. QQ-plots (quantile quantile-plots) show how well the MLM GWAS model fit expectation; they are the −log of *P*-values from the MLM GWAS in the *y*-axis plotted against their expected values under the null-hypothesis of no association between SNPs and the trait under consideration in the *x*-axis (Lipka *et al.* 2012).

The number of significant SNPs in relation to each carotenoid across each chromosome is summarized in Table 3. The allelic variants and effects selected for the most significant SNPs in the GWAS are indicated in Table 4, while the associated candidate protein coding genes along with their genomic positions are listed in Table 5. Figure 2 illustrates the GWAS result for each carotenoid trait, complemented by Figure S5 which summarizes the association using the lowest *P*-values attained at 5% FDR threshold. The strongest association was detected for lutein content. At the significance level of 1% FDR, a total of 129 SNPs distributed on chromosomes 2, 3, 4, 6, 8 and 9 were associated with lutein levels, with the largest fraction of SNPs (>90%) located on chromosome 8. The most significant SNPs associated with this carotenoid scored the lowest of all the *P*-value (SNPs S8_138938983 and S8_138938949, *P* = 9.81E−12). The model containing each of these SNPs explained 53% of the variation in accumulation of this carotenoid. The majority of significant SNPs that survived the stringent significance threshold of 1% Bonferroni were also associated with lutein (27 SNPs on chromosome 8). Many of these SNPs were also associated with the ratio of α- to β-branch carotenoids at FDR 5%. The second most significant association was detected for the ratio of β-carotene to β-cryptoxanthin derived carotenoid trait. Twenty six SNPs were associated with this derived trait at FDR 1%, the most significant SNP (S10_136007578) scoring *P*-value of 6.75E−10 and *R*^2^ of 60%.

**Table 3:** Number of significantly associated SNPs with carotenoids by chromosome

Carotenoid	Significance Threshold	No. of SNPs (Chromosome)	Total
β-carotene	Bon 1%	5 (10)	5
	FDR 1%	17 (10)	17
β-cryptoxanthin	Bon 1%	2 (10)	2
	FDR 1%	12 (10)	12
Lutein	Bon 1%	27 (8)	27
	FDR 1%	1 (2), 3 (3), 3 (4), 1 (6), 120 (8), 1 (9)	129
β-carotene to β-cryptoxanthin ratio	Bon 1%	12 (10)	12
	FDR 1%	26 (10)	26
α to β branch carotenoids ratio	FDR 1%	5 (8)	5
	FDR 5%	10 (8)	11
Zeaxanthin	FDR 5%	12 (10)	12
Provitamin A	FDR 5%	3 (10)	3
Total carotenoid	FDR 1%	3 (2)	3
β-carotene to zeaxanthin ratio	Bon 1%	5 (10)	5
	FDR 1%	12 (10)	12
β-carotene to all carotenoids ratio	FDR 5%	8 (10)	8

SNPs associated with multiple carotenoids were included in the counts in this Table. Hence, sums of SNPs may not tally with those indicated in Table 2. FDR thresholds of 5% were considered only for traits for which no significant SNPs could be obtained at the stringent thresholds. Bon, Bonferroni; FDR, false discovery rate.

**Table 4:** Summary of significant SNPs identified in the GWAS study without considering the allele specific markers as covariates

No.	SNP*^a^*	Alleles	Allelic Effect	*P*-value	MAF	*R*^2^ of Model	*R*^2^ of Model + SNP	FDR adjusted *P*-value	Carotenoid
1	S2_208672678	C/G	3.00	6.17E−06	0.03	0.16	0.31	6.40E−03	lut
2	S3_18632237	C/G	1.76	1.38E−06	0.23	0.16	0.33	2.10E−03	lut
3	S3_49624005	C/T	2.24	7.05E−06	0.13	0.16	0.31	6.85E−03	lut
4	S3_99107971	A/T	2.09	1.06E−05	0.18	0.16	0.30	9.02E−03	lut
5	S4_8900031	C/T	2.33	4.87E−06	0.17	0.16	0.31	5.41E−03	lut
6	S4_229316518	G/A	−2.75	9.16E−06	0.08	0.16	0.30	8.25E−03	lut
7	S6_165089413	T/G	3.27	9.27E−06	0.03	0.16	0.30	8.29E−03	lut
8	S8_16743428	C/T	2.18	8.65E−09	0.21	0.16	0.41	6.34E−05	lut
9	S8_111289041	C/T	2.67	5.82E−09	0.16	0.16	0.42	5.34E−05	lut
10	S8_118971709	C/A	−2.80	1.49E−08	0.12	0.16	0.40	1.03E−04	lut
11	S8_121485958	C/T	2.36	3.60E−08	0.14	0.16	0.39	1.65E−04	lut
12	S8_123786605	T/C	−2.79	2.86E−08	0.11	0.16	0.39	1.65E−04	lut
13	S8_124434722	G/T	−0.43	3.97E−10	0.25	0.16	0.46	7.28E−06	lut
14	S8_128541902	C/T	2.53	4.19E−08	0.13	0.16	0.38	1.71E−04	lut
15	S8_130212000	A/G	−0.47	1.67E−08	0.16	0.16	0.40	1.08E−04	lut
16	S8_131682022	G/T	1.93	8.38E−08	0.25	0.16	0.37	3.29E−04	lut
17	S8_138510292	C/G	−0.51	2.87E−10	0.15	0.16	0.47	7.28E−06	lut
18	S8_138938949	C/T	−0.60	9.81E−12	0.13	0.16	0.53	5.39E−07	lut
19	S8_141803960	G/A	−3.04	4.23E−09	0.10	0.16	0.42	4.23E−05	lut
20	S8_144458630	A/G	2.81	6.93E−09	0.11	0.16	0.41	5.44E−05	lut
21	S9_112005623	C/G	2.16	4.43E−06	0.08	0.16	0.31	5.23E−03	lut
22	S10_136007575	G/A	−2.39	1.19E−09	0.22	0.42	0.62	6.55E−05	βcar
23	S10_139877594	G/A	−3.20	5.13E−08	0.13	0.42	0.57	1.13E−03	βcar
24	S10_116977608	G/C	−3.11	2.74E−07	0.15	0.42	0.55	3.11E−03	βcar
25	S10_136833624	C/T	2.59	4.98E−07	0.15	0.42	0.55	4.98E−03	βcar
26	S10_10289734	T/G	−1.96	1.07E−06	0.20	0.42	0.54	9.00E−03	βcar
27	S10_124427599	C/T	2.76	1.31E−06	0.18	0.42	0.54	9.00E−03	βcar
28	S10_74479633	G/C	−1.89	1.53E−06	0.19	0.42	0.53	9.89E−03	βcar
29	S10_134655704	T/C	−2.98	6.98E−08	0.13	0.22	0.42	3.28E−03	βcryp
30	S10_136840488	T/C	1.42	9.59E−08	0.18	0.22	0.41	3.28E−03	βcryp
31	S10_59877496	G/C	1.51	3.19E−07	0.16	0.22	0.39	4.96E−03	βcryp
32	S10_136833624	C/T	2.59	3.61E−07	0.15	0.22	0.39	4.96E−03	βcryp
33	S10_139877594	G/A	−3.20	6.51E−07	0.13	0.22	0.38	7.43E−03	βcryp
34	S8_137468530	C/T	−0.46	2.23E−07	0.23	0.16	0.36	9.60E−03	lnßbr/α-br
35	S8_138938949	C/T	−0.60	2.71E−07	0.13	0.16	0.35	9.60E−03	lnßbr/α-br
36	S8_117876676	T/C	0.44	4.37E−07	0.36	0.16	0.34	9.60E−03	lnßbr/α-br
37	S10_136007578	G/T	2.39	6.75E−10	0.22	0.38	0.60	3.15E−05	lnßcar/βcryp
38	S10_139877594	G/A	−3.20	9.60E−10	0.13	0.38	0.60	3.15E−05	lnßcar/βcryp
39	S10_136833624	C/T	2.59	2.69E−08	0.15	0.38	0.56	3.67E−04	lnßcar/βcryp
40	S10_116977608	G/C	−3.11	2.78E−08	0.15	0.38	0.55	3.67E−04	lnßcar/βcryp
41	S10_141574617	T/C	−2.03	9.58E−08	0.13	0.38	0.54	8.10E−04	lnßcar/βcryp
42	S10_4749679	G/C	−1.43	6.57E−07	0.38	0.38	0.52	3.44E−03	lnßcar/βcryp
43	S10_134650981	A/T	3.03	1.65E−09	0.13	0.30	0.53	1.04E−04	lnßcar/zea
44	S10_71671890	A/T	2.03	2.52E−07	0.15	0.30	0.46	4.61E−03	lnßcar/zea
45	S10_139877594	G/A	−3.20	3.90E−07	0.13	0.30	0.46	5.36E−03	lnßcar/zea
46	S2_44473758	C/T	−5.61	1.78E−07	0.21	0.16	0.36	9.26E−03	tcar
47	S2_139644276	G/A	−1.10	2.53E−07	0.35	0.16	0.36	9.26E−03	tcar
48	S10_134601800	G/A	−2.09	6.39E−07	0.29	0.38	0.51	2.68E−02	pva
49	S10_136007578	G/T	2.39	7.32E−07	0.22	0.38	0.51	2.68E−02	pva
50	S10_136840488	T/C	1.42	5.53E−07	0.18	0.15	0.33	4.76E−02	zea
51	S10_134655704	T/C	−2.98	8.84E−07	0.13	0.15	0.32	4.76E−02	zea
52	S10_135634185	G/A	−1.52	2.92E−06	0.31	0.15	0.31	4.76E−02	zea
53	S10_139075941	A/C	2.03	4.00E−06	0.15	0.15	0.30	4.76E−02	zea

a Representative significant SNPs selected based on their positions and approximate LD decay. Significant SNPs were selected at FDR 1%, except for chromosome 8 SNPs associated with lutein - which were selected only at Bonferroni 1%. For zeaxanthin and total provitamin A the threshold was set at 5% FDR to be able to detect significant SNPs. Some SNPs may appear two to four times as they were associated with multiple related traits. βcar, β-carotene; βcryp, β-cryptoxanthin; lut, lutein; zea, zeaxanthin; pva, provitamin A; tcar, total carotenoid; β br/αbr, ratio of carotenoids on β to α branch; Chr, Chromosome; MAF, minor allele frequency; FDR, false discovery rate. Allelic effects of SNPs indicated refer to the second allelic variants. The number after “S” before the underscore in each SNP’s name refers to the chromosome number; The number indicated after the underscore is the concerned SNP’s position.

**Table 5:** Candidate protein coding genes associated with carotenoid content and composition in maize endosperm detected by GWAS

SNP name	lowest *P*-value	Trait affected	*R*^2^ of Model	*R*^2^ of Model + SNP	Stable ID for the closest linked gene	Gene name	Gene description	strand	AGPv2 Gene start (bp)	AGPv2 Gene end (bp)
S10_136007575/S10_136007579	6.7E−10	lnbcar/bcryp	0.38	0.60	GRMZM2G152135	crtRB1*^a^*	Beta-carotene hydroxylase 1	−1	136,057,100	136,060,219
S8_138888278*^b^*	3.2E−08	lut	0.16	0.39	GRMZM2G012966	lcyE	Lycopene epsilon cyclase1	1	138,882,594	138,889,812
S2_44448432/S2_44448438	1.1E−06	tcar, zea*^c^*	0.16	0.33	GRMZM2G127139	ZEP1	Zeaxanthin epoxidase1	−1	44,440,299	44,449,237
S2_44473758/S2_44473801	1.8E−07	tcar	0.16	0.36	GRMZM2G062559	—	Uncharacterized protein		44,471,623	44,474,212
S5_78384689*^d^*	7.1E−07	bcar, PVA	0.58	0.68	GRMZM2G102845	—	Auxin response factor 20	−1	78,381,834	78,389,884
S8_16743428	8.7E−09	lut	0.16	0.41	GRMZM2G143211	—	Uncharacterized protein	1	16,741,652	16,746,323
S8_16444572/S8_16444587	3.5E−08	lut	0.16	0.39	GRMZM2G380414	—	Ultraviolet-B-repressible protein	−1	16,443,989	16,444,752
S8_111289041	4.0E−10	lut	0.16	0.42	GRMZM2G333079	—	Uncharacterized protein	−1	111,287,695	111,290,414
S8_124434722	5.8E−09	lut	0.16	0.46	GRMZM2G330693	—	Uncharacterized protein	1	124,434,479	124,435,152
S8_138938949/S8_138938983*^b^*	9.8E−12	lut	0.16	0.53	GRMZM2G463133	—	Putative HLH DNA-binding domain superfamily protein	−1	138,938,542	138,943,955
S10_136007575/S10_136007578	6.8E−10	lnbcar/bcryp	0.38	0.60	GRMZM2G397684	—	Putative RING zinc finger domain superfamily protein	1	136,006,849	136,007,871
S10_134650981	2.0E−08	bcar, lnbcar/zea	0.38	0.59	GRMZM2G018314	—	Uncharacterized protein	1	134,647,347	134,652,537
S10_139877594	5.1E−08	bcar, lnbcar/bcryp	0.38	0.60	GRMZM2G080516	ereb2	AP2-EREBP transcription factor	−1	139,875,910	139,877,865

a SNP linked to *crtRB1* does not reside within the gene.

b S8_138888278 is not the most significant SNP linked with *lcyE* but was located within the gene, whereas SNPs S8_138938949/S8_138938983 were the most significant but were located in another gene ∼50 kb downstream of lcyE.

c Association with zeaxanthin detected only when accounting for the allelic effects of *lcyE* and *crtRB1*.

d S5_78384689 detected in GWAS that considered effects of *lcyE* and *crtRB1* as covariates on the bases of their allele specific markers. Genes and associated information retrieved from maizeGDB.org and gramene.org. Physical positions of SNPs and coordinates of genes given according to B73 RefGen_2. The number after “S” before the underscore in each SNP’s name refers to the chromosome number; and the number after the underscore is the concerned SNP’s position. βar, β-carotene; βcryp, β-cryptoxanthin; lut, lutein; zea, zeaxanthin; pva, provitamin A; tcar, total carotenoid βbr/αbr, ratio of carotenoids on α to β branch.

Using the Bonferroni approach to adjust the family-wise type I error rate at α = 0.01, 13 SNPs on chromosome 10 were associated with carotenoids of the β branch and some of the derived ratio traits (β-carotene, β-cryptoxanthin, β-carotene to β-cryptoxanthin and/or β-carotene to zeaxanthin). The ratio of α to β branch carotenoid was significantly affected by 10 SNPs on chromosome 8 at FDR 5%, the most significant SNPs in the group accounting for 33 and 36% of the variations in the derived trait, respectively. These SNPs were also significantly associated with lutein.

Associations with zeaxanthin (12 SNPs) and provitamin A (3 SNPs) could only be detected when relaxing the significance cutoff threshold to 5% FDR. The variances explained by the model involving the most significant SNPs were 33% for zeaxanthin (SNP S10_136840488, *P* = 5.53E−07) and 51% for provitamin A (SNP S10_134601800, *P* = 6.39E−07). These SNPs were also associated with β-carotene and its derived ratio traits.

### Genes in LD with significant SNPs

The genomic locations of significant SNPs were investigated to identify what protein-coding genes the SNPs were located in or adjacent to, zooming in based on SNP data retrieved from online databases for maize genome (http://www.maizegdb.org/ and http://ensembl.gramene.org/Zea_mays/). The list of all annotated genes, including those encoding uncharacterized proteins, within circa 0.8 Mb of the most significant SNPs are presented in Table S4 in File S3. Here only those candidate genes the closest to the most significant SNPs listed in Table 5 are described.

**Figure 3:**
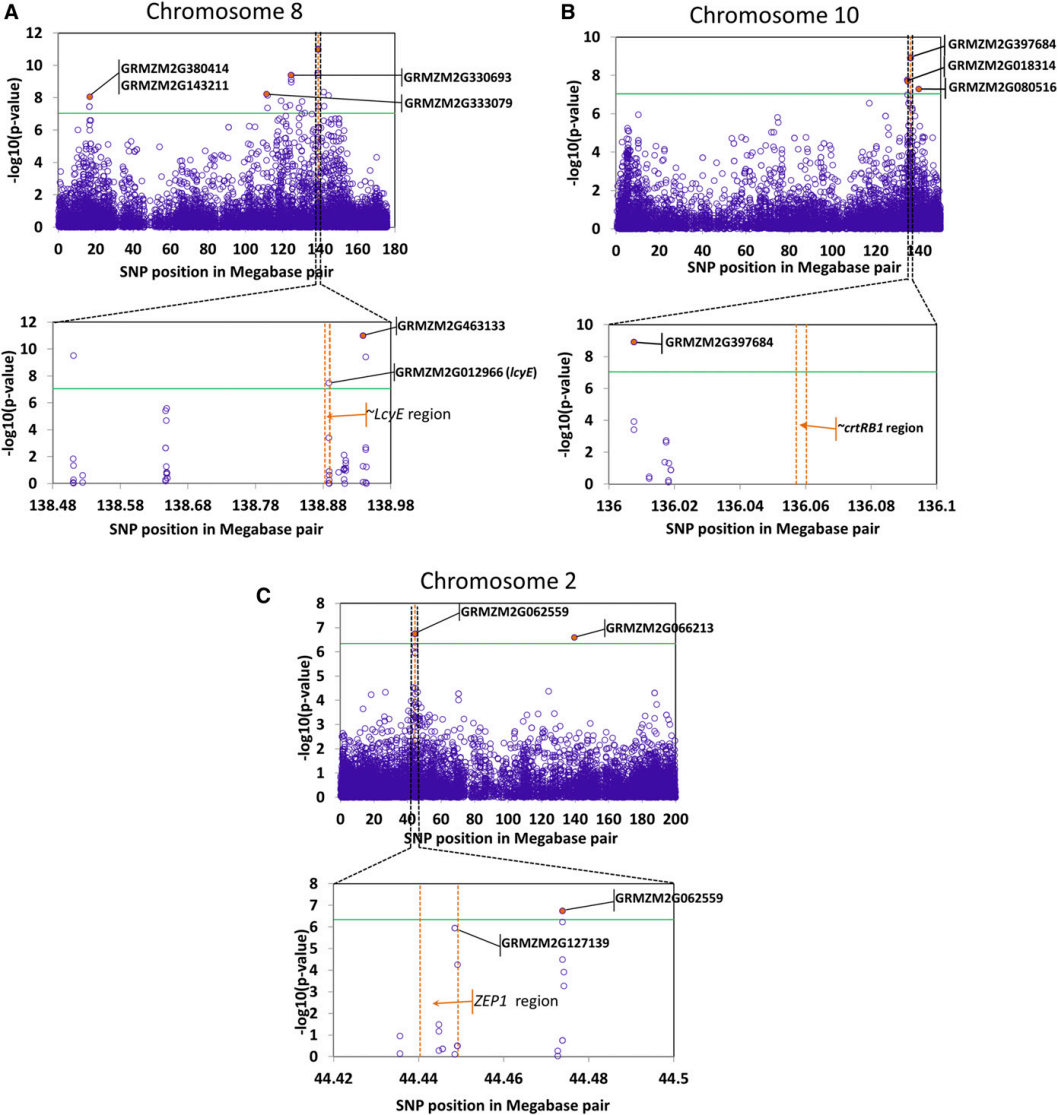
Scatter diagrams showing statistically significant association signals. (a) chromosome 8 for lutein, (b) chromosome 10 for β-carotene, (c) chromosome 2 for zeaxanthin. The most significant SNPs are highlighted in orange color and labeled with the IDs of the putative genes. Light green horizontal lines represent 1% Bonferroni-adjusted significance threshold (−log_10_(p) = 7.04) except for chromosome 2, which refers to 5% Bonferroni significance threshold (−log_10_(p) = 6.34). Vertical orange lines show regions of the major carotenoid candidate genes *lcyE*, *crtRB1*, and *ZEP1*. Plots were made with Microsoft Excel.

**Figure 4:**
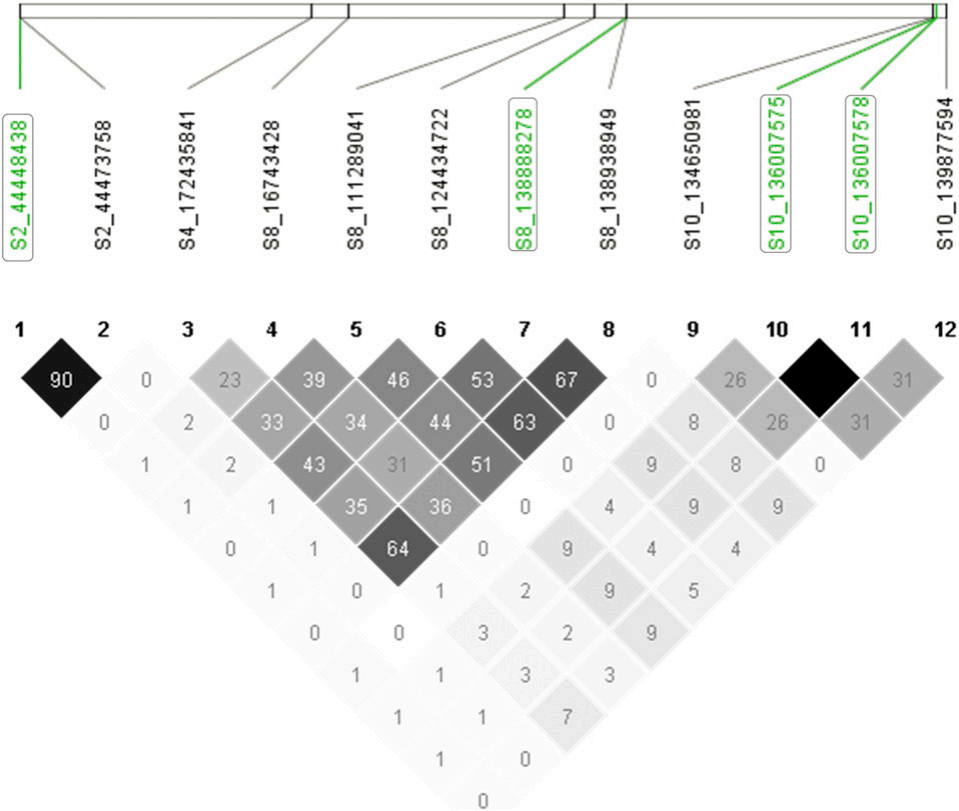
LD among selected peak SNPs. Numbers in cells are *R*^2^ values multiplied by 100, where the darkest gray scale shading denotes 100 and white denotes zero. The closest SNPs to the genes *ZEP1*, *lcyE*, and *crtRB1* (2 SNPs), appearing in the same left to right order, are highlighted in green and enclosed with boxes.

The most significant SNP in the association signal for lutein content ∼16 Mbp on chromosome 8 (SNP S8_16743428, *P*-value = 8.65E−09) was located within a putative gene GRMZM2G143211 (Figure 3a). This gene model contains a WD domain and displays sequence similarity to the yeast autophagy 18 (*AtATG18*) gene class in *Arabidopsis thaliana*. Two additional significant SNPs (S8_16444572 and S8_16444587) in this region were located within another candidate gene GRMZM2G380414, which encodes a protein called Ultraviolet-B-repressible which is likely involved in photosynthesis. The association peak between 110 and 144 Mbp on the same chromosome for the same trait contained three highly significant SNPs, namely S8_111289041 (*P*-value = 5.82E−09); S8_124434722 (*P*-value = 3.97E−10), and S8_138938949/S8_138938983 (*P*-value = 9.81E−12) that were in strong LD to one another, with *R*^2^ value ranging from 0.31 to 0.67 (Figure 4). These SNPs were located within three different protein-coding putative genes GRMZM2G333079, GRMZM2G330693 and GRMZM2G463133, respectively with the first and third genes having some evidence of expression in maize endosperm (Sekhon *et al.* 2011). The two SNPs, S8_138938949 and S8_138938983, are 50 kb distal from one of the major carotenoid biosynthesis genes, *lcyE*. Pairwise LD among these highly significant SNPs on chromosome 8 varied from *R*^2^ = 0.23 to 0.67 (Figure 4).

On chromosome 10, the strong association peak surrounding the 138 Mbp region for β-carotene (Figure 2 and Figure 3b) contained two closely spaced and significant SNPs S10_136007575 and S10_136007578, *P* = 6.75E−10. These SNPs are the closest significant SNPs to the major candidate gene of carotenoid *crtRB1* (∼40 kb distal), but are physically located within a putative RING zinc finger domain protein coding gene, GRMZM2G397684 (Figure 3b). The other significant SNPs in this region were S10_134650981 (*P*-value = 1.99E−08) and S10_139877594 (*P*-value = 5.12E−08), residing within candidate genes GRMZM2G018314 and GRMZM2G080516, respectively. The latter encodes an AP2-EREBP transcription factor which is expressed in maize seed endosperm (Sekhon *et al.* 2011). LD among the peak SNPs on chromosome 10 ranged from 0 between SNPs S10_134650981 and S10_139877594, to 1, between SNPs S10_136007575 and S10_136007578 (Figure 4).

A small but significant SNP association was detected on the short arm of chromosome 2 coinciding with a gene involved in the conversion of carotenoids to abscisic acid, namely zeaxanthin epoxidase 1 (*ZEP1*, GRMZM2G127139). Two of the six SNPs that were significantly associated with total carotenoids at FDR 5% (S2_44448438, *P* = 1.11E−06) and S2_44448432 *P* = 1.11E−06) were physically located within the *ZEP1* gene (Figure 3c). However, the most significant SNP, S2_44473758, *P* = 1.78E−07, was located circa 33 kb downstream of the *ZEP1* gene within another protein-coding gene GRMZM2G062559 that encodes an uncharacterized protein. All of these SNPs were in high LD forming a haplotype block (Figure 4) when considered without the non-significant SNPs in the region. We consider that the significant effect most likely arises from *ZEP1* (or some of these SNPs could be linked with regulatory regions or control elements). The other SNP on the same chromosome around position 139 Mbp that showed a strong association with total carotenoid (S2_139644276, *P*-value = 2.53E−07) was located in a putative gene GRMZM2G066213 (Figure 3c).

**Figure 5:**
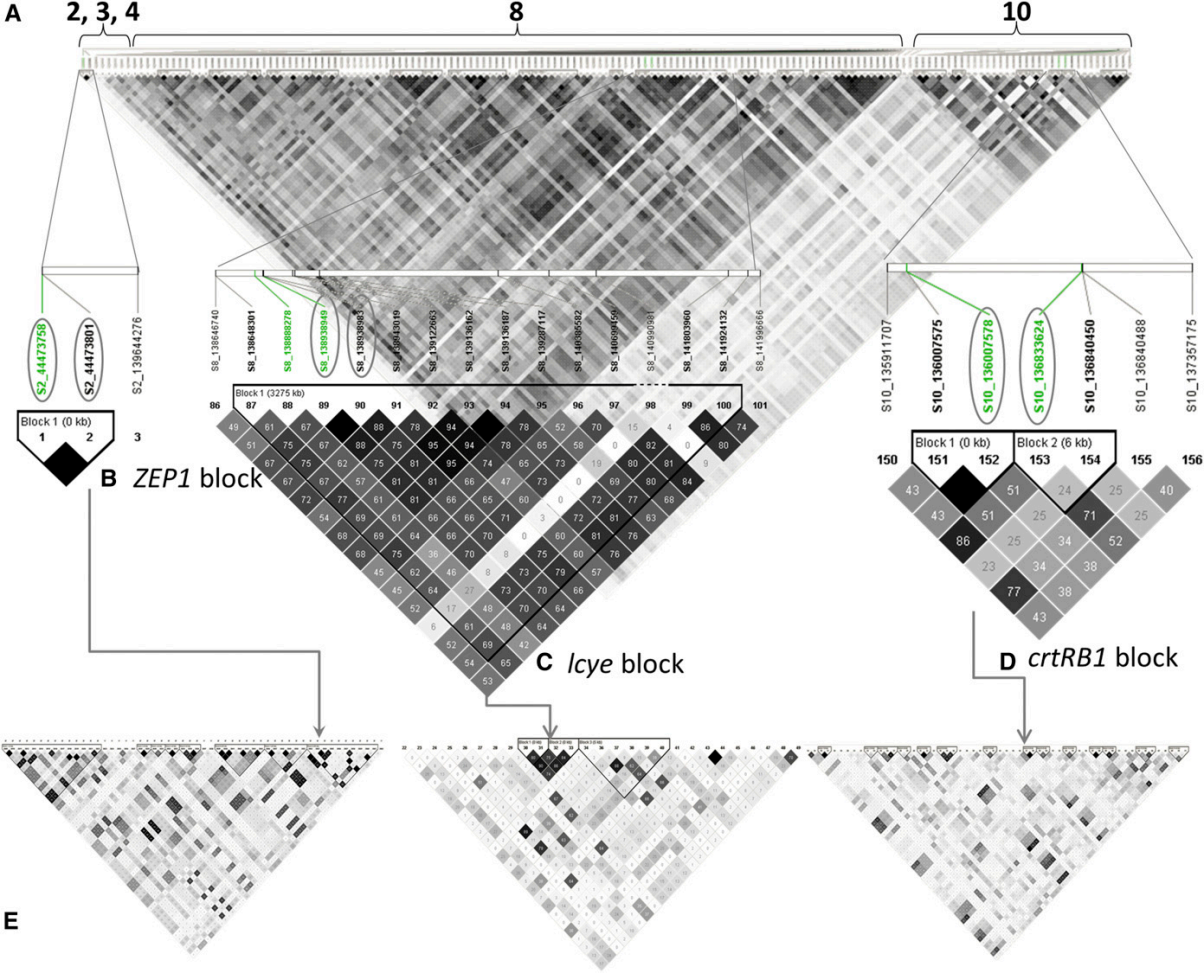
LD plots of significant SNPs and LD blocks surrounding the genes *lcyE*, *crtRB1* and *ZEP1*. (a) LD plot of all significant SNPs selected at FDR 1%. Labels 2, 3, 4, 8 and 10 refer to the chromosomes of the SNPs that reached significance at this threshold; (b) an LD block on chromosome 2 surrounding the gene *ZEP1*; (c) an LD block on chromosome 8 surrounding the gene *lcyE*; (d) an LD block on chromosome 10 comprising the gene *crtRB1*; (e) LD plots that included non-significant SNPs in regions +/− of *crtRB1*, *lcyE* and *ZEP1* where significant associations were detected. Haplotype blocks were defined with the option of confidence interval (Gabriel *et al.* 2002). Green highlighted SNPs are the closest SNPs to the carotenoid genes indicated, with the most significant ones enclosed with oval shapes. The grayscale shading pattern of LD plot reflects the strength of linkage as it increases from the lightest to the darkest shaded cells paralleling the range of no LD (*R*^2^ = 0%) to absolute LD (*R*^2^ = 100%). Plots generated using HaploView software (Barrett *et al.* 2005).

Strong and extensive pairwise LD was observed among the significant SNPs selected at 1% FDR (Figure 5a and Table 6). Seventy percent of the pairwise comparison among the SNPs led to statistically significant LD (*P <* 0.001) of which 21% was comprised of inter-chromosomal correlations. LD for within chromosome comparisons ranged from 0.37 to 1, both on chromosome 3, with genome-wide average of 0.42. For inter-chromosomal comparisons, LD ranged from 0.18 in chromosome 10 to 0.5 in chromosome 3, with a genome-wide average of 0.25. Significant SNPs on chromosomes 3 and 4 displayed strong inter-chromosomal LD with those on chromosome 8, but was negligible for SNPs on chromosome 10. The confidence interval algorithm deployed in HaploView software generated 11 haplotype blocks based on LD of the significant SNPs on chromosome 8, five blocks for those on chromosome 10 and one block for those on chromosome 2. Haplotype blocks were identified for each of the three carotenoid genes *crtRB1*, *lcyE* and *ZEP1* when analyzing the significant SNPs in regions surrounding their corresponding genomic locations. Further analysis of LD for regions comprising these three genes, with the inclusion of non-significant SNPs revealed heterogeneous LD. This suggests that the LD among the significant SNPs residing in regions of these major genes could be functional, rather than tight genetic linkage occurring as a result of long range average LD decay in the association panel.

**Table 6:** Summary of LD analysis among significant SNPs at 1% FDR

		Chr 2	Chr 3	Chr 4	Chr 8	Chr 10	Total
No. of SNPs		3	3	2	119	36	163
% Significant LD (*P* < 0.001)		33	67	100	96	86	70
Haplotype blocks		1	—	—	11	5	17
Intra-chromosome LD	% *P* < 0.001						79
	minimum	1.00	0.34	0.59	0.09	0.13	0.09
	maximum	1.00	0.40	0.59	1.00	1.00	1.00
	average	1.00	0.37	0.59	0.42	0.45	0.42
Inter-chromosome LD	% *P <* 0.001						20.70
	minimum	0.10	0.13	0.15	0.10	0.10	0.10
	maximum	0.23	1.00	0.75	0.77	0.54	1.00
	average	0.15	0.40	0.37	0.28	0.18	0.25

Chr, chromosome; % *P <* 0.001, percentage of significant LD (*r*^2^) having their *P*-values <0.001.

### GWA re-calculated with the allele specific markers of crtRB1 and lcyE included as covariates

**Figure 6:**
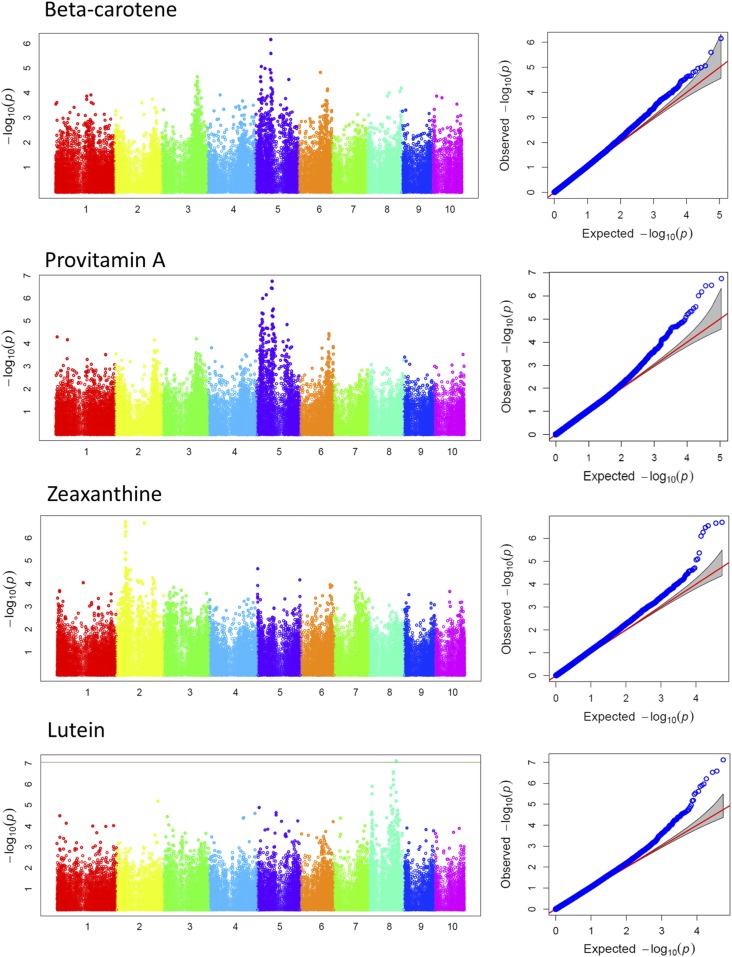
Manhattan and QQ-plots for GWAS conducted with allele specific markers of *crtRB1* and *lcyE* genes as covariates. The association signals were significant after 5% FDR correction of the *P*-values.

GWA was re-calculated by incorporating the allele specific markers of the two genes *lcyE* and *crtRB1* as additional fixed effect covariates in the MLM model. As expected, the number of SNPs significantly associated with the traits in this analysis was drastically reduced from 386 in the previous analysis to 38 SNPs (excluding the four SNPs significant at 10% FDR), at a cut-off threshold of 5% FDR (Table 7). Numerous SNPs on chromosome 8 and 10 previously associated with lutein and β-carotene (plus its derived traits) became statistically non-significant, even at a lower significance threshold of 10% FDR (Figure 6 and Table 7). Using this approach, chromosome 10 was devoid of significant SNPs, and only two SNPs on chromosome 8 (SNP S8_138938949 and S8_138938983) were strongly associated with lutein (*P*-value = 7.66E−08; *R*^2^ = 0.53). These SNPs were also the most significant SNPs in the initial GWAS result. The SNPs were physically located within a putative gene GRMZM2G463133 encoding an HLH binding domain protein. Since these two SNPs were in high LD with SNPs in the *lcyE* region (Figure 5c), it is possible that the significant effect arises from linkage to this known carotenoid biosynthesis gene. However, functional studies would be required to unequivocally ascribe the significant effect to these SNPs.

**Table 7:** Summary of significant SNPs identified by GWAS with allele specific markers of *lcyE* and *crtRB1* included as covariates

No.	SNP	Allele	Allelelic effect	*P*-value	MAF	*R*^2^ of Model	*R*^2^ of Model + SNP	FDR adjusted *P*-value	Carotenoid
1	S2_43342654	G/A	3.43	8.21E−07	0.33	0.33	0.48	0.011278	zea
2	S2_44448432	C/T	−3.99	2.89E−07	0.20	0.33	0.49	0.006272	zea
3	S2_44448438	T/G	3.99	2.89E−07	0.20	0.33	0.49	0.006272	zea
4	S2_44473748	T/G	3.86	3.42E−07	0.21	0.33	0.49	0.006272	zea
5	S2_44473758	C/T	−5.01	2.03E−07	0.21	0.33	0.49	0.006272	zea
6	S2_44473801	G/A	5.01	2.03E−07	0.21	0.33	0.49	0.006272	zea
7	S2_45967604	T/G	−0.44	5.55E−07	0.37	0.33	0.48	0.008711	zea
8	S2_139644276	G/A	−3.85	2.27E−07	0.35	0.33	0.49	0.006272	zea
9	S2_36077381	G/A	−0.97	8.27E−07	0.05	0.67	0.74	0.013309	lnßcar/zea
10	S2_43376157	C/T	0.95	3.40E−07	0.06	0.67	0.74	0.010586	lnßcar/zea
11	S2_44474088	G/A	−0.95	8.47E−07	0.05	0.67	0.74	0.013309	lnßcar/zea
12	S2_45967604	T/G	−0.44	2.20E−06	0.37	0.67	0.73	0.026892	lnßcar/zea
13	S2_47044902	A/C	0.82	1.85E−06	0.07	0.67	0.73	0.02546	lnßcar/zea
14	S2_47310378	C/G	0.93	3.85E−07	0.06	0.67	0.74	0.010586	lnßcar/zea
15	S2_47310382	G/C	−0.93	3.85E−07	0.06	0.67	0.74	0.010586	lnßcar/zea
16	S2_103681279	T/C	−0.76	7.17E−07	0.09	0.67	0.74	0.013309	lnßcar/zea
17	S2_109770228	A/C	0.90	3.75E−07	0.07	0.67	0.74	0.010586	lnßcar/zea
18	S7_108535010	A/G	0.62	3.55E−06	0.10	0.67	0.73	0.03903	lnßcar/zea
19	S2_208672678	C/G	2.67	6.67E−06	0.03	0.37	0.48	0.048918	lut
20	S8_16444572	T/C	−1.47	2.83E−06	0.29	0.37	0.49	0.025904	lut
21	S8_16444587	T/A	−1.47	2.83E−06	0.29	0.37	0.49	0.025904	lut
22	S8_16743428	C/T	1.69	1.25E−06	0.21	0.37	0.50	0.017159	lut
23	S8_111289041	C/T	2.00	3.35E−06	0.16	0.37	0.49	0.028289	lut
24	S8_111803908	A/G	1.90	1.45E−06	0.18	0.37	0.50	0.017674	lut
25	S8_124434722	G/T	1.84	6.13E−07	0.25	0.37	0.51	0.011226	lut
26	S8_124434723	C/G	1.79	1.08E−06	0.25	0.37	0.50	0.016903	lut
27	S8_124434725	G/A	−1.74	2.59E−07	0.28	0.37	0.52	0.008199	lut
28	S8_124434726	C/G	1.69	2.98E−07	0.29	0.37	0.52	0.008199	lut
29	S8_124434730	G/C	−1.84	6.13E−07	0.25	0.37	0.51	0.011226	lut
30	S8_138510292	C/G	2.28	2.38E−06	0.15	0.37	0.49	0.025904	lut
31	S8_138938949	C/T	2.82	7.66E−08	0.13	0.37	0.53	0.004208	lut
32	S8_138938983	C/T	2.82	7.66E−08	0.13	0.37	0.53	0.004208	lut
33	S8_138943019	C/T	2.49	6.36E−06	0.12	0.37	0.48	0.048918	lut
34	S2_44473758	C/T	−5.01	2.06E−06	0.21	0.20	0.35	0.075561	tcar
35	S2_44473801	G/A	5.01	2.06E−06	0.21	0.20	0.35	0.075561	tcar
36	S2_139644276	G/A	−3.85	9.62E−07	0.35	0.20	0.36	0.075561	tcar
37	S5_78384689	C/T	1.14	7.08E−07	0.27	0.76	0.81	0.077827	βcar
38	S5_30601081	C/G	1.45	1.03E−06	0.33	0.58	0.67	0.022747	tpva
39	S5_48678892	G/A	−1.56	6.97E−07	0.37	0.58	0.67	0.01915	tpva
40	S5_74462863	C/T	2.05	3.55E−07	0.20	0.58	0.67	0.013758	tpva
41	S5_78384689	C/T	1.14	1.81E−07	0.27	0.58	0.68	0.013758	tpva
42	S5_78427240	G/T	1.53	3.75E−07	0.27	0.58	0.67	0.013758	tpva

βcar, β-carotene; βcryp, β-cryptoxanthin; lut, lutein; zea, zeaxanthin; pva, provitamin A; tcar, total carotenoid; Chr, Chromosome; MAF, minor allele frequency; FDR, false discovery rate. Effects indicated are for the second allelic variants of SNPs. The number after “S” before the underscore in each SNP’s name refers to the chromosome number; The number indicated after the underscore is the concerned SNP’s position.

On the other hand, the re-run GWAS detected new significant associations on chromosome 5 for β-carotene and provitamin A. In particular, SNPs S5_78384689 and S5_78427240 were associated with provitamin A at 5% FDR (*P*-value = 1.81E−07; *R*^2^ = 68) and one of these SNPs S5_78384689, was associated with β-carotene at 10% FDR (*P*-value= 7.08E−07; *R*^2^ 0 = 81). The SNP S5_78384689 lay within an auxin-response factor 20 gene (GRMZM2G102845, 5:78,381,834–78,389,884, Table 5). In addition, seven SNPs on chromosome 2 were significantly associated with zeaxanthin content at FDR 1% (Table 7). Three of the zeaxanthin associated SNPs (44,473,748, S2_44473758, and S2_44473801) were located within the gene *ZEP1* while the other two were located 23 kb upstream of the *ZEP1* gene.

## Discussion

Genome-wide and candidate-gene based association studies are powerful approaches to identify nucleotide variants that functionally underlie important agronomic and nutritional traits. Such nucleotide variants can be harnessed in breeding programs to develop improved cultivars through marker- or genomics-assisted selection (Babu *et al.* 2013; Hamblin *et al.* 2011). Association mapping using large population sizes and high marker densities can be used for successful and reliable prediction of LD and associations between alleles and target phenotypes (Yu and Buckler 2006; Khatkar *et al.* 2008; Zhu *et al.* 2008).

As a major staple crop, maize has been the focus of both candidate gene and GWA studies for a number of agriculturally and nutritionally significant phenotypes (Cook *et al.* 2012; Li *et al.* 2012, 2013; Lipka *et al.* 2013; Yan *et al.* 2010; Yu and Buckler 2006). In our GWA study, we have used a panel of 130 diverse and partially related inbred lines of maize where we have used genome-wide GBS to generate a highly-dense SNP map for association analyses. Our use of inbred lines combining the genomes of both temperate and tropical maize germplasm has allowed us to capture small to large effect carotenoid allelic variants that are present in the two gene pools within IITA’s maize breeding program.

Despite its predisposition to large levels of missing data (Heslot *et al.* 2013), GBS generates large number of SNPs with dense coverage and potentially less ascertainment bias, which is ideal for consistent GWAS (Crossa *et al.* 2013; Elshire *et al.* 2011). The SNP data set used in our GWA study had acceptable level of missing data of only 10% (which was predicted with conservative imputation criteria in GAPIT genetic analysis software to allow reliable genome-wide associations). A minimum MAF criteria of 1% was used to filter out potential spurious SNPs stemming from sequencing error (Glaubitz *et al.* 2014).

The frequency of minor alleles is an important factor that can affect the accuracy of LD analysis and GWAS especially when using small samples (Tabangin *et al.* 2009; Yan *et al.* 2009). The filtered data set had a large proportion of MAFs distributed uniformly across the genome, frequencies ranging between 1 and 5% accounting for the largest proportion. However, the MAFs of the vast majority of the significant SNPs were above 10% which might be indicative of the positive detection power of the GWAS as the biasness associated with rare alleles when using small sized samples for association mapping was eliminated (Schnable *et al.* 2009). This could suggest that alleles associated with carotenoid content and composition may be segregating in our panel at frequencies higher than 10% (Hamblin *et al.* 2011).

The average genome-wide LD decay in our study was estimated at circa. 830 kbp at a background critical *R*^2^ = 0.2. Previous studies in maize reported LD extent to be <1000 bp for maize landraces, >2000 bp for diverse breeding lines, and ∼100 kb for commercial elite inbred lines (Yu and Buckler 2006). Although it can lack the power for high precision mapping, a mapping panel with persistent LD can be considered ideal, if low-resolution mapping is targeted (Flint-Garcia *et al.* 2003). The long range LD in this panel was expected since such extensive LD is characteristics of advanced maize inbred lines that have experienced strong recent selection (Yu and Buckler 2006). Also, small populations are prone to genetic drift leading to loss of rare alleles and increased LD (Flint-Garcia *et al.* 2003). Nonetheless, there was considerable localized variation in LD structure across the genome suggesting that the mapping resolution also vary. The extensive LD in our study could lead to the identification of SNPs in genes that are either causal or contributory to the carotenoid phenotype, or which act as linked markers associated with the carotenoid phenotype.

The two MLM GWAS models we employed detected a number of small to large effect known carotenoid biosynthesis genes, as well as several putative genes encoding characterized or uncharacterized proteins. The first MLM GWAS considered population structure (Q) and relative kinship (K) only, while the second incorporated the allele specific markers of *lcyE* and *crtRB1* major carotenoid genes as additional fixed effect covariates. The identification of known carotenoid biosynthesis genes in our study indicates that our study had sufficient power to identify causal or contributory genes.

The vast majority of highly significant hits in the first GWAS were on chromosome 8 associated with lutein, followed by chromosome 10 associated with β-carotene and the ratio of β-carotene to β-cryptoxanthin. These significant SNPs were in chromosomal regions where the genes *lcyE* and *crtRB1* were located on chromosome 8: 138,882,594–138,889,812 and chromosome 10:136,057,100–136,060,219, respectively (Figure 3, a and b, Table 4, and Table 5). The large effects of these genes on carotenoids within the maize panel used in our study were expected, as the markers designed to detect the allelic variants of these genes were previously confirmed (Harjes *et al.* 2008; Yan *et al.* 2010) to have significant impact in the same mapping panel (Azmach *et al.* 2013), indicating the successful introgression of the favorable alleles of these two genes into the tropical yellow maize genetic background.

While the most significant SNP (*P* = 9.81E−12; Table 4 and Table 5) on chromosome 8 was located 49 kb downstream of the *lcyE* gene, another significant SNP (SNP: S8_138888278, *P* = 3.19E−8) was detected within the gene ∼1 kb upstream of the 3′indel functional polymorphism that was previously reported by Harjes *et al.* (2008). Despite no SNP was found within the gene *crtRB1*, the closest SNPs (S10_136007575 and S10_136007578) significantly associated with β-carotene to β-cryptoxanthin ratio (*P*-value = 6.75E−10) and other ratio involving β-carotene were located 50 kb upstream of this gene.

The inclusion of the allele-specific marker information for *lcyE* and *crtRB1* as additional fixed effect covariate allowed to control for the large effects of the two genes. Using this approach, 10% of the significant SNPs detected at 5% FDR in the first GWAS survived the correction for the allele specific markers. The two most significant SNPs on chromosome 8 detected in the first GWAS still displayed a strong association with lutein levels, which could be due to a stronger LD of the SNPs with larger-effect functional polymorphisms in the 3′TE untranslated region of *lcyE* not captured with the present genotyping and possibly different from the polymorphisms previously described by Harjes *et al.* (2008). This could explain the relatively low effect of the known allele-specific markers of *lcyE* in our previous marker validation study (Azmach *et al.* 2013), although the strongest association was still detected in this gene region in our GWA study.

The controlling of the effects of *lcyE* and *crtRB1* in the GWAS including marker covariates led to the detection of significant associations for zeaxanthin levels on chromosome 2 at 1% FDR. The significant SNPs co-localized with a known downstream carotenoid biosynthesis gene *ZEP1* (chromosome 2: 44,440,299–44,449,237). These SNPs were detected in the first MLM GWAS, but they were then significantly associated with total carotenoid content at a 5% FDR. In the GWAS without covariates, zeaxanthin was significantly affected by SNPs only from the large association signal detected on chromosome 10. This could suggest that increases or decreases in the rate of conversion of β-carotene to zeaxanthin through β-cryptoxanthin may be more pronounced than that of decreases in the rate of conversion of zeaxanthin to violaxanthin by *ZEP1* in the maize inbred line panel used in our study. This would provide a reason for the greater impact of *crtRB1* on the level of zeaxanthin than *ZEP 1*. This could be interpreted as a scenario where reduced function of *crtRB1* leads to accumulation of β-carotene at the expense of zeaxanthin synthesis, reflecting the larger effect of *crtRB1* on the concentration of zeaxanthin.

Indeed, recent association studies have reported similar associations of SNPs within the gene *ZEP1* with zeaxanthin content (Owens *et al.* 2014; Suwarno 2012). In addition a small effect QTL underlying kernel color close to the gene *ZEP1* has also been reported and suggested as a target for allele mining by Chandler *et al.* (2013). This locus can therefore be considered as one of the loci potentially contributing to the variation in total carotenoid in the mapping panel used in this study and can be the next target gene for allele mining. Allele-combinations of *ZEP1* and other genes in the biosynthesis pathway can be used in breeding programs to increase accumulation of provitamin A and total carotenoid in maize endosperm.

Our GWAS including marker covariates also identified an association between SNPs on chromosome 5 and provitamin A at 5% FDR and β-carotene at 10% FDR. These SNPs were co-localized with a gene encoding an auxin-response factor 20 (arf20) protein (5:78,381,834–78,389,884). Auxin-response factors are transcription factors that target auxin response DNA elements (AuxRE) in the promoters of auxin-regulated genes (Li *et al.* 2016). The key enzymes involved in carotenoid biosynthesis in cereals are well known and have been previously reviewed; *e.g.*, figure 1 in Zhai *et al.* (2016). This family of transcription factors is known to have a role in conditioning carotenoid biosynthesis through coordinated regulation of transcription of genes involved in the pathway (Meier *et al.* 2011). As the *ARF20* gene is highly expressed in the maize endosperm (Sekhon *et al.* 2011), the gene may constitute a novel target for further unraveling of the regulatory mechanism of carotenoid biosynthesis in maize endosperm.

### Conclusions

Using a panel of IITA’s tropical maize inbred lines that incorporated high β-carotene alleles introgressed from a temperate maize germplasm, our study detected SNPs co-localizing with known major and small effect carotenoid biosynthesis genes, demonstrating the detection power of our GWA analyses. In addition, a number of associations were detected for novel candidate genes encoding transcription factors, which might have roles in regulation of the carotenoid biosynthesis in maize endosperm. As our study is based on IITA’s tropical maize breeding program, it can contribute to transitioning of the maize biofortification efforts of the breeding program toward molecular-marker assisted approaches. Our findings pave the way for additional allele mining efforts and greater understanding of the genes involved in regulation of expression of carotenoid biosynthesis genes, which is necessary to further exploit the genetic potential of maize in accumulating provitamin A in maize endosperm.

## Supplementary Material

Supplemental material is available online at www.g3journal.org/lookup/suppl/doi:10.1534/g3.117.300511/-/DC1.
