# Supplementary figures and images for: Genetic Loci Controlling Carotenoid Biosynthesis in Diverse Tropical Maize Lines
Source: G3 (Bethesda). 2018 Jan 29;8(3):1049–65. doi: 10.1534/g3.117.300511 (PMC5844293; doi:10.1534/g3.117.300511)

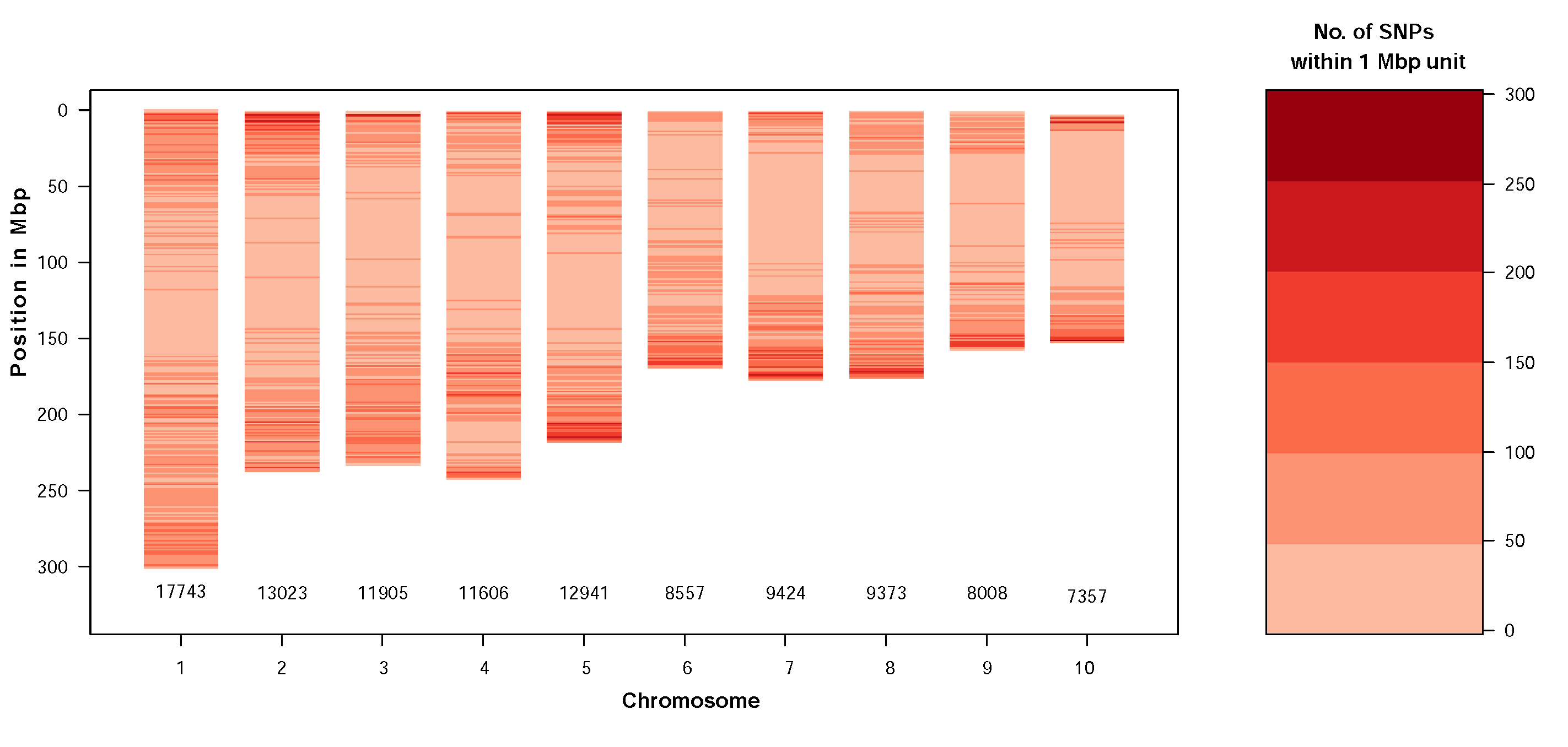

Supplement: Supplementary file 1 [file 1049FigureS1.tif]

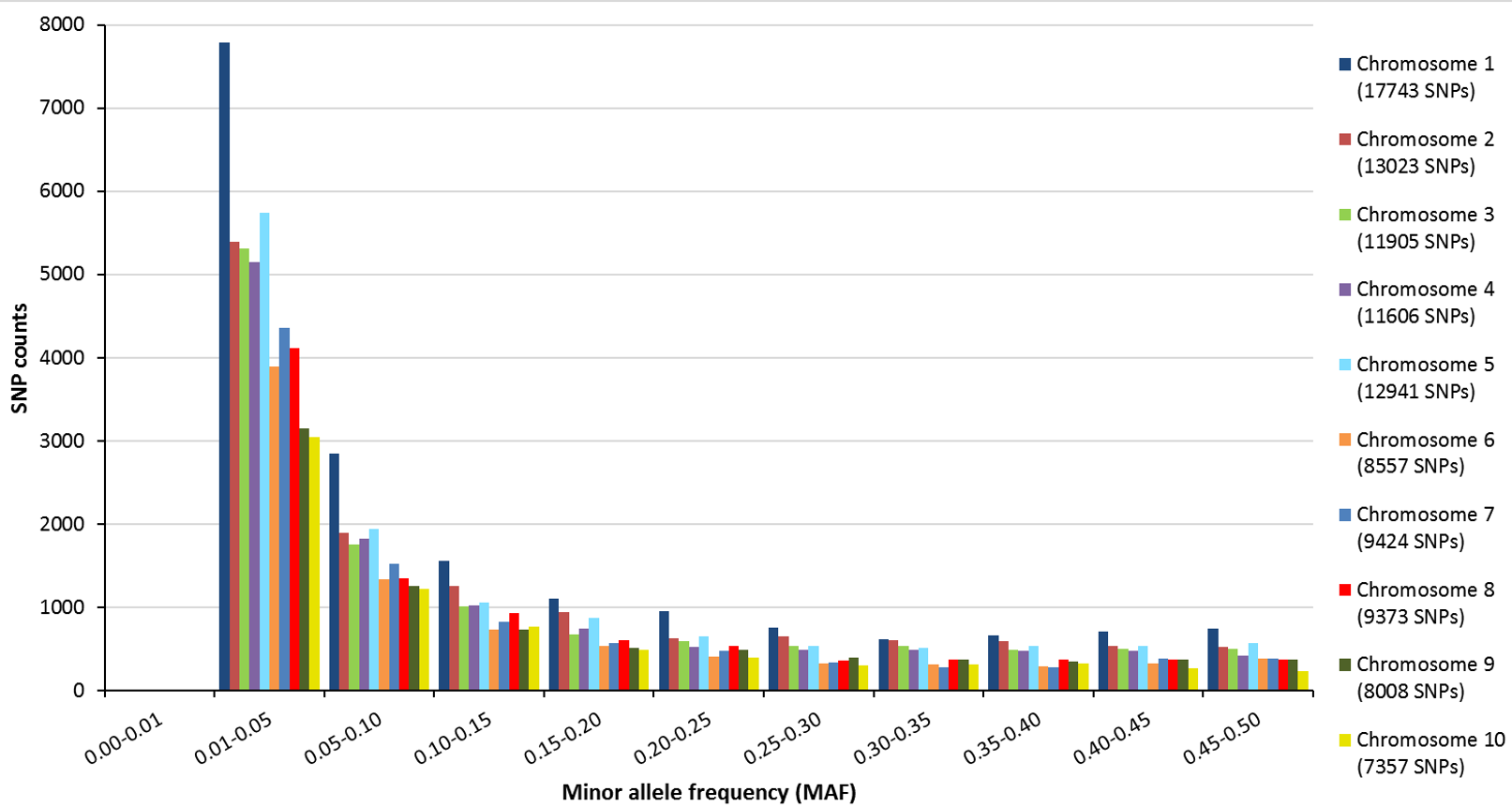

Supplement: Supplementary file 2 [file 1049FigureS2.tif]

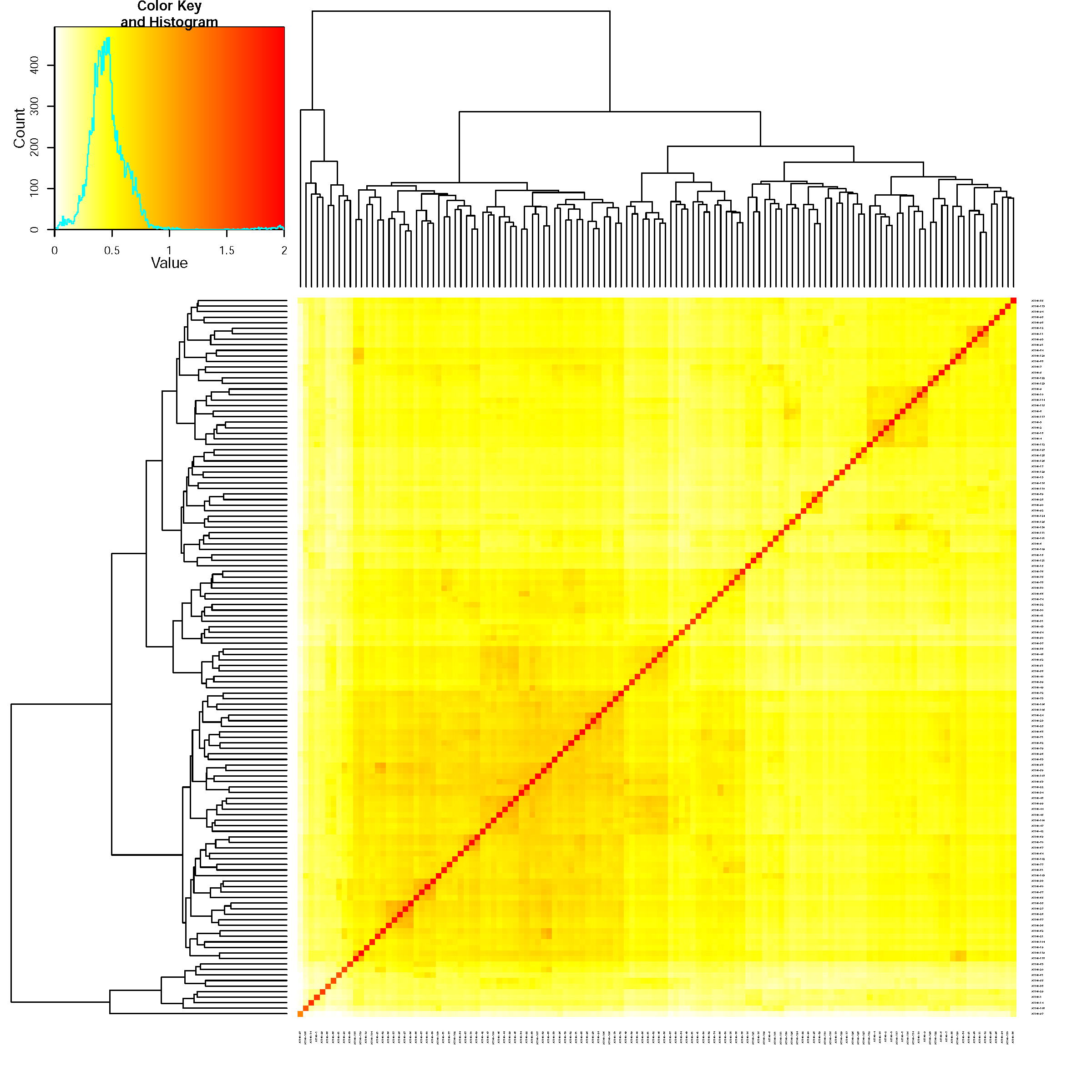

Supplement: Supplementary file 3 [file 1049FigureS3.tif]

## Slide 1
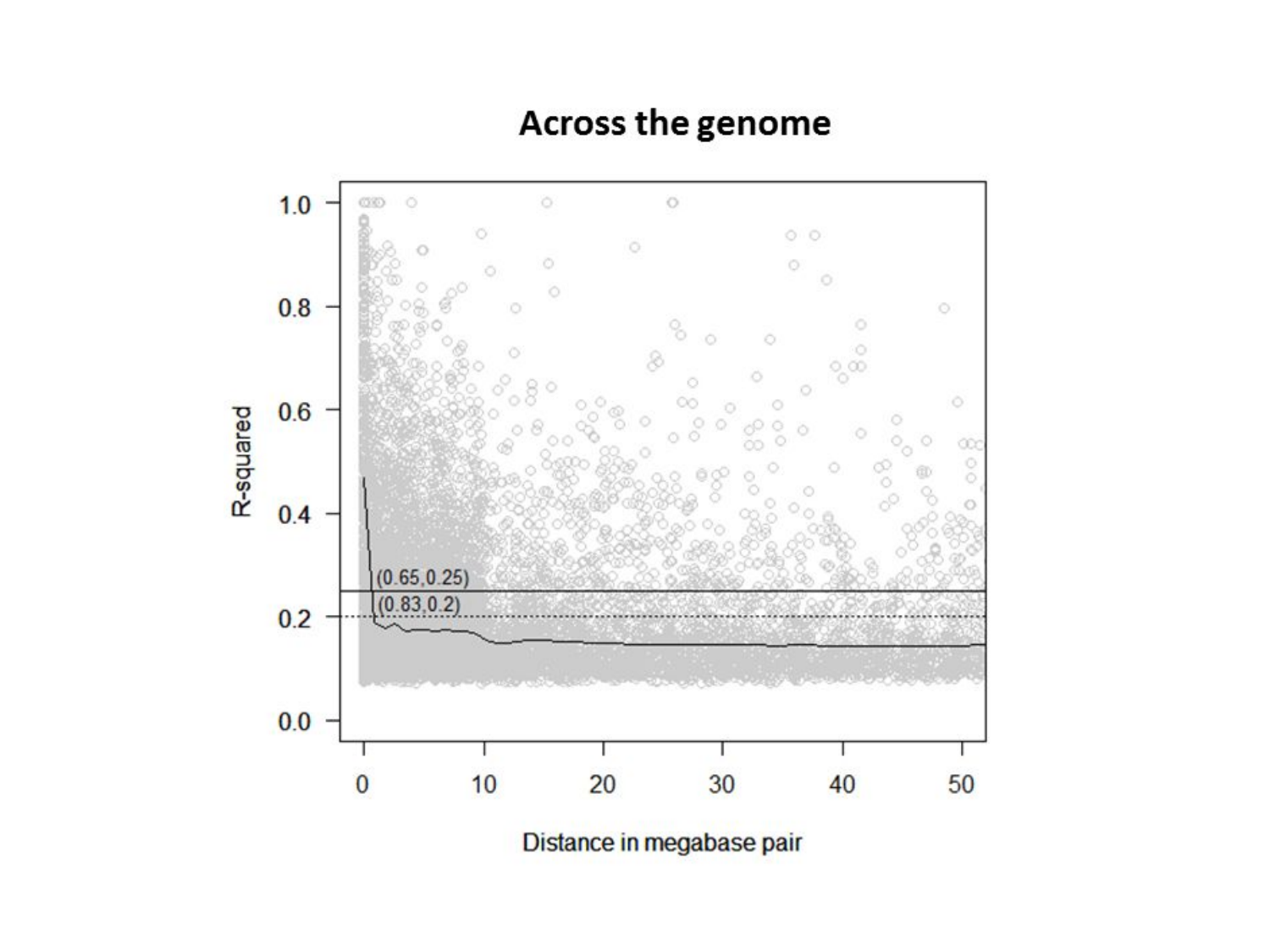

## Slide 2
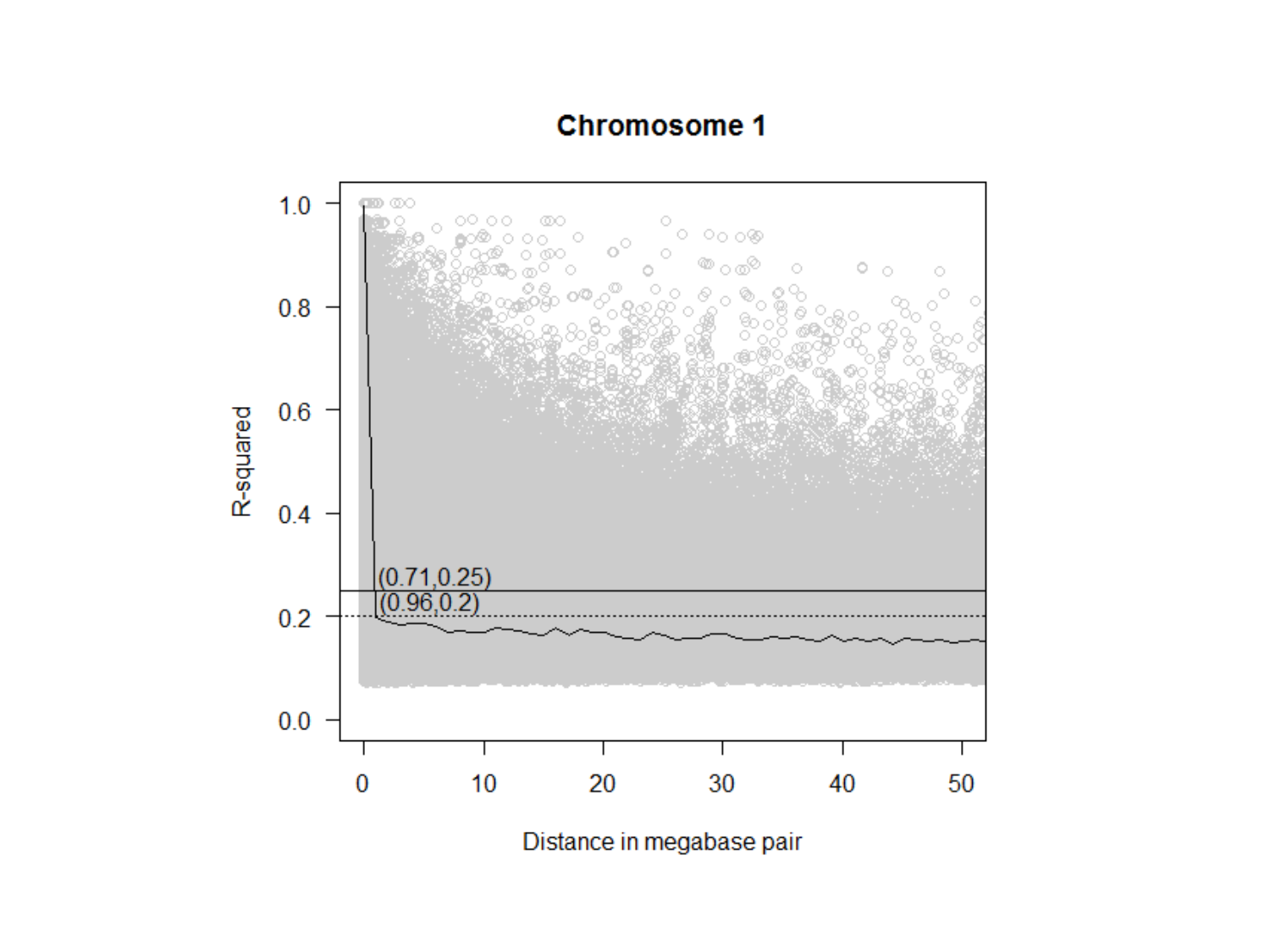

## Slide 3
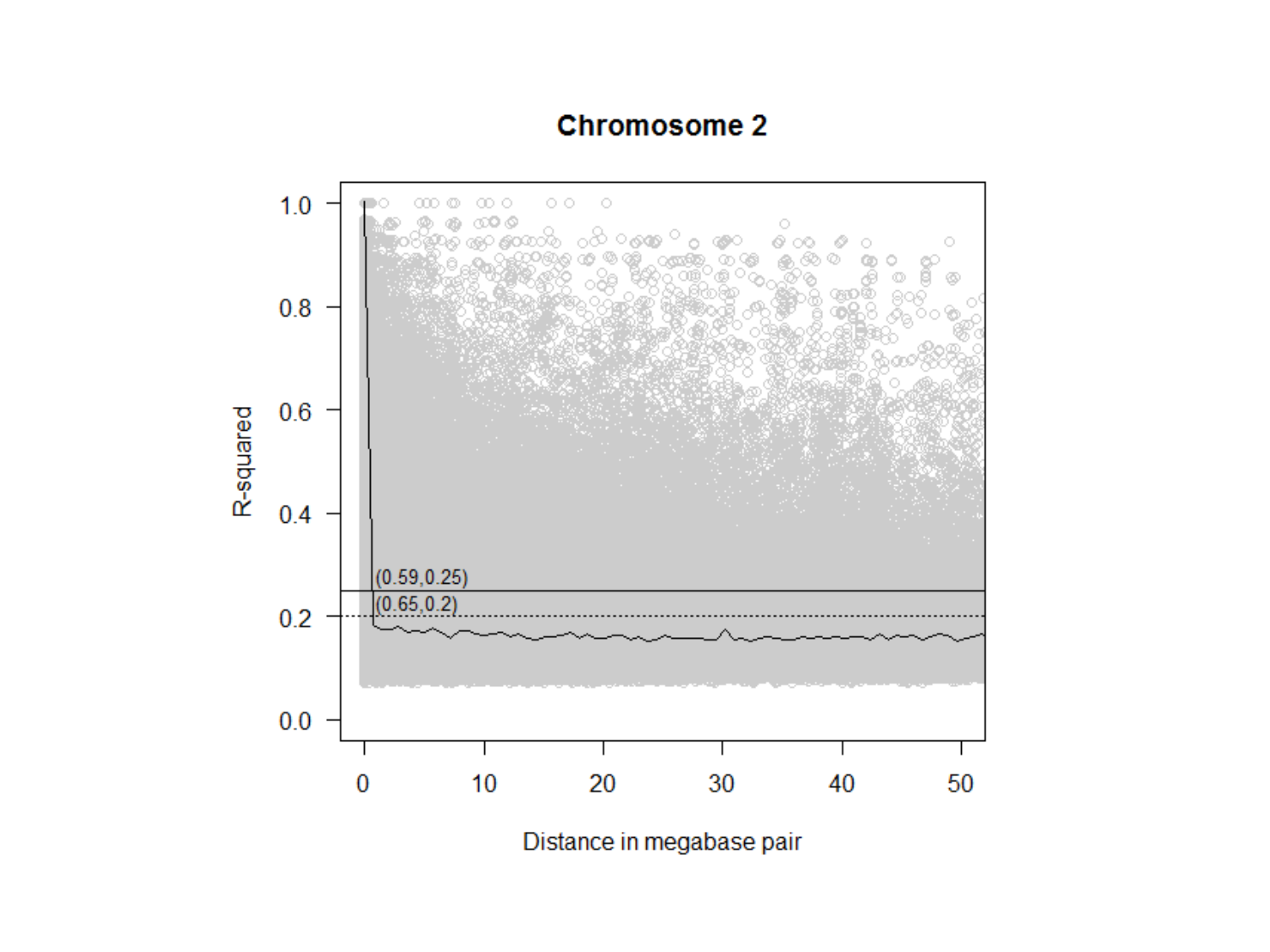

## Slide 4
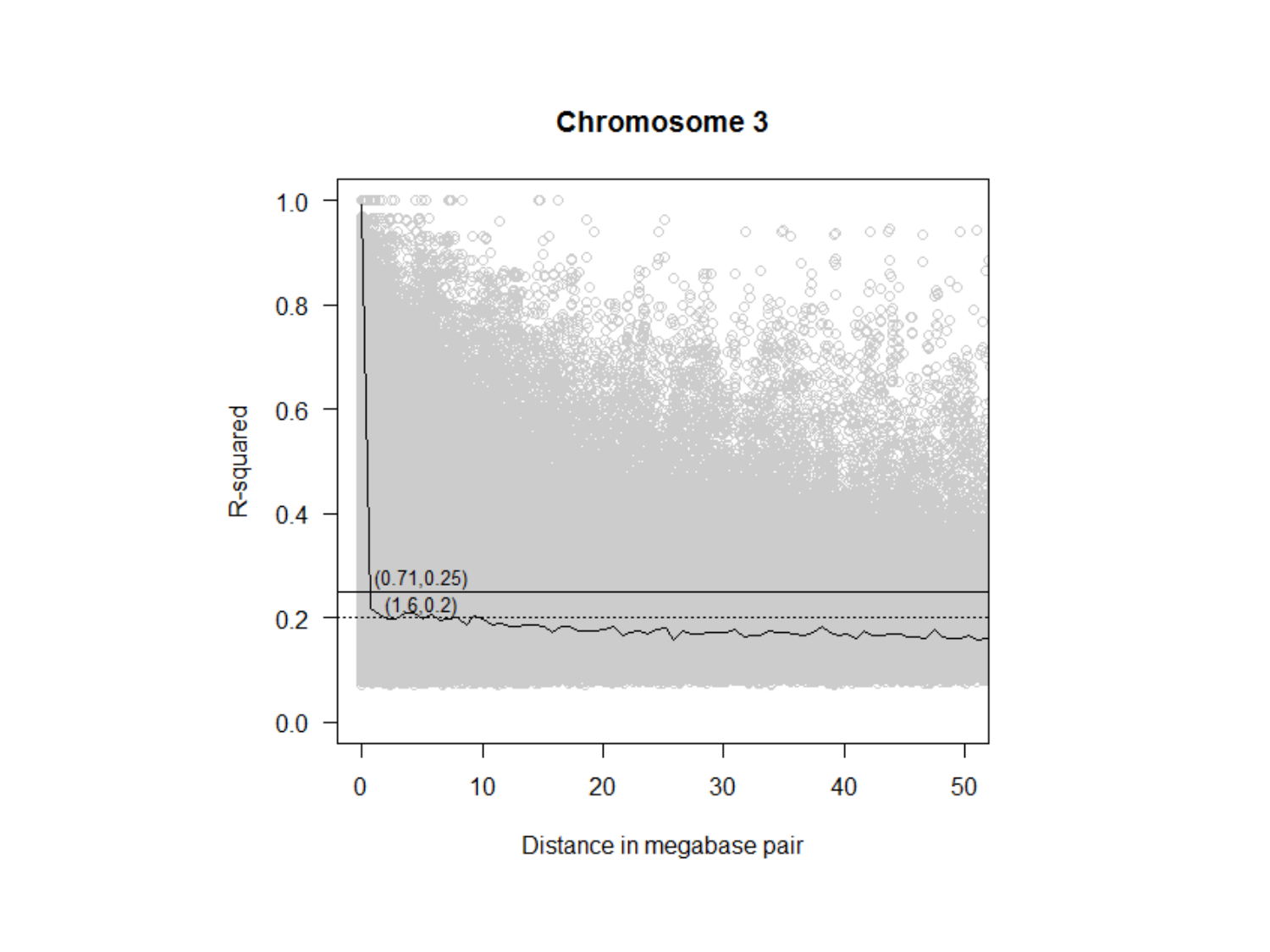

## Slide 5
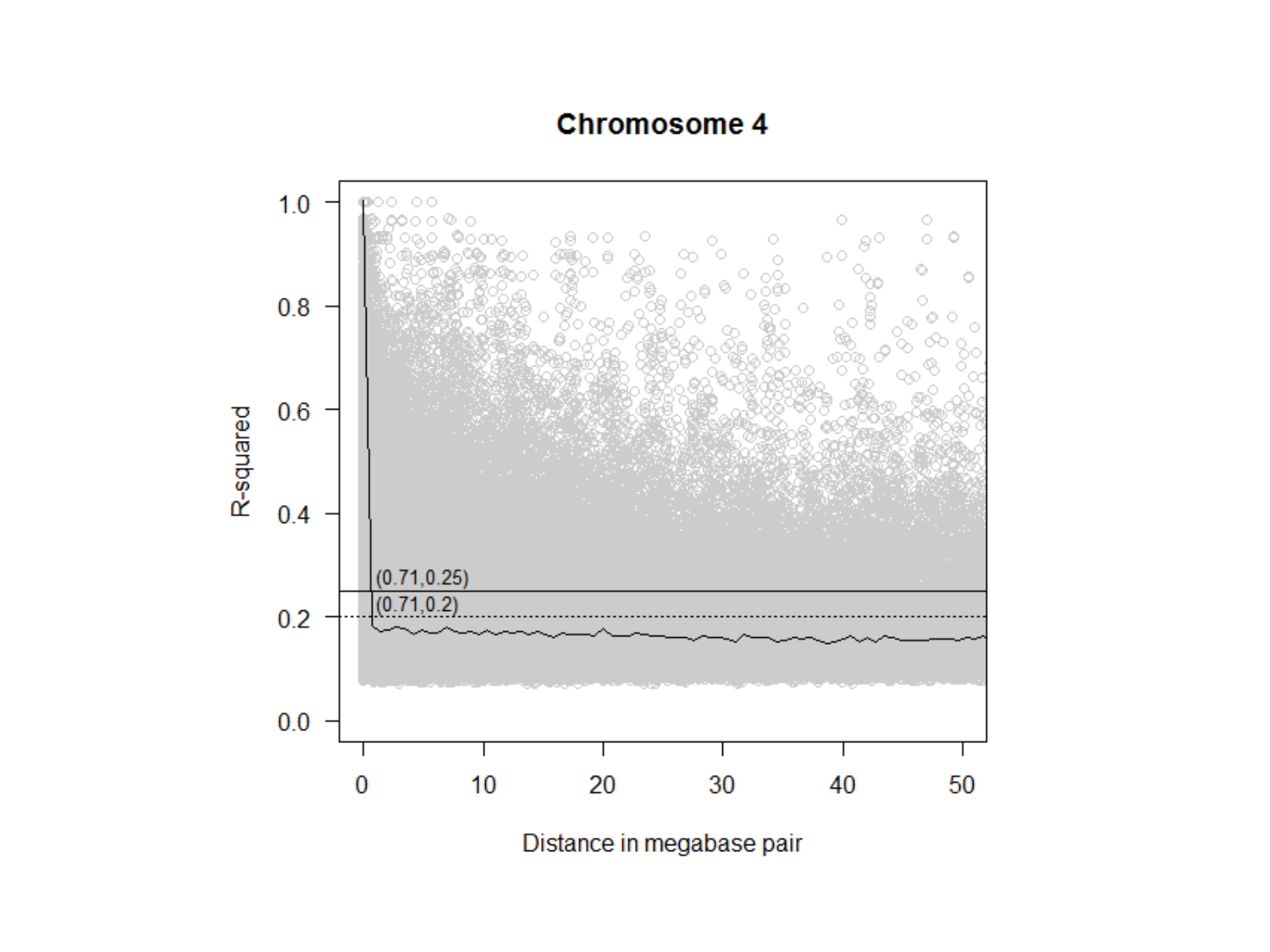

## Slide 6
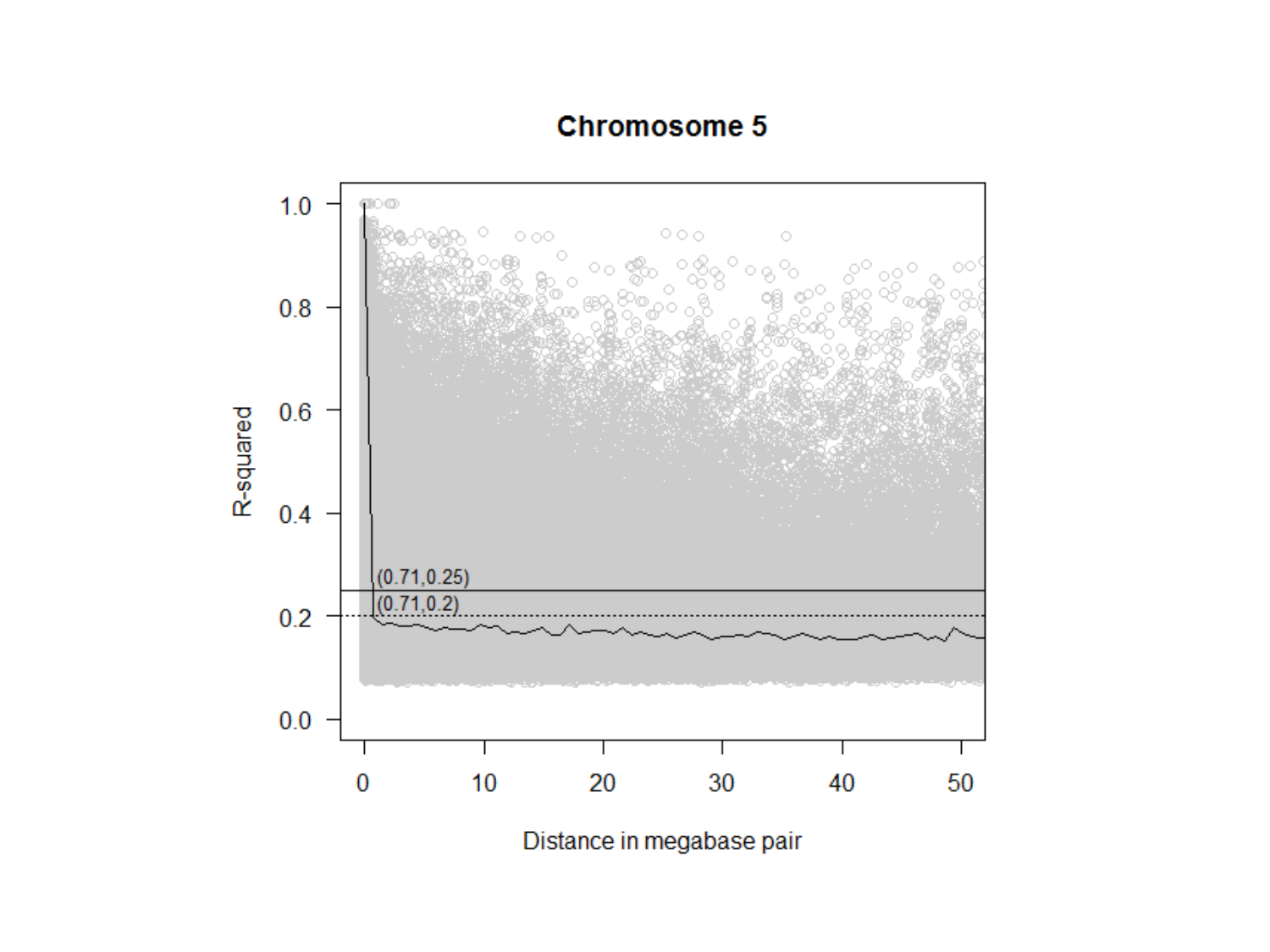

## Slide 7
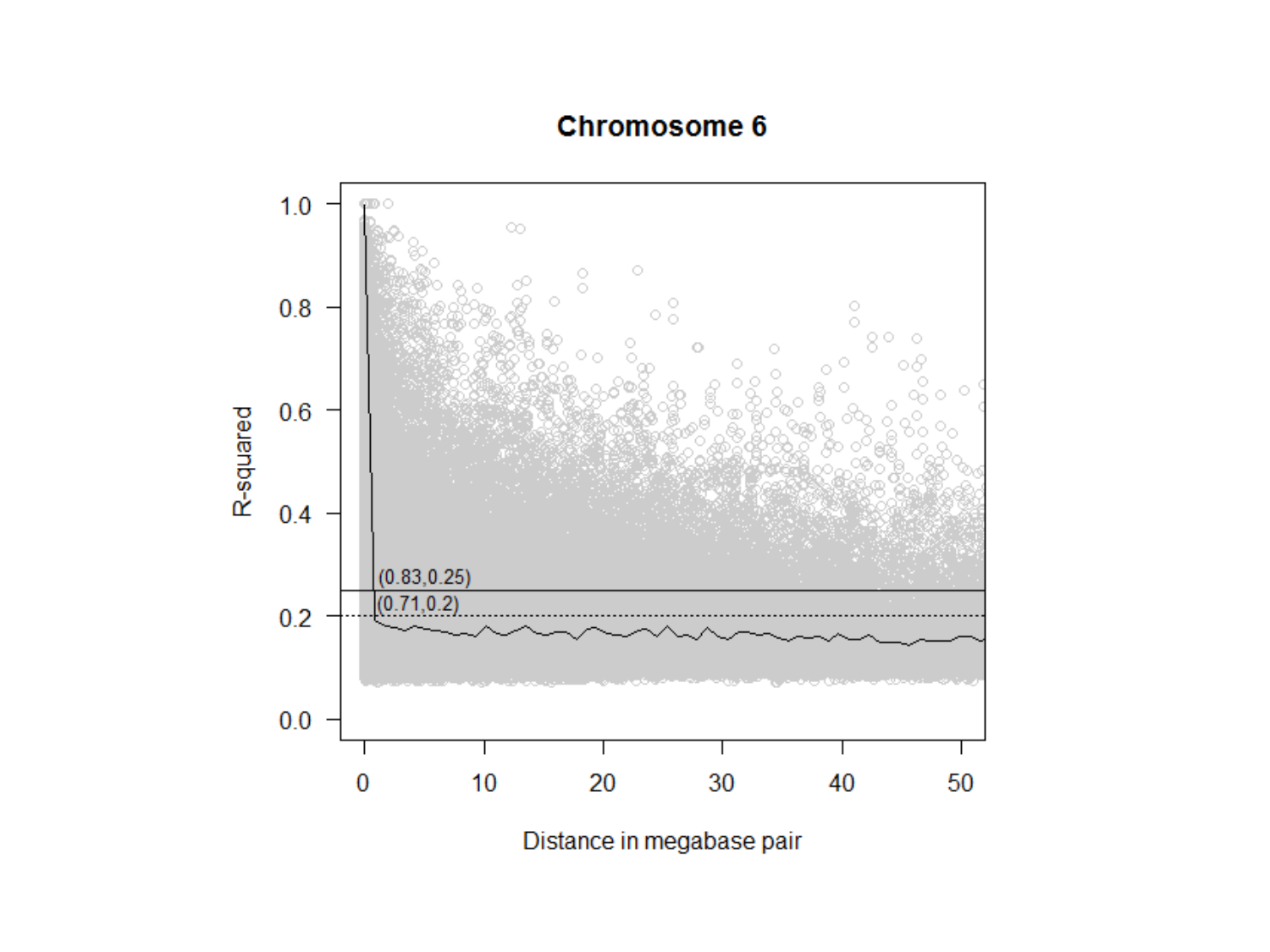

## Slide 8
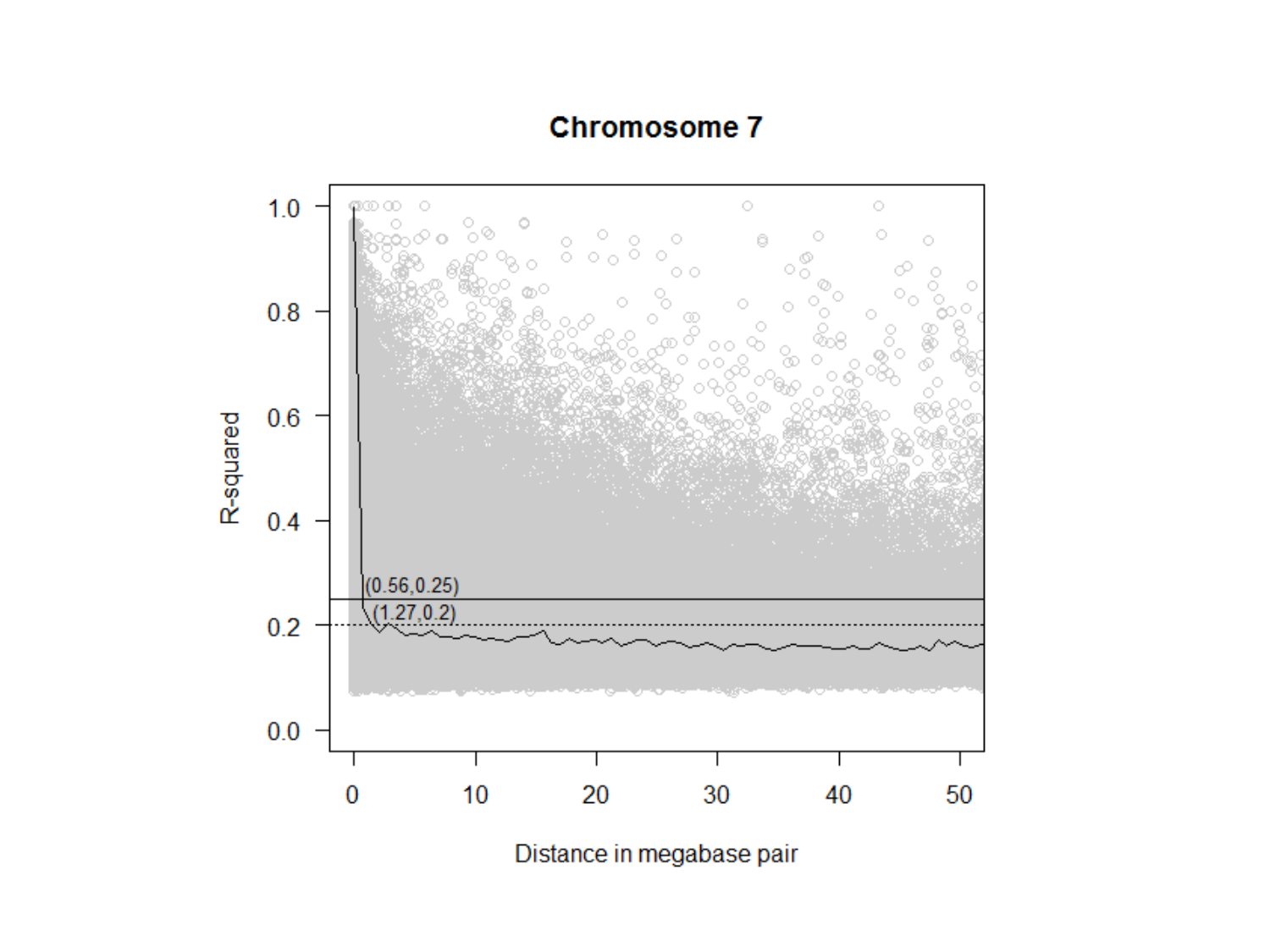

## Slide 9
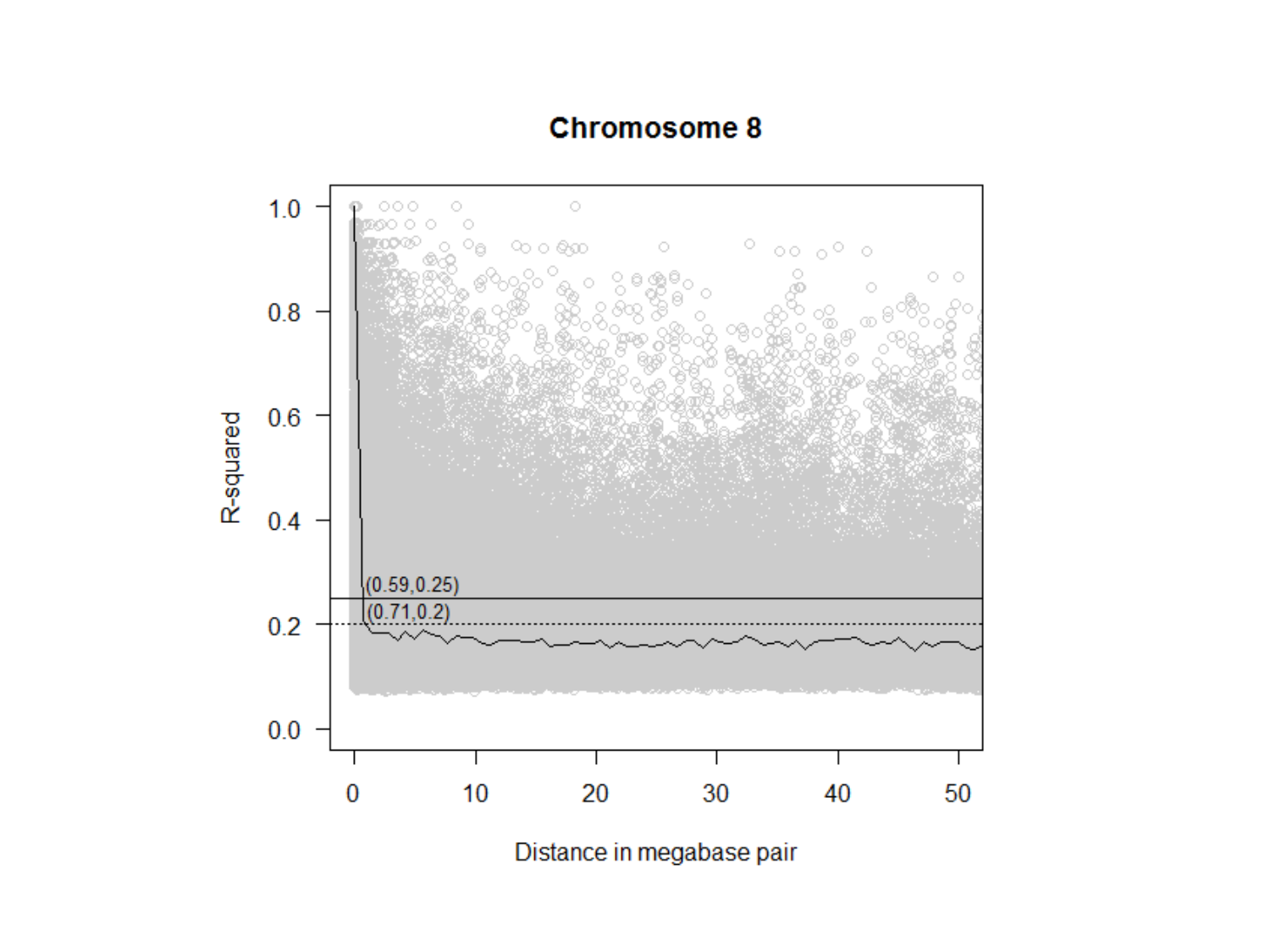

## Slide 10
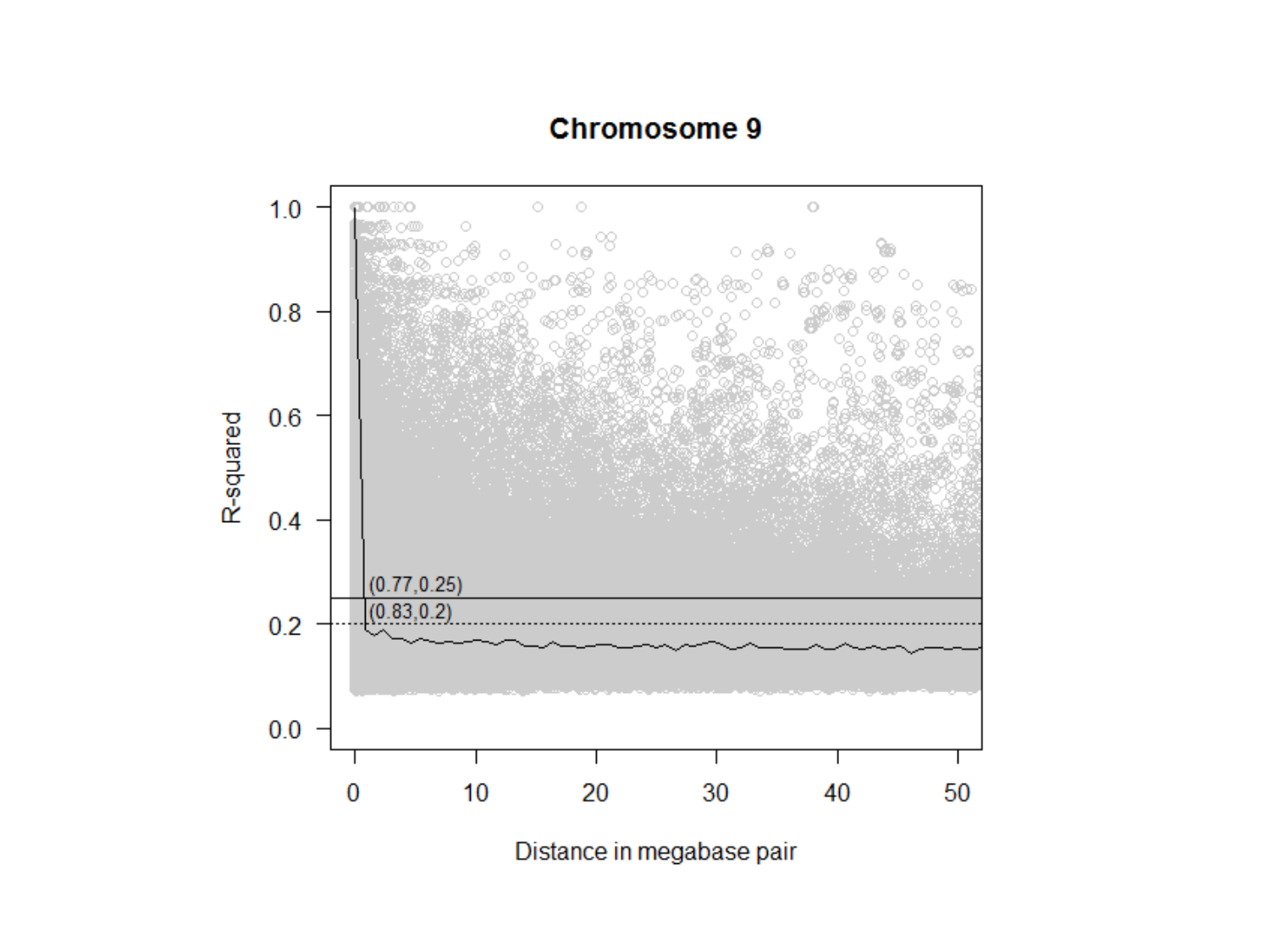

## Slide 11
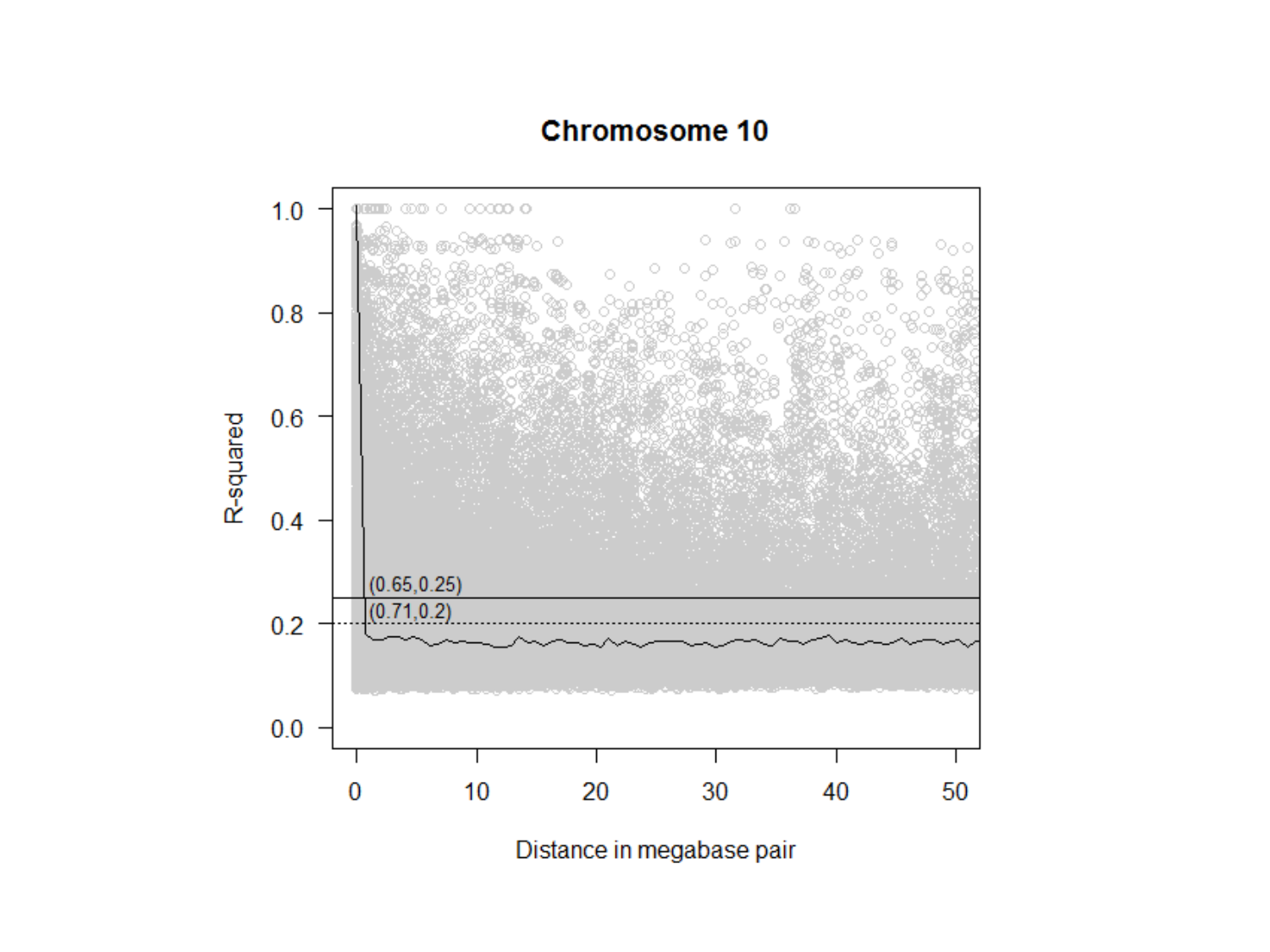

Supplement: Supplementary file 4 [file 1049FigureS4.pptx]

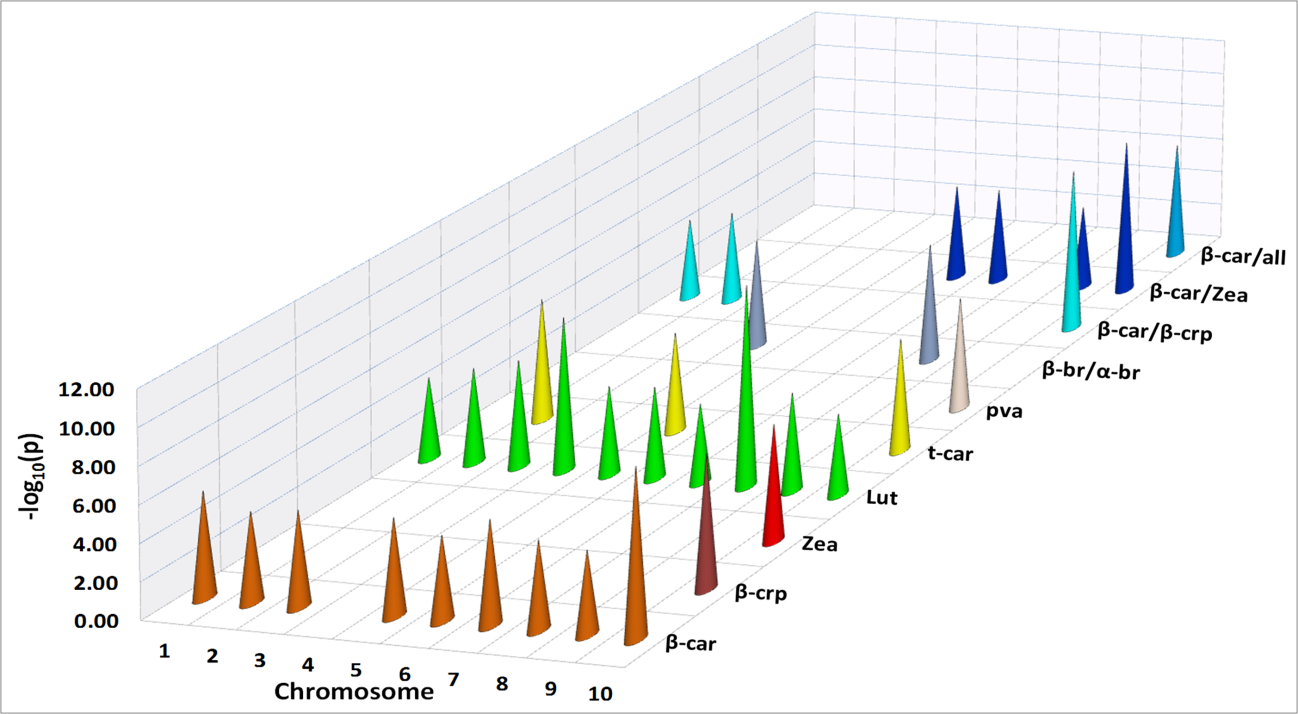

Supplement: Supplementary file 5 [file 1049FigureS5.tif]
