# Supplementary material for: Genetic Loci Controlling Carotenoid Biosynthesis in Diverse Tropical Maize Lines
Source: G3 (Bethesda). 2018 Jan 29;8(3):1049–65. doi: 10.1534/g3.117.300511 (PMC5844293; doi:10.1534/g3.117.300511)
Supplement: Supplementary file 7 [file 1049FileS3.docx]

### Supplementary Information

### SNP diversity within the GBS genotyped IITA tropical maize panel

The summary of the 110 k SNP data set used for the GWAS and its diversity parameters are presented in Supplementary Table 1. The average missing data for this data set was 10%. SNP distribution across the genome was not uniform but attained significant coverage (Supplementary Figure 1). Minor allele frequencies (MAF) displayed a uniform distribution across the 10 maize chromosomes (average = 0.13 to 0.14, median = 0.06 to 0.8). The rare allele frequencies (< 0.05) represented the largest proportion of the minor allele frequencies (Supplementary Figure 2). The average inbreeding coefficient (f) estimates per locus ranged from below zero to one, indicating the variable level of inbreeding across the genome, while the genome-wide mean f was 0.82. The heterozygosity (H) of the lines varied from 0.02 to 0.13, with an average of 0.05. More than half of the inbred lines showed less than 0.04 H values. Both H and f had more or less uniform values across the chromosomes. The genome-wide polymorphic information content (PIC) of the SNPs ranged from 0.02 to 0.38, while the average was 0.18.

### Population structure and kinship within the IITA tropical maize panel

The first three Principal Components (PCs) accounted for 21% of the variation, thus the first 15 PCs were considered for the variance to reach 50% (Table 2). On the basis of BIC model selection the optimum number of principal components (PC) Q (population structure) to account for the variation in the various carotenoids was either no PC or two PCs (Supplementary Table 2). This demonstrated the contribution of genome-wide population structure to the variation in carotenoid profile of the maize panel based on PCA. As the association panel included maize inbred lines derived from several bi-parental and backcross progenies, relatedness was expected among individuals derived from crosses involving common parent(s) (Azmach et al., 2013). The kinship heat-map for the panel (Supplementary Figure 3) illustrated that the majority of the inbred lines had kinship values below 0.5 suggesting the low level of overall relatedness in the panel. The counts of kinship values started to rise from near zero, peaked when approaching kinship value of 0.5 and quickly declined before reaching 1 and stayed constant just above zero until a kinship value of two, demonstrating the variable level of relatedness across groups of inbred lines in the panel.

**Linkage disequilibrium (LD)**

Genome-wide LD analysis was performed using the 1658 SNPs filtered from the large GBS dataset at 0% missing and 10% minimum MAF. Full matrix LD analysis of this dataset in TASSEL 3 involved 1.3 million pairwise correlations, 10% of which resulted in significant R^2^ at *P<*0.001 (Table 3). The genome-wide extent of LD estimate was 0.83 Mbp at baseline R^2^ = 0.2 and 0.65 Mbp at R^2^ = 0.25 (Table 3, Supplementary Figure S4). A heterogeneous distribution of LD was observed in the pattern of the LD heat-map generated using the same SNP dataset (Figure 1) (Flint-Garcia et al., 2003). The LD for each chromosome ranged from 0.65 Mbp on chromosomes 2 to 1.6 Mbp on Chromosome 3, when considering 0.2 as a baseline critical R^2^. On the other hand, at the calculated critical R^2^ = 0.25, the LD decay across chromosomes was less variable ranging from 0.56 Mbp on chromosome 7, 0.59 on chromosome 2 and 8, 0.65 on chromosome 10, 0.71 on chromosomes 1, 3, 4, 5 and 6, to 0.77 on chromosome 9.

**Supplementary Table ‎1** SNP data summary and basic diversity parameters for SNP dataset used in the GWAS

|  |  | **Chr1** | **Chr2** | **Chr3** | **Chr4** | **Chr5** | **Chr6** | **Chr7** | **Chr8** | **Chr9** | **Chr10** | **GW** |
| --- | --- | --- | --- | --- | --- | --- | --- | --- | --- | --- | --- | --- |
| **Total SNPs** | - | 17,743 | 13,023 | 11,905 | 11,606 | 12,941 | 8,557 | 9,424 | 9,373 | 8,008 | 7,357 | 109,937 |
| **Missing (%)** | - | 0.10 | 0.10 | 0.10 | 0.10 | 0.10 | 0.10 | 0.10 | 0.10 | 0.10 | 0.10 | 0.10 |
| **Avg. distance between successive SNPs in Mbp** | Max | 1.7 | 2.0 | 1.4 | 1.8 | 1.4 | 1.3 | 1.5 | 2.9 | 1.3 | 0.8 | 2.9 |
|  | Mean | 0.017 | 0.018 | 0.019 | 0.021 | 0.017 | 0.020 | 0.019 | 0.019 | 0.020 | 0.020 | 0.019 |
| **MAF** | Mean | 0.13 | 0.14 | 0.13 | 0.13 | 0.13 | 0.13 | 0.12 | 0.13 | 0.14 | 0.13 | 0.13 |
|  | median | 0.07 | 0.08 | 0.06 | 0.06 | 0.06 | 0.06 | 0.06 | 0.07 | 0.08 | 0.07 | 0.07 |
| **PIC** | Min | 0.02 | 0.02 | 0.02 | 0.06 | 0.02 | 0.02 | 0.02 | 0.02 | 0.02 | 0.02 | 0.02 |
|  | Max | 0.38 | 0.38 | 0.38 | 0.20 | 0.38 | 0.38 | 0.38 | 0.38 | 0.38 | 0.38 | 0.38 |
|  | Mean | 0.18 | 0.19 | 0.17 | 0.18 | 0.19 | 0.16 | 0.15 | 0.18 | 0.19 | 0.17 | 0.18 |
|  | Median | 0.16 | 0.17 | 0.13 | 0.16 | 0.17 | 0.13 | 0.11 | 0.17 | 0.17 | 0.13 | 0.15 |
| **f** | Min | -0.26 | -0.28 | -0.67 | -0.13 | -0.70 | -0.78 | -0.44 | -0.92 | -0.83 | -0.44 | -0.92 |
|  | Max | 1.00 | 1.00 | 1.00 | 1.00 | 1.00 | 1.00 | 1.00 | 1.00 | 1.00 | 1.00 | 1.00 |
|  | Mean | 0.78 | 0.75 | 0.68 | 0.81 | 0.74 | 0.73 | 0.72 | 0.67 | 0.71 | 0.71 | 0.82 |
|  | Median | 0.89 | 0.88 | 0.85 | 0.89 | 0.87 | 0.85 | 0.87 | 0.86 | 0.88 | 0.87 | 0.87 |
| **H** | Min | 0.02 | 0.02 | 0.01 | 0.02 | 0.01 | 0.02 | 0.01 | 0.02 | 0.02 | 0.02 | 0.02 |
|  | Max | 0.12 | 0.14 | 0.15 | 0.13 | 0.12 | 0.13 | 0.14 | 0.14 | 0.15 | 0.16 | 0.13 |
|  | Mean | 0.04 | 0.05 | 0.05 | 0.05 | 0.04 | 0.05 | 0.14 | 0.05 | 0.05 | 0.05 | 0.05 |
|  | median | 0.04 | 0.04 | 0.04 | 0.04 | 0.04 | 0.04 | 0.04 | 0.04 | 0.05 | 0.04 | 0.03 |

Chr, Chromosome; GW, Genome-wide; MAF, MAF; Min, Minimum; Max, Maximum; PIC, polymorphic information content; f, inbreeding coefficient; and H, heterozygousity,

**H** was estimated in TASSEL, per inbred line basis

**PIC** , and **f** were calculated in PowerMarker using 3532 SNPs without missing data point and 1% MAF.

**Supplementary Table 2** Optimum number of the first PCs selected based on BIC

| Carotenoid | No. of PCs selected based on BIC |
| --- | --- |
| β-carotene | 2 |
| β -cryptoxanthin | 0 |
| Lutein | 0 |
| Zeaxanthin | 0 |
| Total carotenoid | 0 |
| Provitamin A | 2 |
| Lnβ-branch/α-branch | 0 |
| Lnβ-carotene/zeaxanthin | 2 |
| Lnβ-carotene/All | 2 |
| Lnβ-carotene/β-cryptoxanthin | 2 |

Supplementary Table 3. Ranges of carotenoids least square means in µg/g dry weight of the the 130 inbred lines evaluated for two seasons (2010 - 2011

|  | **lut** | **zeax** | **β-cryp** | **α-car** | **β-car** | **tpva** | **tcar** |
| --- | --- | --- | --- | --- | --- | --- | --- |
| **Minimum** | 0.45 | 0.04 | 0.08 | 0 | 0.03 | 0.06 | 4.43 |
| **Maximum** | 13.51 | 25.9 | 8.55 | 1.68 | 16.38 | 17.25 | 42.71 |
| **Grand Mean** | 3.58 | 9.66 | 2.92 | 0.4 | 4.21 | 5.87 | 20.78 |

βcar, β-carotene; βcryp, β-cryptoxanthin; lut, lutein; zea, zeaxanthin; pva, provitamin A; tcar, total carotenoid

Supplementary Table 4 Genes within LD range of the most significant SNPs detected by the GWAS (with/without allele specific markers as covariates)

| **Gene stable ID** | **Gene name** | **Gene description** | **Chr** | **Strand** | **Coordinate** | | **Most significant SNP** | **Distance from gene (Mbp)** |
| --- | --- | --- | --- | --- | --- | --- | --- | --- |
|  |  |  |  |  | **start** | **end** |  |  |
| GRMZM2G021710 |  |  | 2 | -1 | 35,273,097 | 35,277,507 |  | 799,874 |
| GRMZM2G023204 |  |  | 2 | -1 | 35,324,876 | 35,326,785 |  | 750,596 |
| GRMZM2G389789 |  | Uncharacterized protein | 2 | 1 | 35,355,453 | 35,360,661 |  | 716,720 |
| AC211676.4_FG001 |  |  | 2 | -1 | 35,482,452 | 35,482,829 |  | 594,552 |
| GRMZM2G448126 |  |  | 2 | 1 | 35,482,483 | 35,484,426 |  | 592,955 |
| GRMZM2G099817 |  | Uncharacterized protein | 2 | 1 | 35,568,928 | 35,570,039 |  | 507,342 |
| GRMZM2G703008 |  |  | 2 | -1 | 35,620,406 | 35,620,868 |  | 456,513 |
| GRMZM2G152000 |  | Zinc finger, C3HC4 type family protein | 2 | 1 | 35,674,894 | 35,676,093 |  | 401,288 |
| GRMZM2G429662 |  | Zinc finger, C3HC4 type family protein | 2 | -1 | 35,746,325 | 35,748,257 |  | 329,124 |
| AC214613.3_FG003 |  |  | 2 | -1 | 35,790,943 | 35,792,868 |  | 284,513 |
| GRMZM2G128800 |  | Zinc finger, C3HC4 type family protein | 2 | -1 | 35,841,329 | 35,842,533 |  | 234,848 |
| GRMZM2G054046 |  |  | 2 | 1 | 35,876,928 | 35,877,532 |  | 199,849 |
| GRMZM2G703013 |  |  | 2 | 1 | 35,877,646 | 35,878,504 |  | 198,877 |
| AC193754.3_FG008 |  |  | 2 | -1 | 35,880,818 | 35,882,857 |  | 194,524 |
| GRMZM2G053929 |  | Uncharacterized protein | 2 | -1 | 35,884,125 | 35,885,404 |  | 191,977 |
| GRMZM2G099183 |  | Uncharacterized protein | 2 | 1 | 35,949,719 | 35,954,263 |  | 123,118 |
| AC193754.3_FG005 |  |  | 2 | -1 | 36,034,684 | 36,035,349 |  | 42,032 |
| AC186613.4_FG003 |  |  | 2 | -1 | 36,039,757 | 36,040,371 |  | 37,010 |
| GRMZM2G021619 |  |  | 2 | -1 | 36,079,677 | 36,082,153 | S2_36077381 | 2,296 |
| GRMZM2G030598 |  | Kelch motif family protein | 2 | -1 | 36,133,981 | 36,154,824 |  | 56,600 |
| GRMZM2G102183 | *LIP* | Malate synthase, glyoxysomal | 2 | 1 | 36,317,292 | 36,319,768 |  | 239,911 |
| GRMZM2G102238 |  | Acid phosphatase/vanadium-dependent haloperoxidase related | 2 | -1 | 36,320,023 | 36,330,061 |  | 242,642 |
| GRMZM2G112072 |  | Putative uncharacterized protein | 2 | 1 | 36,358,387 | 36,360,987 |  | 281,006 |
| GRMZM2G130109 |  | Uncharacterized protein | 2 | 1 | 36,445,644 | 36,449,142 |  | 368,263 |
| GRMZM2G130224 |  |  | 2 | -1 | 36,450,557 | 36,453,156 |  | 373,176 |
| GRMZM2G130230 |  | Glucose-6-phosphate 1-dehydrogenase | 2 | -1 | 36,456,199 | 36,464,613 |  | 378,818 |
| GRMZM2G142712 |  | Uncharacterized protein | 2 | -1 | 36,563,910 | 36,568,978 |  | 486,529 |
| GRMZM2G093832 |  |  | 2 | 1 | 36,573,079 | 36,579,034 |  | 495,698 |
| GRMZM2G093781 |  |  | 2 | -1 | 36,582,037 | 36,585,511 |  | 504,656 |
| GRMZM2G093720 |  |  | 2 | 1 | 36,586,610 | 36,591,326 |  | 509,229 |
| GRMZM2G093557 |  | 26S proteasome non-ATPase regulatory subunit 6 | 2 | -1 | 36,591,474 | 36,594,714 |  | 514,093 |
| GRMZM2G100872 |  | Uncharacterized protein | 2 | 1 | 36,597,705 | 36,602,732 |  | 520,324 |
| GRMZM2G100815 |  |  | 2 | -1 | 36,605,205 | 36,613,857 |  | 527,824 |
| GRMZM2G372930 |  |  | 2 | -1 | 36,617,989 | 36,618,665 |  | 540,608 |
| GRMZM2G063262 |  |  | 2 | 1 | 36,763,535 | 36,768,059 |  | 686,154 |
| GRMZM2G063163 |  | Uncharacterized protein | 2 | -1 | 36,767,527 | 36,778,587 |  | 690,146 |
| GRMZM2G045117 |  | BCL-2 binding anthanogene-1 | 2 | -1 | 36,824,114 | 36,825,732 |  | 746,733 |
| GRMZM2G045070 |  | Topoisomerase-like protein | 2 | 1 | 36,833,209 | 36,838,151 |  | 755,828 |
| GRMZM2G044989 |  | Uncharacterized protein | 2 | -1 | 36,838,775 | 36,842,537 |  | 761,394 |
| GRMZM2G094792 |  | Uncharacterized protein | 2 | -1 | 42,618,631 | 42,623,189 |  | 752,968 |
| GRMZM2G393762 |  |  | 2 | -1 | 42,633,247 | 42,637,017 |  | 739,140 |
| GRMZM2G427790 |  |  | 2 | -1 | 42,693,055 | 42,695,872 |  | 680,285 |
| GRMZM2G065073 |  | Gamma-glutamyltranspeptidase 1 | 2 | 1 | 42,784,980 | 42,792,494 |  | 583,663 |
| GRMZM2G522241 |  |  | 2 | 1 | 42,793,644 | 42,794,026 |  | 582,131 |
| GRMZM2G065157 |  | Putative uncharacterized protein | 2 | 1 | 42,794,940 | 42,795,548 |  | 580,609 |
| GRMZM2G065171 |  | Uncharacterized protein | 2 | 1 | 42,800,989 | 42,805,578 |  | 570,579 |
| GRMZM2G476822 |  |  | 2 | -1 | 42,919,265 | 42,931,636 |  | 444,521 |
| GRMZM2G044322 |  | Uncharacterized protein | 2 | -1 | 42,957,793 | 42,959,684 |  | 416,473 |
| GRMZM2G148147 |  | Putative uncharacterized protein | 2 | 1 | 43,000,189 | 43,001,179 |  | 374,978 |
| GRMZM2G119375 |  |  | 2 | -1 | 43,091,374 | 43,093,513 |  | 282,644 |
| GRMZM2G018275 |  | Uncharacterized protein | 2 | -1 | 43,117,505 | 43,119,018 |  | 257,139 |
| GRMZM2G071996 |  | Putative uncharacterized protein | 2 | 1 | 43,182,532 | 43,185,690 |  | 190,467 |
| GRMZM2G071768 |  | Serologically defined breast cancer antigen NY-BR-84 | 2 | 1 | 43,188,346 | 43,197,822 |  | 178,335 |
| GRMZM2G071714 | *LIP1* | Lipoyl synthase, mitochondrial | 2 | 1 | 43,200,299 | 43,203,078 |  | 173,079 |
| GRMZM2G071704 |  |  | 2 | 1 | 43,204,316 | 43,205,109 |  | 171,048 |
| GRMZM2G092107 |  | Putative uncharacterized protein | 2 | 1 | 43,339,685 | 43,346,040 |  | 30,117 |
| GRMZM2G442523 |  | Uncharacterized protein | 2 | 1 | 43,370,373 | 43,375,906 |  | 251 |
| GRMZM2G442546 |  | Uncharacterized protein | 2 | -1 | 43,375,177 | 43,378,906 | S2_43376157 | 980 |
| GRMZM2G031028 |  | Plastid-lipid-associated protein 2 | 2 | 1 | 43,428,306 | 43,433,991 |  | 52,149 |
| GRMZM5G843141 |  | Uncharacterized protein | 2 | -1 | 43,434,582 | 43,441,085 |  | 58,425 |
| GRMZM5G831135 |  |  | 2 | -1 | 43,480,267 | 43,481,433 |  | 104,110 |
| GRMZM2G360374 |  |  | 2 | 1 | 43,480,267 | 43,481,433 |  | 104,110 |
| GRMZM2G061495 |  |  | 2 | -1 | 43,482,588 | 43,483,408 |  | 106,431 |
| GRMZM2G010338 |  | Uncharacterized protein | 2 | 1 | 43,523,290 | 43,532,367 |  | 147,133 |
| GRMZM2G049568 |  | Ubiquitin carboxyl-terminal hydrolase | 2 | 1 | 43,549,463 | 43,557,488 |  | 173,306 |
| GRMZM2G049549 |  | Uncharacterized protein | 2 | -1 | 43,557,836 | 43,561,417 |  | 181,679 |
| GRMZM5G837621 |  |  | 2 | 1 | 43,579,798 | 43,580,388 |  | 203,641 |
| GRMZM2G171296 |  | Putative uncharacterized protein | 2 | -1 | 43,633,707 | 43,634,200 |  | 257,550 |
| GRMZM2G009125 |  | Cytokinin-O-glucosyltransferase 2 | 2 | -1 | 43,708,019 | 43,710,470 |  | 331,862 |
| AC194705.3_FG004 |  |  | 2 | 1 | 43,736,695 | 43,738,206 |  | 360,538 |
| GRMZM2G325139 |  |  | 2 | -1 | 43,760,551 | 43,761,190 |  | 712,611 |
| GRMZM5G870067 |  | Uncharacterized protein | 2 | -1 | 43,834,734 | 43,836,570 |  | 637,231 |
| GRMZM2G417770 |  |  | 2 | 1 | 43,943,602 | 43,945,578 |  | 528,223 |
| GRMZM2G024119 |  | Uncharacterized protein | 2 | -1 | 44,013,584 | 44,035,237 |  | 438,564 |
| GRMZM2G703021 |  | Uncharacterized protein | 2 | 1 | 44,038,259 | 44,040,528 |  | 433,273 |
| GRMZM2G145758 |  | Histone H3 | 2 | -1 | 44,167,696 | 44,170,031 |  | 303,770 |
| GRMZM2G470882 |  | Uncharacterized protein | 2 | -1 | 44,187,637 | 44,189,354 |  | 284,447 |
| GRMZM2G171254 |  | Uncharacterized protein | 2 | -1 | 44,190,672 | 44,202,611 |  | 271,190 |
| GRMZM2G471039 |  |  | 2 | 1 | 44,193,630 | 44,195,023 |  | 278,778 |
| GRMZM2G171277 |  | Uncharacterized protein | 2 | 1 | 44,200,748 | 44,202,483 |  | 271,318 |
| GRMZM2G160966 |  |  | 2 | -1 | 44,260,729 | 44,262,323 |  | 211,478 |
| GRMZM2G160994 |  | Putative uncharacterized protein | 2 | -1 | 44,262,632 | 44,265,116 |  | 208,685 |
| GRMZM2G161012 |  |  | 2 | -1 | 44,274,679 | 44,276,416 |  | 197,385 |
| GRMZM2G167262 |  | Signal recognition particle 19 kDa protein | 2 | -1 | 44,298,898 | 44,304,717 |  | 169,084 |
| GRMZM2G046900 |  | Putative uncharacterized protein | 2 | -1 | 44,330,496 | 44,336,261 |  | 137,540 |
| GRMZM2G347488 |  | Putative uncharacterized protein | 2 | -1 | 44,337,299 | 44,338,263 |  | 135,538 |
| GRMZM2G347489 |  |  | 2 | 1 | 44,340,046 | 44,345,073 |  | 128,728 |
| GRMZM2G015844 |  | Uncharacterized protein | 2 | 1 | 44,378,673 | 44,382,847 |  | 90,954 |
| GRMZM2G015610 |  | Uncharacterized protein | 2 | 1 | 44,386,603 | 44,391,746 |  | 82,055 |
| GRMZM2G127184 |  |  | 2 | 1 | 44,434,561 | 44,436,541 |  | 37,260 |
| GRMZM2G127173 |  |  | 2 | -1 | 44,436,649 | 44,439,381 |  | 34,420 |
| GRMZM2G127139 | *ZEP1* | Zeaxanthin epoxidase | 2 | -1 | 44,440,299 | 44,449,237 | S2_44473801 | 24,564 |
| GRMZM2G062559 |  | Uncharacterized protein | 2 | -1 | 44,471,623 | 44,474,212 | S2_44473758 | 411 |
| GRMZM5G889338 |  | OVERLAPPING | 2 | 1 | 44,471,647 | 44,472,033 |  | 2,154 |
| GRMZM2G345238 |  |  | 2 | -1 | 44,547,174 | 44,552,558 |  | 73,373 |
| GRMZM2G046402 |  | Uncharacterized protein | 2 | -1 | 44,553,002 | 44,562,139 |  | 79,201 |
| GRMZM2G474783 |  |  | 2 | -1 | 44,697,029 | 44,698,753 |  | 223,228 |
| GRMZM2G053985 |  |  | 2 | 1 | 44,719,043 | 44,719,955 |  | 245,242 |
| GRMZM2G355906 |  | Uncharacterized protein | 2 | -1 | 44,720,554 | 44,751,914 |  | 246,753 |
| GRMZM2G126062 |  | Aldose reductase | 2 | -1 | 44,795,877 | 44,798,393 |  | 322,076 |
| GRMZM2G051355 |  | Uncharacterized protein | 2 | 1 | 44,854,880 | 44,861,495 |  | 381,079 |
| GRMZM2G103050 |  |  | 2 | 1 | 44,928,620 | 44,937,597 |  | 454,819 |
| GRMZM2G408038 |  |  | 2 | 1 | 44,941,098 | 44,944,904 |  | 467,297 |
| GRMZM2G101069 |  | Glutamate decarboxylase | 2 | 1 | 44,991,322 | 44,994,248 |  | 517,521 |
| GRMZM2G104843 |  | Lipoxygenase | 2 | -1 | 45,192,092 | 45,196,460 |  | 718,291 |
| GRMZM2G105542 |  | Uncharacterized protein | 2 | -1 | 45,219,705 | 45,222,039 |  | 745,904 |
| GRMZM2G105608 |  |  | 2 | -1 | 45,223,315 | 45,224,127 |  | 749,514 |
| GRMZM2G105617 |  |  | 2 | 1 | 45,230,433 | 45,231,167 |  | 756,632 |
| GRMZM2G090675 |  | Uncharacterized protein | 2 | -1 | 102,897,151 | 102,905,974 |  | 784128 |
| GRMZM2G019742 |  |  | 2 | -1 | 103,027,499 | 103,028,664 |  | 653780 |
| GRMZM2G019819 |  |  | 2 | 1 | 103,045,720 | 103,049,593 |  | 635559 |
| GRMZM2G109873 |  |  | 2 | 1 | 103,171,373 | 103,172,289 |  | 509906 |
| GRMZM2G109879 |  |  | 2 | 1 | 103,172,610 | 103,179,072 |  | 508669 |
| GRMZM2G365957 |  |  | 2 | -1 | 103,336,613 | 103,342,858 |  | 344666 |
| GRMZM2G303995 |  |  | 2 | -1 | 103,451,158 | 103,452,508 |  | 230121 |
| GRMZM2G303993 |  |  | 2 | -1 | 103,499,700 | 103,500,770 |  | 181579 |
| AC196719.3_FG002 |  |  | 2 | 1 | 103,500,310 | 103,500,681 |  | 180969 |
| GRMZM2G003796 |  |  | 2 | 1 | 103,532,762 | 103,534,648 |  | 148517 |
| GRMZM2G334321 |  |  | 2 | 1 | 103,668,965 | 103,670,087 |  | 12314 |
| GRMZM2G334336 |  |  | 2 | -1 | 103,680,545 | 103,682,621 | S2_103681279 | 734 |
| GRMZM2G334338 |  |  | 2 | -1 | 103,685,681 | 103,686,659 |  | 5,380 |
| GRMZM2G397965 |  |  | 2 | -1 | 103,825,178 | 103,826,833 |  | 145,554 |
| GRMZM2G089010 |  | Uncharacterized protein | 2 | 1 | 103,960,946 | 103,965,539 |  | 284,260 |
| AC190677.3_FG003 |  |  | 2 | -1 | 104,040,064 | 104,040,735 |  | 359,456 |
| GRMZM2G172686 |  |  | 2 | -1 | 104,167,615 | 104,170,477 |  | 489,198 |
| GRMZM2G172695 |  |  | 2 | 1 | 104,173,790 | 104,176,088 |  | 494,809 |
| GRMZM2G132968 |  | 60S ribosomal protein L3 | 2 | 1 | 138,864,214 | 138,867,222 |  | 777,054 |
| GRMZM2G132966 |  |  | 2 | 1 | 138,892,765 | 138,893,204 |  | 751,072 |
| AC204953.3_FG002 |  |  | 2 | -1 | 138,906,162 | 138,906,854 |  | 737,422 |
| GRMZM5G854138 |  | Uncharacterized protein | 2 | 1 | 139,066,950 | 139,068,044 |  | 576,232 |
| GRMZM2G486618 |  |  | 2 | -1 | 139,175,046 | 139,175,672 |  | 468,604 |
| GRMZM2G047292 |  | Stachyose synthase | 2 | -1 | 139,185,152 | 139,187,472 |  | 456,804 |
| GRMZM2G348151 |  | Uncharacterized protein | 2 | -1 | 139,433,415 | 139,435,902 |  | 208,374 |
| GRMZM2G099502 |  |  | 2 | 1 | 139,516,746 | 139,518,995 |  | 125,281 |
| GRMZM2G066213 |  |  | 2 | -1 | 139,643,865 | 139,645,573 | S2_139644276 | 411 |
| GRMZM2G366802 |  |  | 2 | 1 | 139,653,617 | 139,654,676 |  | 9,341 |
| GRMZM2G522398 |  |  | 2 | 1 | 139,658,407 | 139,662,410 |  | 14,131 |
| GRMZM2G039648 |  |  | 2 | 1 | 139,743,422 | 139,745,283 |  | 99,146 |
| GRMZM5G898141 |  |  | 2 | 1 | 139,759,830 | 139,764,442 |  | 115,554 |
| GRMZM2G144885 |  |  | 2 | -1 | 139,902,080 | 139,902,696 |  | 257,804 |
| AC205544.3_FG004 |  |  | 2 | -1 | 140,056,166 | 140,057,095 |  | 411,890 |
| GRMZM2G006765 |  | Uncharacterized protein | 2 | 1 | 140,057,140 | 140,059,599 |  | 412,864 |
| GRMZM2G010282 |  | Uncharacterized protein | 2 | -1 | 140,089,814 | 140,091,612 |  | 445,538 |
| AC226536.2_FG001 |  |  | 2 | 1 | 140,306,755 | 140,307,437 |  | 662,479 |
| GRMZM2G125352 |  | Uncharacterized protein | 2 | -1 | 140,309,065 | 140,313,923 |  | 664,789 |
| GRMZM5G821024 |  | Uncharacterized protein | 2 | -1 | 140,322,684 | 140,326,990 |  | 678,408 |
| GRMZM2G014400 |  |  | 2 | 1 | 207,955,688 | 207,967,618 |  | 705,060 |
| GRMZM2G112954 |  |  | 2 | 1 | 207,974,613 | 207,977,249 |  | 695,429 |
| GRMZM2G034927 |  |  | 2 | -1 | 208,075,008 | 208,076,470 |  | 596,208 |
| GRMZM2G034764 |  | C2 domain containing protein | 2 | 1 | 208,079,590 | 208,081,944 |  | 590,734 |
| GRMZM2G113848 |  |  | 2 | -1 | 208,114,241 | 208,115,156 |  | 557,522 |
| GRMZM2G403800 |  | Uncharacterized protein | 2 | -1 | 208,119,426 | 208,120,531 |  | 552,147 |
| GRMZM2G105207 |  | NADH-ubiquinone oxidoreductase 18 kDa subunit | 2 | 1 | 208,123,671 | 208,129,714 |  | 542,964 |
| GRMZM2G105302 |  | Putative uncharacterized protein | 2 | -1 | 208,128,674 | 208,130,991 |  | 541,687 |
| GRMZM2G351417 |  |  | 2 | 1 | 208,195,862 | 208,203,005 |  | 469,673 |
| GRMZM5G881349 |  |  | 2 | -1 | 208,203,708 | 208,204,361 |  | 468,317 |
| GRMZM2G003937 |  | Uncharacterized protein | 2 | 1 | 208,205,739 | 208,206,806 |  | 465,872 |
| GRMZM2G129575 |  |  | 2 | 1 | 208,223,142 | 208,227,514 |  | 445,164 |
| GRMZM2G454474 |  | Triacylglycerol lipase | 2 | -1 | 208,329,261 | 208,330,419 |  | 342,259 |
| GRMZM2G575305 |  | Putative uncharacterized protein | 2 | 1 | 208,345,176 | 208,349,036 |  | 323,642 |
| GRMZM2G021151 |  |  | 2 | 1 | 208,361,028 | 208,361,925 |  | 310,753 |
| GRMZM2G019060 |  |  | 2 | 1 | 208,376,585 | 208,378,677 |  | 294,001 |
| GRMZM2G028905 |  | Uncharacterized protein | 2 | -1 | 208,433,511 | 208,437,003 |  | 235,675 |
| GRMZM2G090274 |  | Eukaryotic peptide chain release factor subunit 1-1 | 2 | -1 | 208,530,771 | 208,535,295 |  | 137,383 |
| GRMZM2G090493 |  | Uncharacterized protein | 2 | 1 | 208,538,326 | 208,540,184 |  | 132,494 |
| GRMZM2G307604 |  |  | 2 | 1 | 208,569,032 | 208,571,129 |  | 101,549 |
| GRMZM2G007060 |  | Uncharacterized protein | 2 | 1 | 208,574,549 | 208,579,842 |  | 92,836 |
| GRMZM2G082612 |  | Uncharacterized protein | 2 | 1 | 208,609,666 | 208,612,318 |  | 60,360 |
| GRMZM2G383841 |  |  | 2 | -1 | 208,616,426 | 208,617,726 |  | 54,952 |
| GRMZM5G865483 |  |  | 2 | 1 | 208,653,141 | 208,653,828 |  | 18,850 |
| AC211702.2_FG002 |  |  | 2 | 1 | 208,654,679 | 208,656,244 |  | 16,434 |
| GRMZM5G823629 |  | Uncharacterized protein | 2 | 1 | 208,669,032 | 208,674,022 | S2_208672678 | 3,646 |
| GRMZM2G455889 |  |  | 2 | -1 | 208,675,050 | 208,677,330 |  | 2372 |
| GRMZM5G824534 |  | Putative uncharacterized protein | 2 | 1 | 208,845,445 | 208,851,248 |  | 172767 |
| GRMZM2G177110 |  | Seed specific protein Bn15D17A | 2 | 1 | 208,854,344 | 208,855,480 |  | 181666 |
| GRMZM2G125501 |  |  | 2 | 1 | 208,881,462 | 208,883,455 |  | 208784 |
| GRMZM2G108040 |  |  | 2 | -1 | 208,942,939 | 208,943,796 |  | 270261 |
| GRMZM2G121868 |  |  | 2 | -1 | 208,974,900 | 208,977,568 |  | 302222 |
| GRMZM2G007324 |  | Uncharacterized protein | 2 | -1 | 208,999,148 | 209,001,435 |  | 326470 |
| GRMZM2G077845 |  | GIR1 | 2 | 1 | 209,010,511 | 209,011,342 |  | 337833 |
| GRMZM2G376432 |  |  | 2 | -1 | 209,010,512 | 209,014,024 |  | 337834 |
| GRMZM2G175718 |  | Uncharacterized protein | 2 | -1 | 209,018,645 | 209,025,333 |  | 345967 |
| GRMZM2G378106 |  | Indole-3-acetic acid amido synthetase | 2 | -1 | 209,110,460 | 209,113,041 |  | 437782 |
| GRMZM2G079067 |  |  | 2 | 1 | 209,119,338 | 209,119,949 |  | 446660 |
| GRMZM2G079082 |  |  | 2 | -1 | 209,124,511 | 209,128,692 |  | 451833 |
| GRMZM2G378121 |  | Uncharacterized protein | 2 | 1 | 209,131,619 | 209,132,616 |  | 458941 |
| GRMZM2G040230 |  | Metal ion binding protein | 2 | -1 | 209,195,758 | 209,223,851 |  | 523080 |
| GRMZM2G414002 |  |  | 2 | -1 | 209,248,084 | 209,263,370 |  | 575406 |
| GRMZM2G113512 |  | Uncharacterized protein | 2 | -1 | 209,278,354 | 209,283,777 |  | 605676 |
| GRMZM2G113696 | *TIF5A* | Eukaryotic translation initiation factor 5A | 2 | -1 | 209,289,645 | 209,292,956 |  | 616967 |
| GRMZM2G115817 |  | Circadian clock coupling factor ZGT | 2 | 1 | 209,300,195 | 209,304,000 |  | 627517 |
| GRMZM2G146292 |  | Uncharacterized protein | 2 | -1 | 209,337,124 | 209,345,160 |  | 664446 |
| GRMZM2G146416 |  | Uncharacterized protein | 2 | -1 | 209,349,729 | 209,354,569 |  | 677051 |
| GRMZM2G703354 |  |  | 2 | -1 | 209,470,409 | 209,472,160 |  | 797731 |
| GRMZM2G084984 |  | Uncharacterized protein | 4 | 1 | 171,638,282 | 171,652,162 |  | 797559 |
| GRMZM2G102163 |  | Protein kinase superfamily proteinsssdf protein | 4 | 1 | 171,665,330 | 171,672,226 |  | 770511 |
| GRMZM2G102200 |  | Uncharacterized protein | 4 | -1 | 171,673,499 | 171,674,430 |  | 762342 |
| GRMZM2G402319 |  | Uncharacterized protein | 4 | 1 | 171,674,702 | 171,676,783 |  | 761139 |
| GRMZM2G102216 |  | Putative glutathione S-transferase family proteinssd protein | 4 | -1 | 171,676,838 | 171,678,581 |  | 759003 |
| GRMZM2G102230 |  | 60S ribosomal protein L23s proteindf | 4 | -1 | 171,688,353 | 171,692,167 |  | 747488 |
| GRMZM2G102815 |  | (Csu525(RpL17)), mRNA | 4 | 1 | 171,691,584 | 171,694,852 |  | 744257 |
| GRMZM2G099097 |  | Uncharacterized protein | 4 | 1 | 171,730,311 | 171,735,318 |  | 705530 |
| GRMZM2G099297 |  | Uncharacterized protein | 4 | 1 | 171,770,786 | 171,772,540 |  | 665055 |
| GRMZM2G099376 |  | Uncharacterized protein | 4 | -1 | 171,778,900 | 171,780,935 |  | 656941 |
| GRMZM2G099420 |  | Uncharacterized protein | 4 | -1 | 171,783,871 | 171,785,676 |  | 651970 |
| GRMZM2G370815 |  | Uncharacterized protein | 4 | -1 | 171,789,366 | 171,791,813 |  | 646475 |
| GRMZM2G702697 |  | Uncharacterized protein | 4 | 1 | 171,801,352 | 171,802,121 |  | 634489 |
| GRMZM2G702699 |  | Uncharacterized protein | 4 | 1 | 171,804,365 | 171,814,541 |  | 631476 |
| AC189280.3_FG001 |  | Uncharacterized protein | 4 | 1 | 171,842,284 | 171,845,974 |  | 593557 |
| GRMZM2G466532 |  | Uncharacterized protein | 4 | -1 | 171,908,549 | 171,910,339 |  | 527292 |
| GRMZM2G466545 |  | Uncharacterized protein | 4 | 1 | 171,917,079 | 171,922,792 |  | 518762 |
| GRMZM2G164854 |  | Uncharacterized protein | 4 | -1 | 171,921,876 | 171,925,757 |  | 513965 |
| GRMZM2G034005 |  | Protein binding proteinss proteins | 4 | -1 | 172,010,354 | 172,019,014 |  | 425487 |
| GRMZM2G062084 |  | Uncharacterized protein | 4 | 1 | 172,055,498 | 172,060,332 |  | 380343 |
| GRMZM2G075336 |  | ATP-dependent transporter YFL028C; Putative ABC transporter family protein | 4 | 1 | 172,080,242 | 172,084,381 |  | 355599 |
| GRMZM2G075124 |  | Putative casein kinase family proteinss sprotein | 4 | 1 | 172,085,426 | 172,089,671 |  | 350415 |
| GRMZM2G023899 |  | Uncharacterized protein | 4 | 1 | 172,109,058 | 172,110,448 |  | 326783 |
| GRMZM2G140901 |  | Putative NAC domain transcription factor superfamily protein | 4 | 1 | 172,192,989 | 172,195,242 |  | 242852 |
| GRMZM2G107109 |  | Uncharacterized protein | 4 | 1 | 172,257,209 | 172,265,943 |  | 178632 |
| GRMZM2G140524 |  | Uncharacterized protein | 4 | 1 | 172,309,385 | 172,318,450 |  | 126456 |
| GRMZM2G171163 |  | Uncharacterized protein | 4 | 1 | 172,357,234 | 172,360,388 |  | 78607 |
| GRMZM2G027075 | *jmj4* | Uncharacterized protein | 4 | 1 | 172,430,260 | 172,439,306 | S4_172435841 | 5,581 |
| GRMZM2G027302 |  | Uncharacterized protein | 4 | -1 | 172,444,330 | 172,446,310 |  | 8489 |
| GRMZM2G364068 |  | Uncharacterized protein | 4 | -1 | 172,522,239 | 172,530,660 |  | 86398 |
| GRMZM2G130043 |  | Starch synthase V | 4 | -1 | 172,606,253 | 172,677,186 |  | 170412 |
| GRMZM2G130002 |  | Putative RING zinc finger and VWF domain family proteins protein | 4 | 1 | 172,685,051 | 172,689,834 |  | 249210 |
| GRMZM2G129979 |  | Putative G10 domain family protein | 4 | -1 | 172,692,344 | 172,695,319 |  | 256503 |
| GRMZM2G178398 |  | Uncharacterized protein | 4 | 1 | 172,748,163 | 172,754,261 |  | 312322 |
| GRMZM2G379005 |  | Putative GATA transcription factor family protein | 4 | -1 | 172,760,157 | 172,765,319 |  | 324316 |
| AC186156.3_FG005 |  | Uncharacterized protein | 4 | 1 | 172,794,270 | 172,794,881 |  | 358429 |
| AC186156.3_FG004 |  | Uncharacterized protein | 4 | 1 | 172,821,941 | 172822552 |  | 386100 |
| GRMZM2G162052 |  | Uncharacterized protein | 4 | 1 | 172,831,531 | 172,833,992 |  | 395690 |
| GRMZM2G162007 |  | Putative alcohol dehydrogenase superfamily proteins protein | 4 | -1 | 172,834,862 | 172,837,214 |  | 399021 |
| GRMZM2G108364 |  | PAP-specific phosphatase | 4 | 1 | 172,992,607 | 172,995,763 |  | 556766 |
| GRMZM2G410487 |  | Uncharacterized protein | 4 | -1 | 172,996,195 | 172,997,403 |  | 560354 |
| GRMZM2G108284 |  | Uncharacterized protein | 4 | 1 | 172,998,043 | 173,001,754 |  | 562202 |
| GRMZM2G108712 |  | Proliferating cell nuclear antigen | 4 | -1 | 173,033,396 | 173,035,374 |  | 597555 |
| GRMZM2G054007 |  | Uncharacterized protein | 4 | 1 | 173,177,172 | 173,177,669 |  | 741331 |
| GRMZM2G054012 |  | 40S ribosomal protein S30 | 4 | -1 | 173,177,749 | 173,179,720 |  | 741908 |
| GRMZM2G054065 |  | Uncharacterized protein | 4 | 1 | 173,181,274 | 173,182,106 |  | 745433 |
| GRMZM2G054076 |  | Chaperone protein dnaJ | 4 | -1 | 173,181,520 | 173,194,277 |  | 745679 |
| GRMZM2G054210 |  | Uncharacterized protein | 4 | -1 | 173,209,988 | 173,224,508 |  | 774147 |
| GRMZM2G126772 |  | Uncharacterized protein | 5 | 1 | 77,657,450 | 77,659,533 |  | 725,156 |
| GRMZM2G428393 |  |  | 5 | -1 | 77,663,317 | 77,666,150 |  | 718,539 |
| GRMZM2G405947 |  |  | 5 | 1 | 77,669,514 | 77,675,552 |  | 709,137 |
| GRMZM2G033523 |  |  | 5 | 1 | 77,700,111 | 77,701,507 |  | 683,182 |
| GRMZM2G010731 |  |  | 5 | 1 | 77,727,436 | 77,729,552 |  | 655,137 |
| GRMZM2G010779 |  | Vacuolar cation/proton exchanger 2 | 5 | 1 | 77,729,765 | 77,749,253 |  | 635,436 |
| GRMZM2G010920 |  | Uncharacterized protein | 5 | -1 | 77,748,895 | 77,751,969 |  | 632,720 |
| GRMZM2G010944 |  | Uncharacterized protein | 5 | -1 | 77,753,934 | 77,757,260 |  | 627,429 |
| GRMZM2G011456 |  | Uncharacterized protein | 5 | -1 | 77,757,345 | 77,777,262 |  | 607,427 |
| GRMZM2G097207 |  | Glucan endo-1,3-beta-glucosidase 6 | 5 | 1 | 77,916,981 | 77,922,073 |  | 462,616 |
| GRMZM2G097275 |  | Uncharacterized protein | 5 | 1 | 77,940,666 | 77,945,714 |  | 438,975 |
| GRMZM2G023798 |  | Uncharacterized protein | 5 | -1 | 77,990,308 | 77,998,801 |  | 385,888 |
| GRMZM2G435796 |  |  | 5 | -1 | 78,022,476 | 78,023,096 |  | 361,593 |
| GRMZM5G832780 |  |  | 5 | -1 | 78,129,898 | 78,131,652 |  | 253,037 |
| GRMZM2G425559 |  |  | 5 | 1 | 78,129,898 | 78,131,784 |  | 252,905 |
| GRMZM2G006937 |  | Uncharacterized protein | 5 | 1 | 78,255,163 | 78,310,499 |  | 74,190 |
| GRMZM2G102912 |  | AIG2-like protein | 5 | 1 | 78,380,304 | 78,381,593 |  | 3,096 |
| GRMZM2G102845 | *arf2o* | Auxin response factor 20 | 5 | -1 | 78,381,834 | 78,389,884 | S5_78384689 | 2,855 |
| GRMZM5G865367 |  |  | 5 | 1 | 78,519,893 | 78,520,683 |  | 135,204 |
| GRMZM2G322493 |  | Uncharacterized protein | 5 | 1 | 78,758,856 | 78,765,635 |  | 374,167 |
| GRMZM2G167741 |  | Uncharacterized protein | 5 | 1 | 78,772,058 | 78,780,292 |  | 387,369 |
| GRMZM2G410357 |  | Uncharacterized protein | 5 | 1 | 78,804,756 | 78,815,306 |  | 420,067 |
| GRMZM2G410393 |  | Putative uncharacterized protein | 5 | 1 | 78,819,803 | 78,826,612 |  | 435,114 |
| AC197118.3_FG005 |  | Antigenic determinant of rec-A protein | 5 | -1 | 78,820,119 | 78,821,393 |  | 435,430 |
| GRMZM2G133048 |  |  | 5 | 1 | 78,904,639 | 78,918,850 |  | 519,950 |
| GRMZM2G018686 |  | Putative HLH DNA-binding domain superfamily protein | 5 | -1 | 79,055,654 | 79,056,960 |  | 670,965 |
| AC212103.3_FG002 |  |  | 5 | 1 | 79,162,381 | 79,163,631 |  | 777,692 |
| AC193606.2_FG001 |  |  | 5 | -1 | 79,175,814 | 79,176,557 |  | 791,125 |
| GRMZM2G332749 |  |  | 5 | 1 | 79,183,135 | 79,188,028 |  | 798,446 |
| GRMZM2G319798 |  |  | 7 | -1 | 107,780,060 | 107,782,693 |  | 752,317 |
| GRMZM2G074672 |  | Protein CCC1 | 7 | 1 | 107,855,871 | 107,858,296 |  | 676,714 |
| GRMZM2G019183 |  | Uncharacterized protein | 7 | 1 | 107,926,112 | 107,930,789 |  | 604,221 |
| GRMZM2G043240 |  | Putative uncharacterized protein | 7 | -1 | 108,007,980 | 108,011,772 |  | 523,238 |
| AC234163.1_FG002 |  | Uncharacterized protein | 7 | 1 | 108,253,198 | 108,254,882 |  | 280,128 |
| GRMZM2G700188 |  | Uncharacterized protein | 7 | -1 | 108,281,609 | 108,388,756 |  | 146,254 |
| GRMZM2G153527 |  | Receptor kinase | 7 | -1 | 108,480,222 | 108,482,455 |  | 52,555 |
| GRMZM2G045638 |  | Uncharacterized protein | 7 | 1 | 108,534,749 | 108,535,532 | S7_108535010 | 261 |
| GRMZM2G169356 |  |  | 7 | -1 | 108,698,427 | 108,699,794 |  | 163417 |
| GRMZM2G126566 |  | Typical P-type R2R3 Myb protein | 7 | 1 | 108,787,033 | 108,790,136 |  | 252023 |
| GRMZM2G126507 |  | Uncharacterized protein | 7 | -1 | 108,843,243 | 108,846,250 |  | 308233 |
| GRMZM2G446171 |  |  | 7 | -1 | 109,047,698 | 109,054,636 |  | 512688 |
| GRMZM2G146225 |  | Uncharacterized protein | 7 | -1 | 109,056,610 | 109,058,446 |  | 521600 |
| GRMZM2G066876 |  |  | 7 | -1 | 109,149,609 | 109,158,470 |  | 614599 |
| AC217843.3_FG001 |  | Uncharacterized protein | 7 | -1 | 109,217,683 | 109,218,246 |  | 682673 |
| GRMZM2G092550 |  |  | 7 | 1 | 109,309,083 | 109,325,401 |  | 774073 |
| GRMZM2G092525 |  | Uncharacterized protein | 7 | -1 | 109,326,537 | 109,329,414 |  | 791527 |
| GRMZM2G700405 |  | Uncharacterized protein | 8 | 1 | 15,945,384 | 15,946,458 |  | 796970 |
| GRMZM2G700407 |  | Uncharacterized protein | 8 | 1 | 15,950,199 | 15,951,272 |  | 792156 |
| GRMZM2G048804 |  | Uncharacterized protein | 8 | 1 | 15,958,422 | 15,977,849 |  | 765579 |
| GRMZM2G048763 |  | Uncharacterized protein | 8 | -1 | 15,981,227 | 15,982,875 |  | 760553 |
| GRMZM2G083328 |  | Uncharacterized protein | 8 | -1 | 15,984,817 | 15,987,132 |  | 756296 |
| GRMZM2G083394 |  | Uncharacterized protein | 8 | -1 | 15,987,735 | 15,992,845 |  | 750583 |
| GRMZM2G588728 |  | Uncharacterized protein | 8 | -1 | 16,040,312 | 16,040,810 |  | 702618 |
| AC211687.3_FG009 |  | Uncharacterized protein | 8 | 1 | 16,040,396 | 16,040,806 |  | 702622 |
| AC212565.3_FG002 |  | Uncharacterized protein | 8 | -1 | 16,076,551 | 16,077,390 |  | 666038 |
| GRMZM5G869161 |  | Uncharacterized protein | 8 | 1 | 16,164,364 | 16,164,876 |  | 578552 |
| AC212565.3_FG001 | *H4C7* | Histone H4 | 8 | -1 | 16,166,029 | 16,166,694 |  | 576734 |
| GRMZM2G469298 |  | Uncharacterized protein | 8 | 1 | 16,219,186 | 16,246,258 |  | 497170 |
| GRMZM2G341010 |  | Uncharacterized protein | 8 | 1 | 16,321,295 | 16,335,574 |  | 407854 |
| GRMZM2G033017 |  | Uncharacterized protein | 8 | -1 | 16,352,457 | 16,353,551 |  | 389877 |
| GRMZM2G081848 |  | Uncharacterized protein | 8 | -1 | 16,422,190 | 16,431,362 |  | 312066 |
| GRMZM2G380414 |  | Ultraviolet-B-repressible protein | 8 | -1 | 16,443,989 | 16,444,752 |  | 298676 |
| GRMZM2G587368 |  | Uncharacterized protein | 8 | -1 | 16,545,140 | 16,549,180 |  | 194248 |
| GRMZM2G165354 |  | Uncharacterized protein | 8 | 1 | 16,632,417 | 16,640,500 |  | 102928 |
| GRMZM2G061187 |  | Uncharacterized protein | 8 | -1 | 16,690,779 | 16,693,942 |  | 49486 |
| GRMZM2G141216 |  | Putative RING zinc finger domain superfamily protein | 8 | 1 | 16,700,565 | 16,704,075 |  | 39353 |
| GRMZM2G143211 |  | Uncharacterized protein | 8 | 1 | 16,741,652 | 16,746,323 | S8_16743428 | 1776 |
| AC211474.3_FG006 |  | Uncharacterized protein | 8 | 1 | 16,747,527 | 16,754,983 |  | 4099 |
| GRMZM2G143258 |  | Uncharacterized protein | 8 | -1 | 16,755,280 | 16,757,481 |  | 11852 |
| GRMZM2G143274 |  | Putative MYB DNA-binding domain superfamily protein | 8 | -1 | 16,770,013 | 16,771,212 |  | 26585 |
| GRMZM2G143278 |  | Uncharacterized protein | 8 | -1 | 16,777,746 | 16,778,788 |  | 34318 |
| GRMZM2G127308 |  | Tryptophan aminotransferase | 8 | -1 | 16,850,861 | 16,855,009 |  | 107433 |
| GRMZM5G853988 |  | Uncharacterized protein | 8 | 1 | 16,905,840 | 16,906,421 |  | 162412 |
| GRMZM2G025997 |  | Putative RING zinc finger domain superfamily protein | 8 | 1 | 16,911,248 | 16,915,154 |  | 167820 |
| GRMZM2G026015 |  | Photosystem I reaction center subunit XI | 8 | 1 | 16,916,336 | 16,918,485 |  | 172908 |
| GRMZM2G025215 |  | Putative DUF1421 domain family protein | 8 | -1 | 16,994,084 | 16,999,444 |  | 250656 |
| GRMZM2G463493 |  | Putative leucine-rich repeat receptor protein kinase family protein | 8 | 1 | 17,058,192 | 17,062,569 |  | 314764 |
| GRMZM2G162928 |  | Uncharacterized protein | 8 | -1 | 17,066,051 | 17,071,072 |  | 322623 |
| GRMZM2G095595 |  | Uncharacterized protein | 8 | 1 | 17,175,395 | 17,180,489 |  | 431967 |
| GRMZM2G095655 |  | Uncharacterized protein | 8 | 1 | 17,188,772 | 17,190,196 |  | 445344 |
| GRMZM2G473147 |  | Uncharacterized protein | 8 | -1 | 17,242,891 | 17,244,208 |  | 499463 |
| GRMZM2G067675 |  | Putative leucine-rich repeat receptor-like protein kinase family protein | 8 | -1 | 17,301,645 | 17,306,811 |  | 558217 |
| GRMZM2G119230 |  | Uncharacterized protein | 8 | 1 | 17,370,948 | 17,371,391 |  | 627520 |
| GRMZM5G893381 |  | Putative DEAD-box ATP-dependent RNA helicase family protein | 8 | -1 | 17,374,877 | 17,377,796 |  | 631449 |
| GRMZM2G062069 |  | Uncharacterized protein | 8 | -1 | 17,380,160 | 17,383,660 |  | 636732 |
| GRMZM2G061969 |  | Phospholipase D | 8 | -1 | 17,388,792 | 17,393,555 |  | 645364 |
| GRMZM2G364129 |  | Uncharacterized protein | 8 | -1 | 17,395,313 | 17,398,611 |  | 651885 |
| GRMZM2G061932 |  | Lipase/lipooxygenase, PLAT/LH2 | 8 | 1 | 17,420,296 | 17,421,579 |  | 676868 |
| GRMZM2G061735 |  | Uncharacterized protein | 8 | -1 | 17,422,102 | 17,426,460 |  | 678674 |
| GRMZM2G021069 |  | Minichromosome maintenance protein | 8 | -1 | 110516644 | 110522956 |  | 766085 |
| GRMZM2G387227 |  | Cyclin superfamily protein, putative | 8 | 1 | 110586176 | 110588594 |  | 700447 |
| GRMZM2G303168 |  | Uncharacterized protein | 8 | -1 | 110596224 | 110597303 |  | 691738 |
| GRMZM2G130569 |  | Uncharacterized protein | 8 | 1 | 110612301 | 110612764 |  | 676277 |
| GRMZM2G039017 |  | Uncharacterized protein | 8 | 1 | 110619879 | 110621612 |  | 667429 |
| GRMZM2G150134 |  | Uncharacterized protein | 8 | 1 | 110686795 | 110688334 |  | 600707 |
| GRMZM2G327741 |  | Uncharacterized protein | 8 | 1 | 111,014,304 | 111,015,047 |  | 788,861 |
| GRMZM2G016655 |  | Uncharacterized protein | 8 | -1 | 111,043,262 | 111,050,129 |  | 753,779 |
| GRMZM2G072238 |  | Putative HLH DNA-binding domain superfamily protein | 8 |  | 111,192,460 | 111,199,551 |  | 604,357 |
| GRMZM2G333079 |  | Uncharacterized protein | 8 | -1 | 111,287,695 | 111,290,414 |  | 513,494 |
| GRMZM2G416308 |  | Putative prolin-rich extensin-like receptor protein kinase family protein | 8 | 1 | 111,624,795 | 111,630,247 |  | 173,661 |
| GRMZM2G025175 |  | Uncharacterized protein | 8 | -1 | 111,704,094 | 111,705,090 |  | 98,818 |
| GRMZM2G046537 |  | Rhomboid family protein | 8 | -1 | 111,722,040 | 111,730,019 |  | 73,889 |
| GRMZM2G157332 |  | Coiled-coil domain-containing protein 25 | 8 | -1 | 111,799,986 | 111,807,525 | S8_111803908 | 3,922 |
| GRMZM2G157564 |  | CER5 | 8 | -1 | 111,823,880 | 111,830,792 |  | 19,972 |
| GRMZM2G053882 |  | Uncharacterized protein | 8 | 1 | 111,864,621 | 111,872,240 |  | 60,713 |
| GRMZM2G362163 |  | Uncharacterized protein | 8 | -1 | 112,001,191 | 112,002,329 |  | 197,283 |
| GRMZM2G153569 |  | Elongation factor 1-delta 1 | 8 | -1 | 112,147,719 | 112,150,640 |  | 343,811 |
| GRMZM2G153552 |  | Uncharacterized protein | 8 | 1 | 112,176,065 | 112,177,057 |  | 372,157 |
| GRMZM2G050286 |  | AGP20 | 8 | -1 | 112,310,806 | 112,311,880 |  | 506,898 |
| GRMZM2G700603 |  | Uncharacterized protein | 8 | 1 | 112,378,878 | 112,381,753 |  | 574,970 |
| GRMZM2G308999 |  | Uncharacterized protein | 8 | 1 | 112,463,421 | 112,466,262 |  | 659,513 |
| GRMZM5G854731 |  | Cyclin-dependent kinase inhibitor 2 | 8 | 1 | 112,496,249 | 112,497,984 |  | 692,341 |
| GRMZM2G179679 |  | Uncharacterized protein | 8 | 1 | 112,530,662 | 112,532,375 |  | 726,754 |
| GRMZM2G180668 |  | DNA-binding WRKY | 8 | 1 | 123,810,520 | 123,811,466 |  | 623259 |
| GRMZM2G104283 |  | Uncharacterized protein | 8 | 1 | 123,844,264 | 123,848,418 |  | 586307 |
| GRMZM2G350023 |  | Uncharacterized protein | 8 | 1 | 123,899,080 | 123,900,573 |  | 534152 |
| GRMZM2G136369 |  | Putative homeodomain-like transcription factor superfamily protein | 8 | 1 | 123,906,214 | 123,912,675 |  | 522050 |
| GRMZM5G891056 |  | Uncharacterized protein | 8 | 1 | 123,944,098 | 123,967,891 |  | 466834 |
| AC233864.1_FG002 |  | Uncharacterized protein | 8 | 1 | 124,013,979 | 124,014,615 |  | 420110 |
| GRMZM5G892365 |  | Uncharacterized protein | 8 | 1 | 124,033,198 | 124,034,311 |  | 400414 |
| AC233864.1_FG009 |  | Uncharacterized protein | 8 | -1 | 124,042,906 | 124,043,298 |  | 391427 |
| AC233864.1_FG014 |  | Uncharacterized protein | 8 | -1 | 124,086,876 | 124,091,045 |  | 343680 |
| GRMZM2G115346 |  | Uncharacterized protein | 8 | -1 | 124,144,399 | 124,145,855 |  | 288870 |
| GRMZM2G415891 |  | Uncharacterized protein | 8 | 1 | 124,164,647 | 124,165,273 |  | 269452 |
| GRMZM2G115364 |  | Uncharacterized protein | 8 | -1 | 124,165,959 | 124,168,715 |  | 266010 |
| GRMZM2G169236 |  | Uncharacterized protein | 8 | -1 | 124,188,697 | 124,190,213 |  | 244512 |
| GRMZM2G162884 |  | Uncharacterized protein | 8 | 1 | 124,208,874 | 124,213,413 |  | 221312 |
| GRMZM2G084477 |  | MPPN domain containing protein | 8 | -1 | 124,223,433 | 124,225,424 |  | 209301 |
| GRMZM2G127844 |  | Uncharacterized protein | 8 | -1 | 124,282,351 | 124,287,196 |  | 147529 |
| GRMZM2G135381 |  | Putative GATA transcription factor family protein | 8 | 1 | 124,357,134 | 124,359,274 |  | 75451 |
| GRMZM2G038082 |  | Uncharacterized protein | 8 | -1 | 124,433,434 | 124,434,146 |  | 579 |
| GRMZM2G330693 |  | Uncharacterized protein | 8 | 1 | 124,434,479 | 124,435,152 | S8_124434725 | 246 |
| GRMZM5G802899 |  | Uncharacterized protein | 8 | 1 | 124,437,928 | 124,439,232 |  | 3203 |
| GRMZM2G173700 |  | Uncharacterized protein | 8 | -1 | 124,457,224 | 124,464,067 |  | 22499 |
| GRMZM2G173710 |  | Uncharacterized protein | 8 | -1 | 124,485,354 | 124,490,167 |  | 50629 |
| GRMZM2G071223 |  | Uncharacterized protein | 8 | 1 | 124,583,741 | 124,586,190 |  | 149016 |
| GRMZM2G071339 |  | Uncharacterized protein | 8 | 1 | 124,587,539 | 124,589,539 |  | 152814 |
| GRMZM2G163546 |  | Uncharacterized protein | 8 | 1 | 124,622,026 | 124,623,523 |  | 187301 |
| GRMZM2G058690 |  | Uncharacterized protein | 8 | -1 | 124,645,482 | 124,648,145 |  | 210757 |
| GRMZM2G058745 |  | Uncharacterized protein | 8 | 1 | 124,652,438 | 124,657,923 |  | 217713 |
| GRMZM2G109842 | *PRO2* | Profilin-2 | 8 | -1 | 124,707,856 | 124,708,953 |  | 273131 |
| GRMZM2G106819 |  | Uncharacterized protein | 8 | 1 | 124,796,660 | 124,797,582 |  | 361935 |
| GRMZM2G106917 |  | Uncharacterized protein | 8 | 1 | 124,799,105 | 124,810,762 |  | 364380 |
| GRMZM2G093418 |  | Harpin-induced protein | 8 | 1 | 124,839,636 | 124,840,524 |  | 404911 |
| GRMZM2G093404 |  | CCCH transcription factor | 8 | 1 | 124,841,672 | 124,843,159 |  | 406947 |
| GRMZM2G095807 |  | Uncharacterized protein | 8 | -1 | 124,847,602 | 124,849,378 |  | 412877 |
| GRMZM2G082707 |  | 50S ribosomal protein L20 | 8 | -1 | 124,930,000 | 124,932,025 |  | 495275 |
| GRMZM2G319747 |  | Proteasome maturation factor UMP1 family protein | 8 | -1 | 124,936,785 | 124,942,287 |  | 502060 |
| GRMZM2G015433 |  | Putative WRKY DNA-binding domain superfamily protein | 8 | -1 | 124,950,950 | 124,952,109 |  | 516225 |
| GRMZM2G353822 |  | Uncharacterized protein | 8 | -1 | 125,007,017 | 125,008,185 |  | 572292 |
| GRMZM5G855375 |  | Uncharacterized protein | 8 | 1 | 125,007,024 | 125,008,185 |  | 572299 |
| GRMZM2G052200 |  | Uncharacterized protein | 8 | -1 | 125,008,668 | 125,012,511 |  | 573943 |
| GRMZM2G408989 |  | ER lumen protein retaining receptor | 8 | -1 | 125,111,092 | 125,114,558 |  | 676367 |
| AC205471.4_FG003 |  | Uncharacterized protein | 8 | 1 | 125,180,331 | 125,182,094 |  | 745606 |
| AC205471.4_FG008 |  | Uncharacterized protein | 8 | -1 | 125,196,525 | 125,197,922 |  | 761800 |
| AC205471.4_FG007 |  | Uncharacterized protein | 8 | -1 | 125,225,855 | 125,226,494 |  | 791130 |
| GRMZM2G018375 | *THI1-1* | Thiamine thiazole synthase 1, chloroplastic | 8 | -1 | 138,142,847 | 138,144,617 |  | 794,332 |
| AC199315.4_FG001 |  | Protein transport protein Sec61 beta subunit | 8 | 1 | 138,225,306 | 138,225,551 |  | 713,398 |
| AC199315.4_FG002 |  | Uncharacterized protein | 8 | 1 | 138,255,217 | 138,255,384 |  | 683,565 |
| GRMZM2G164341 |  | Putative HLH DNA-binding domain superfamily protein | 8 | -1 | 138,262,239 | 138,264,389 |  | 674,560 |
| GRMZM2G142984 |  | Uncharacterized protein | 8 | -1 | 138,315,538 | 138,326,465 |  | 612,484 |
| GRMZM2G132577 |  | Uncharacterized protein | 8 | -1 | 138,390,391 | 138,391,121 |  | 547,828 |
| GRMZM2G368556 |  | Uncharacterized protein | 8 | -1 | 138,431,180 | 138,431,962 |  | 506,987 |
| GRMZM2G700683 |  | Uncharacterized protein | 8 | 1 | 138,510,107 | 138,515,042 |  | 423,907 |
| GRMZM2G106479 |  | Uncharacterized protein | 8 | -1 | 138,519,520 | 138,524,207 |  | 414,742 |
| GRMZM2G136765 |  | Uncharacterized protein | 8 | 1 | 138,556,689 | 138,562,724 |  | 376,225 |
| GRMZM2G409343 |  | Uncharacterized protein | 8 | 1 | 138,645,560 | 138,647,354 |  | 291,595 |
| GRMZM5G852338 |  | Uncharacterized protein | 8 | 1 | 138,647,887 | 138,650,204 |  | 288,745 |
| GRMZM2G170628 |  | Uncharacterized protein | 8 | -1 | 138,657,602 | 138,660,155 |  | 278,794 |
| GRMZM2G170632 |  | RNA-binding protein | 8 | -1 | 138,667,667 | 138,671,356 |  | 267,593 |
| GRMZM2G116083 |  | Uncharacterized protein | 8 | 1 | 138,789,602 | 138,795,274 |  | 143,675 |
| GRMZM2G013448 |  | Uncharacterized protein | 8 | 1 | 138,861,203 | 138,862,890 |  | 76,059 |
| GRMZM5G851965 |  | Uncharacterized protein | 8 | 1 | 138,868,954 | 138,870,495 |  | 68,454 |
| GRMZM2G012966 | *lcyE* | Lycopene epsilon cyclase1 | 8 | 1 | 138,882,594 | 138,889,812 |  | 49,137 |
| GRMZM2G165428 |  | Putative leucine-rich repeat protein kinase family protein | 8 | 1 | 138,909,363 | 138,913,061 |  | 25,888 |
| GRMZM2G463133 |  | Putative HLH DNA-binding domain superfamily protein | 8 | -1 | 138,938,542 | 138,943,955 | S8_138938949 | 407 |
| AC196426.3_FG007 |  | Uncharacterized protein | 8 | -1 | 139,014,642 | 139,020,725 |  | 75693 |
| GRMZM2G096655 |  | Uncharacterized protein | 8 | 1 | 139,104,748 | 139,106,331 |  | 165799 |
| GRMZM2G395771 |  | Uncharacterized protein | 8 | 1 | 139,138,199 | 139,140,971 |  | 199250 |
| GRMZM2G096764 |  | Uncharacterized protein | 8 | -1 | 139,143,564 | 139,151,103 |  | 204615 |
| GRMZM2G050553 |  | Uncharacterized protein | 8 | 1 | 139,286,853 | 139,300,161 |  | 347904 |
| GRMZM2G027333 |  | Uncharacterized protein | 8 | 1 | 139,342,590 | 139,346,470 |  | 403641 |
| GRMZM2G142705 |  | Uncharacterized protein | 8 | -1 | 139,450,959 | 139,452,582 |  | 512010 |
| GRMZM2G114895 |  | LIN1 protein | 8 | 1 | 139,487,349 | 139,489,298 |  | 548400 |
| GRMZM2G420119 |  | Uncharacterized protein | 8 | -1 | 139,592,349 | 139,595,685 |  | 653400 |
| GRMZM2G046037 |  | Uncharacterized protein | 8 | 1 | 139,630,135 | 139,631,894 |  | 691186 |
| GRMZM2G054900 |  | Putative calmodulin-binding family protein | 8 | 1 | 139,711,000 | 139,713,137 |  | 772051 |
| GRMZM2G386430 |  | Uncharacterized protein | 10 | 1 | 133877805 | 133886124 |  | 764857 |
| GRMZM2G404375 |  | Uncharacterized protein | 10 | -1 | 133982300 | 133985927 |  | 665054 |
| GRMZM2G404367 |  | Uncharacterized protein | 10 | -1 | 133989048 | 133990408 |  | 660573 |
| GRMZM2G102860 |  | Uncharacterized protein | 10 | 1 | 133992324 | 133993013 |  | 657968 |
| GRMZM2G126665 |  | Uncharacterized protein | 10 | 1 | 134032517 | 134034788 |  | 616193 |
| GRMZM2G427087 |  | Putative homeodomain-like transcription factor superfamily protein | 10 | 1 | 134039237 | 134040599 |  | 610382 |
| GRMZM2G126742 |  | Uncharacterized protein | 10 | -1 | 134043229 | 134046251 |  | 604730 |
| GRMZM2G427097 |  | Glutamate dehydrogenase | 10 | 1 | 134053442 | 134057895 |  | 593086 |
| GRMZM2G427106 |  | Uncharacterized protein | 10 | -1 | 134057764 | 134059399 |  | 591582 |
| GRMZM2G421212 |  | Uncharacterized protein | 10 | -1 | 134094157 | 134098692 |  | 552289 |
| GRMZM2G134523 |  | Uncharacterized protein | 10 | -1 | 134251243 | 134254824 |  | 396157 |
| GRMZM2G134517 |  | Uncharacterized protein | 10 | -1 | 134255159 | 134255926 |  | 395055 |
| GRMZM2G016819 |  | Uncharacterized protein | 10 | 1 | 134399292 | 134400918 |  | 250063 |
| GRMZM2G016939 |  | Protein kinase G11A protein | 10 | 1 | 134410371 | 134414070 |  | 236911 |
| GRMZM2G017789 |  | Ubiquitin carboxyl-terminal hydrolase isozyme L3 protein | 10 | 1 | 134414639 | 134418345 |  | 232636 |
| GRMZM2G027173 |  | ELMO domain-containing protein 2 protein | 10 | -1 | 134431102 | 134434923 |  | 216058 |
| GRMZM2G026983 |  | Uncharacterized protein | 10 | -1 | 134436742 | 134439351 |  | 211630 |
| GRMZM2G092599 |  | Uncharacterized protein | 10 | -1 | 134439511 | 134441032 |  | 209949 |
| GRMZM5G805382 |  | Uncharacterized protein | 10 | -1 | 134457411 | 134458785 |  | 192196 |
| GRMZM2G063972 |  | Uncharacterized protein | 10 | 1 | 134495450 | 134504022 |  | 146959 |
| GRMZM2G018631 |  | Putative RING zinc finger domain superfamily protein | 10 | -1 | 134566707 | 134567642 |  | 83339 |
| GRMZM2G322586 |  | Uncharacterized protein | 10 | 1 | 134569581 | 134576295 |  | 74686 |
| GRMZM2G322582 |  | Putative WAK-related receptor-like protein kinase family protein | 10 | 1 | 134583763 | 134585981 |  | 65000 |
| GRMZM2G018485 |  | Uncharacterized protein | 10 | -1 | 134600335 | 134602107 |  | 48874 |
| GRMZM2G018464 |  | Putative G10 domain family protein | 10 | 1 | 134606660 | 134612036 |  | 38945 |
| GRMZM2G018314 |  | Uncharacterized protein | 10 | 1 | 134647347 | 134652537 | S10_134650981 | 1556 |
| GRMZM2G322506 |  | Uncharacterized protein | 10 | 1 | 134653914 | 134659406 |  | 2933 |
| GRMZM5G887529 |  | Uncharacterized protein | 10 | -1 | 134707390 | 134707643 |  | 56409 |
| GRMZM2G018027 |  | Uncharacterized protein | 10 | -1 | 134717099 | 134718846 |  | 66118 |
| GRMZM2G072121 |  | Uncharacterized protein | 10 | 1 | 134791598 | 134804631 |  | 140617 |
| GRMZM2G149178 | *H4C7* | Histone H4 | 10 | -1 | 134805431 | 134806076 |  | 154450 |
| GRMZM2G481249 |  | Uncharacterized protein | 10 | 1 | 134827823 | 134828576 |  | 176842 |
| GRMZM2G181030 |  | Putative MYB DNA-binding domain superfamily protein protein | 10 | -1 | 134830171 | 134832693 |  | 179190 |
| GRMZM2G321239 |  | CDPK protein; Putative calcium-dependent protein kinase family protein | 10 | 1 | 134917888 | 134922506 |  | 266907 |
| GRMZM2G097813 |  | Uncharacterized protein | 10 | 1 | 134969148 | 134971067 |  | 318167 |
| GRMZM2G097848 |  | Uncharacterized protein | 10 | -1 | 134974316 | 134977168 |  | 323335 |
| GRMZM2G031280 |  | Putative RING zinc finger domain superfamily protein | 10 | -1 | 134991961 | 134992953 |  | 340980 |
| GRMZM2G031326 |  | Nucleolar complex protein 4 | 10 | -1 | 134999391 | 135006443 |  | 348410 |
| GRMZM2G031453 |  | Uncharacterized protein | 10 | 1 | 135008283 | 135011071 |  | 357302 |
| GRMZM2G322634 |  | Uncharacterized protein | 10 | -1 | 135044840 | 135048155 |  | 393859 |
| AC233979.1_FG006 |  | Uncharacterized protein | 10 | -1 | 135056126 | 135058358 |  | 405145 |
| GRMZM5G814481 |  | Uncharacterized protein | 10 | -1 | 135125968 | 135126659 |  | 474987 |
| AC233979.1_FG007 |  | Uncharacterized protein | 10 | -1 | 135130731 | 135131021 |  | 479750 |
| AC233979.1_FG008 |  | Uncharacterized protein | 10 | -1 | 135135344 | 135135658 |  | 484363 |
| AC233979.1_FG009 |  | Uncharacterized protein | 10 | 1 | 135164262 | 135169386 |  | 513281 |
| AC233979.1_FG010 |  | Uncharacterized protein | 10 | 1 | 135171168 | 135172765 |  | 520187 |
| AC233979.1_FG011 |  | Putative RING zinc finger domain superfamily protein | 10 | 1 | 135174868 | 135175986 |  | 523887 |
| GRMZM2G413796 |  | Uncharacterized protein | 10 | 1 | 135273676 | 135318895 |  | 622695 |
| GRMZM2G113818 |  | Uncharacterized protein | 10 | 1 | 135325713 | 135327992 |  | 674732 |
| GRMZM2G113800 |  | Uncharacterized protein | 10 | 1 | 135330047 | 135331862 |  | 679066 |
| GRMZM2G413774 |  | Uncharacterized protein | 10 | 1 | 135364950 | 135393105 |  | 713969 |
| GRMZM2G086474 |  | Putative HLH DNA-binding domain superfamily protein | 10 | 1 | 135,677,365 | 135,680,963 |  | 326612 |
| GRMZM2G086403 |  | PLATZ transcription factors proteidn | 10 | -1 | 135,685,281 | 135,687,959 |  | 319616 |
| GRMZM2G173429 |  | Uncharacterized protein | 10 | 1 | 135,800,706 | 135,802,992 |  | 204583 |
| GRMZM2G162640 |  | Uncharacterized protein | 10 | 1 | 135,910,599 | 135,912,870 |  | 94705 |
| GRMZM2G397684 |  | Putative RING zinc finger domain superfamily protein | 10 | 1 | 136,006,849 | 136,007,871 | S10_136007575/ S10_136007578 | 293 |
| GRMZM2G098676 |  | Putative DUF604-domain containing/glycosyltransferase-related family protein | 10 | 1 | 136,016,690 | 136,019,623 |  | 9112 |
| GRMZM2G152135 | *crtRB1* | Beta-carotene hydroxylase 1 | 10 | -1 | 136,057,100 | 136,060,219 |  | 49522 |
| GRMZM2G454399 |  | Uncharacterized protein | 10 | -1 | 136,064,677 | 136,068,220 |  | 57099 |
| GRMZM5G894619 |  | Uncharacterized protein | 10 | -1 | 136,083,487 | 136,086,305 |  | 75909 |
| GRMZM2G040673 |  | Plant-specific domain TIGR01568 family protein | 10 | -1 | 136,103,462 | 136,105,185 |  | 95884 |
| GRMZM2G040359 |  | Uncharacterized protein | 10 | -1 | 136,105,398 | 136,107,763 |  | 97820 |
| GRMZM2G016477 |  | Putative leucine-rich repeat receptor-like protein kinase family protein | 10 | -1 | 136,114,579 | 136,118,747 |  | 107001 |
| GRMZM2G105855 |  | Uncharacterized protein | 10 | -1 | 136,179,354 | 136,184,210 |  | 171776 |
| GRMZM2G305146 |  | Uncharacterized protein | 10 | 1 | 136,245,381 | 136,247,528 |  | 237803 |
| GRMZM2G134703 |  | Putative cytochrome P450 superfamily proteins proteind | 10 | -1 | 136,271,138 | 136,273,515 |  | 263560 |
| GRMZM2G414915 |  | Putative subtilase family proteins protein | 10 | 1 | 136,292,132 | 136,296,887 |  | 284554 |
| GRMZM2G702514 |  | Uncharacterized protein | 10 | -1 | 136,325,395 | 136,339,339 |  | 317817 |
| AC199382.3_FG005 |  | Uncharacterized protein | 10 | 1 | 136,339,578 | 136,340,304 |  | 332000 |
| GRMZM2G018018 |  | Uncharacterized protein | 10 | -1 | 136,435,256 | 136,440,939 |  | 427678 |
| GRMZM2G424112 |  | Uncharacterized protein | 10 | -1 | 136,478,132 | 136,478,844 |  | 470554 |
| GRMZM2G006212 |  | Putative MATE efflux family protein | 10 | -1 | 136,615,140 | 136,617,256 |  | 607562 |
| GRMZM2G077082 |  | Uncharacterized protein | 10 | 1 | 136,697,887 | 136,699,626 |  | 690309 |
| GRMZM2G077069 |  | Uncharacterized protein | 10 | -1 | 136,700,998 | 136,704,479 |  | 693420 |
| GRMZM2G077036 |  | Uncharacterized protein | 10 | 1 | 136,709,655 | 136,713,380 |  | 702077 |
| AC199370.4_FG006 |  | Uncharacterized protein | 10 | -1 | 136,742,880 | 136,743,326 |  | 735302 |
| GRMZM5G896748 |  | Uncharacterized protein | 10 | 1 | 136,742,977 | 136,743,532 |  | 735399 |
| GRMZM2G148467 |  | Squamosa promoter-binding protein-like (SBP domain) transcription factor family protein | 10 | -1 | 139078940 | 139081895 |  | 795699 |
| GRMZM2G124965 |  | Uncharacterized protein | 10 | 1 | 139115338 | 139120886 |  | 756708 |
| GRMZM2G125023 | *TIP2-3* | Aquaporin TIP2-3 | 10 | 1 | 139122197 | 139125216 |  | 752378 |
| GRMZM2G392040 |  | Uncharacterized protein | 10 | 1 | 139191682 | 139193279 |  | 684315 |
| GRMZM2G311059 |  | Putative MYB DNA-binding domain superfamily protein | 10 | -1 | 139838952 | 139839963 |  | 37631 |
| GRMZM2G007899 |  | Uncharacterized protein | 10 | -1 | 139840305 | 139841290 |  | 36304 |
| GRMZM2G303768 |  | Putative leucine-rich repeat receptor-like protein kinase family protein | 10 | 1 | 139852918 | 139855428 |  | 22166 |
| GRMZM2G004709 |  | EREBP-4 like protein protein | 10 | -1 | 139858081 | 139861295 |  | 16299 |
| GRMZM2G080516 |  | AP2-EREBP transcription factor | 10 | -1 | 139875910 | 139877865 | S10_139877594 | 271 |
| GRMZM2G129006 |  | Uncharacterized protein | 10 | -1 | 139934291 | 139935167 |  | 56697 |
| GRMZM2G555422 |  | Uncharacterized protein | 10 | -1 | 139959580 | 139962869 |  | 81986 |
| GRMZM2G128057 |  | Uncharacterized protein | 10 | 1 | 139976216 | 139979369 |  | 98622 |
| GRMZM2G128074 |  | Uncharacterized protein | 10 | -1 | 139980303 | 139984144 |  | 102709 |
| GRMZM2G128078 |  | Uncharacterized protein | 10 | -1 | 139984959 | 139987580 |  | 107365 |
| GRMZM2G128092 |  | Uncharacterized protein | 10 | -1 | 139995082 | 140003086 |  | 117488 |
| GRMZM2G081557 |  | Putative MYB DNA-binding domain superfamily protein protein | 10 | -1 | 140048664 | 140050182 |  | 171070 |
| GRMZM2G119383 |  | Uncharacterized protein | 10 | -1 | 140142031 | 140143150 |  | 264437 |
| GRMZM2G119370 |  | Uncharacterized protein | 10 | 1 | 140145782 | 140146639 |  | 268188 |
| GRMZM2G150107 |  | Uncharacterized protein | 10 | 1 | 140170361 | 140170979 |  | 292767 |
| GRMZM2G397848 |  | Uncharacterized protein | 10 | -1 | 140184269 | 140185171 |  | 306675 |
| GRMZM2G097618 |  | Uncharacterized protein | 10 | -1 | 140185478 | 140185945 |  | 307884 |
| GRMZM2G097628 |  | Uncharacterized protein | 10 | -1 | 140186581 | 140187542 |  | 308987 |
| GRMZM2G097636 |  | Putative MYB DNA-binding domain superfamily protein | 10 | -1 | 140190031 | 140191484 |  | 312437 |
| GRMZM2G097638 |  | Putative MYB DNA-binding domain superfamily protein | 10 | -1 | 140193073 | 140194565 |  | 315479 |
| GRMZM2G104989 |  | Putative RNA recognition motif containing family protein | 10 | -1 | 140223325 | 140225120 |  | 345731 |
| AC198515.3_FG011 |  | Uncharacterized protein | 10 | 1 | 140268585 | 140271261 |  | 390991 |
| GRMZM2G017752 |  | Uncharacterized protein | 10 | 1 | 140289208 | 140290291 |  | 411614 |
| GRMZM2G017647 |  | Uncharacterized protein | 10 | -1 | 140305834 | 140311238 |  | 428240 |
| GRMZM2G159389 |  | Uncharacterized protein | 10 | -1 | 140344785 | 140347607 |  | 467191 |
| GRMZM2G458283 |  | Putative translation elongation factor Tu family protein | 10 | -1 | 140351382 | 140355980 |  | 473788 |
| GRMZM5G872750 |  | Uncharacterized protein | 10 | -1 | 140357577 | 140358332 |  | 479983 |
| GRMZM2G364060 |  | SC3 protein protein | 10 | -1 | 140486168 | 140496625 |  | 608574 |
| AC192330.1_FG004 |  | Uncharacterized protein | 10 | 1 | 140597198 | 140599033 |  | 719604 |
| AC208110.2_FG001 |  | Putative WRKY DNA-binding domain superfamily protein | 10 | 1 | 140656185 | 140659057 |  | 778591 |

**Note:** Genes and associated information retrieved from maizeGDB.org and gramene.org. Physical positions of SNPs and coordinates of genes given according to B73 RefGen_2.
